# Cause of Death and Risk of Death for People Living With HIV Admitted to Hospital: A Systematic Review and Meta‐Analysis

**DOI:** 10.1002/jia2.70134

**Published:** 2026-06-09

**Authors:** Rachael M. Burke, Nadia Sabet, Jayne Ellis, Hannah M. Rickman, Ajay Rangaraj, David S. Lawrence, Joseph N. Jarvis, Jane Falconer, Lillian Tugume, Rebecca H. Berhanu, Nathan Ford, Peter MacPherson

**Affiliations:** ^1^ Faculty of Infectious and Tropical Disease London School of Hygiene and Tropical Medicine London UK; ^2^ Malawi Liverpool Wellcome Research Programme Blantyre Malawi; ^3^ Perinatal HIV Research Unit (PHRU) University of the Witwatersrand Johannesburg South Africa; ^4^ Infectious Diseases Institute Makerere University Kampala Uganda; ^5^ Department of HIV, Viral Hepatitis and Sexually Transmitted Infections World Health Organization Geneva Switzerland; ^6^ Botswana Harvard Health Partnership Gaborone Botswana; ^7^ School of Pathology, Faculty of Health Sciences University of the Witwatersrand Johannesburg South Africa; ^8^ Library, Archive and Open Research Services London School of Hygiene and Tropical Medicine London UK; ^9^ Division of Infectious Diseases Department of Medicine, Vanderbilt University Medical Center Nashville Tennessee USA; ^10^ Centre for Integrated Data and Epidemiological Research, School of Public Health and Family Medicine, Faculty of Health Sciences University of Cape Town Cape Town South Africa; ^11^ School of Health and Wellbeing University of Glasgow Glasgow UK

**Keywords:** advanced HIV disease, death, epidemiology, hospitalization, opportunistic infections, tuberculosis

## Abstract

**Introduction:**

Despite increasing access to HIV treatment and care, HIV‐associated deaths remain high. We aimed to summarize global and regional trends in risk and causes of death among people living with HIV (PLHIV) admitted to hospital.

**Methods:**

We conducted a systematic search across eight databases on 23 April 2023, identifying studies that reported cause of hospital admission or death among hospitalized PLHIV from first January 2014 onwards. We extracted data on age, geographical region, type of ward, antiretroviral treatment use, CD4 cell count, risk of death, cause of death and method of ascertainment of cause of death. We grouped studies into mutually exclusive groups: adults in medical wards by world region; adults in intensive care; and children. We used a Bayesian multilevel meta‐regression model to pool data on causes of death. We additionally estimated temporal trends in risk of death among hospitalized PLHIV between 2000 and 2023.

**Results:**

We identified 67 studies (59,013 participants) reporting risk of death among hospitalized PLHIV between 2014 and 2023. The overall risk of in‐hospital death was 16% (95% credible interval [CrI]: 8%–27%). Mortality risk was highest among adults in Africa (19%, 95% CI: 15%–24%) and adults in intensive care units (44%, 95% CI: 34%–55%). Among 40 studies reporting cause of death in 6,838 participants, AIDS‐related conditions predominated (72% of deaths, 95% CrI: 57%–85%), including tuberculosis deaths (27% of deaths, 95% CrI: 15%–40%). Bacterial infections were the second leading cause of death (25% of deaths, 95% CrI: 9%–47%). There was no strong evidence of risk of death changing between 2000 and 2023 (−2.2 percentage point decrease, 95% CrI −16.1 to +17.0 percentage points).

**Conclusions:**

Despite advances in HIV treatment, AIDS‐related illnesses and bacterial infections remain the leading causes of in‐hospital death among PLHIV. Our analysis reveals that in most regions, the risk of death for hospitalized PLHIV has remained largely unchanged in the past 23 years. These findings underscore the critical need to prioritize high‐quality hospital care for opportunistic infections to reduce AIDS‐related deaths.

## 1 Introduction

Advanced HIV disease remains a persistent problem. Since around 1995 in Europe and North America, and 2003 in other regions, access to HIV care, including antiretroviral treatment (ART), has increased substantially [1]. While improved ART‐access has saved millions of years of life [2], approximately a third of people living with HIV (PLHIV) still present to healthcare with advanced HIV disease (CD4<200 cells/mm^3^) [3]. Hospitalization remains common among PLHIV, who experience a very high risk of death during hospital admission and in the weeks following discharge [4].

Understanding the range of causes of illness and death among hospitalized PLHIV is crucial for informing approaches to opportunistic infection prophylaxis and early detection strategies for life‐threatening communicable and non‐communicable diseases in PLHIV. A 2015 systematic review identified AIDS‐related illness and bacterial infections as major causes of both hospital admissions and deaths in all age groups and world regions [5]. Since then, the global adoption of the recommendation to “Treat All” using highly effective integrase‐inhibitor‐based ART regimens has been introduced [6], and this may have changed the risk and causes of death among hospitalized PLHIV. There has also been a rise in the number of deaths due to non‐communicable conditions in all regions of the world [7], including among PLHIV [8].

We aimed to systematically review the risk and causes of death among hospitalized PLHIV since 2014, and to investigate changes over time since 2000.

## 2 Methods

### 2.1 Search Strategy and Selection Criteria

We conducted a systematic review to inform the 2025 WHO Advanced HIV Disease Guidelines, reported according to PRISMA guidelines. Searches were conducted by an information specialist (JF), and selection processes have been described in detail previously [9]. We searched eight databases (Ovid Medline ALL, Ovid Embase Classic+Embase, Ovid Global Health, EBSCOhost CINAHL Complete, EBSCOhost Africa‐Wide Information, Clarivate Analytics Web of Science Core Content, Clarivate Analytics Web of Science SciELO and Global Index Medicus) on 26 April 2023 for terms related to HIV and to either hospital admission or death. Articles were uploaded into the Rayyan software for screening. One author (RMB) initially removed clearly irrelevant records based on title‐abstract review; a second title‐abstract review of possibly relevant records was performed independently in duplicate, with two additional reviewers (RMB, JE, NS). Relevant records were reviewed at full text by two authors (two of RMB, JE, NS). Additional relevant studies were identified through reference list screening and expert opinion.

We included studies reporting causes of death among hospitalized PLHIV and studies reporting risk of in‐hospital death where cause of admission was reported. We excluded studies of pre‐selected groups (e.g. PLHIV admitted with cough or stroke) to maintain generalizability to general hospital settings. We included studies using various methods of death ascertainment, including medical record reviews and autopsy studies (complete diagnostic or minimally invasive). To provide updated evidence following a previous systematic review, we included studies where participants were admitted to hospital from 1 January 2014 onwards. For studies spanning periods before and after this date, we included those where the majority of the study period was after 1 January 2014. We searched databases on 26 April 2023 for papers published up to this date; the analysis took place from 2023 to 2025.

### 2.2 Data Analysis

We extracted data on year of study, world region, study design, age group, setting (intensive care unit [ICU] vs. ward), risk of death and causes of death. Year of study was defined by the mid‐point of data collection for the study. For cause of death, we grouped potential causes according to a modified version of the International Classification of Diseases ‐ 10 (ICD‐10) [10]. For “AIDS‐related death,” while our preferred definition was the USA Centre for Disease Control (CDC) “C” criteria [11], we accepted study authors’ alternative definitions when specified. Following CDC‐C criteria and in line with the previous 2015 review, “AIDS” included all forms of tuberculosis (whether pulmonary or extra‐pulmonary) but did not include bacterial infections (bacterial infections meet CDC‐C criteria only if “multiple or recurrent,” but it was not possible to disaggregate these from other bacterial infections). We preferentially extracted a single main cause of death per deceased person; however, when this was not possible because studies only reported multiple causes per person, we included all documented causes. For risk of death, we extracted number of in‐hospital deaths and number of people admitted. For studies where in‐hospital death was not reported, but deaths were measured over another period (e.g. 28 days from admission), we extracted deaths for any time period up to 6 months from admission. Where one or more reports presented data from the same group of participants (e.g. a primary paper and secondary analysis of the same dataset), we combined all available reports to extract data once for that cohort. Data extraction was performed using Google Sheets by one author (one of JE, NS, DSL, AR, RMB), with frequent discussion between all extractors to ensure consistency. All authors extracting data were physicians with experience in caring for hospitalized PLHIV, and all final extractions underwent verification by RMB.

We assessed risk of bias using a modified Newcastle Ottawa tool [12]. We considered the following key bias‐introducing domains: Representativeness of the cohort—specifically cohorts that recruited only a sub‐set of participants (e.g. only those newly diagnosed with HIV), and cohorts where some participants were recruited before first January 2014; Completeness of follow up—whether limited diagnostics were used (e.g. studies that only looked for tuberculosis) and whether limited diagnoses were reported (e.g. HIV‐associated vs. non‐HIV associated). Additionally, we noted concern if a study was an abstract rather than a full paper. Studies were assessed as having a higher risk of bias if one or more of these concerns were present.

We categorized studies into mutually exclusive categories of: “adults in medical wards” in seven world regions (Africa [AFR], USA and Canada [AMR‐N], America other than USA and Canada [AMR‐S], Eastern Mediterranean Region [EMR], Europe [EUR], South East Asia Region [SEAR] and Western Pacific Region [WPR]); “adults in ICU”; and “children.” For studies with both adults and children, or where age was not stated, these were grouped with adult studies since the number of children was typically small.

The denominator for risk of death was hospital admission (as many papers did not account for the possibility of multiple admissions per person). For overall risk of death from this review (i.e. including studies from 2014 to 2023), we constructed binomial Bayesian multi‐level regression models, with patient category and study as random effects. Estimates for the grand mean (i.e. overall risk of death) and estimates by category were obtained by taking 6000 draws from the posterior using the marginaleffects package [13], and summarized using the mean and quantile‐based 95% credible intervals (95% CrI).

For cause of death meta‐analyses, each cause of death was considered separately. Individual studies contributed to all the cause of death analyses for which they contained data, including studies that reported zero deaths for the cause of interest. If a study did not mention presence or absence of a cause of death, then it was excluded from the analysis of that cause. To estimate distributions of proportions of death in each disease category with 95% credible intervals [CrI], we fitted a binomial model with number of deaths from cause of interest and total number of deaths in included studies as an intercept‐only model with random effects terms for category and study. We used weakly informative priors to improve sampling (see Supplementary Material). We also report two pre‐specified restricted analyses: autopsy studies and studies at lower risk of bias.

For analysis of change in incidence of death over time, we obtained data from the published appendix of an earlier systematic review from 2015 [5] and verified with original papers. We combined data on risk of death from the 2015 and 2023 reviews and constructed a second Bayesian model with intercept and mid‐year of study (year since 2000) as fixed effects and category and study as random effects terms for both intercept and study mid‐year effect. Using posterior draws, we estimated the average marginal effect of one additional year at each category level (i.e. the slope) and the absolute and relative percentage differences between 2000 and 2023 by category.

For all models, we used the R brms package [14] to interface with Stan to run models. Model fitting was checked by *R̂* statistics, effective sample size measures, trace plots of chains and posterior predictive plots. Further detail on all models is in the Supplementary Material.

### 2.3 Role of the Funding Source

This work was funded through a grant from the Bill & Melinda Gates Foundation to the World Health Organization. The funder had no role in the design, data collection, analysis or interpretation of the data.

Ethical approval was not required as all data is aggregate data from previously published sources. Consent was not required as there were no data gathered on individuals.

## 3 Results

**FIGURE 1:**
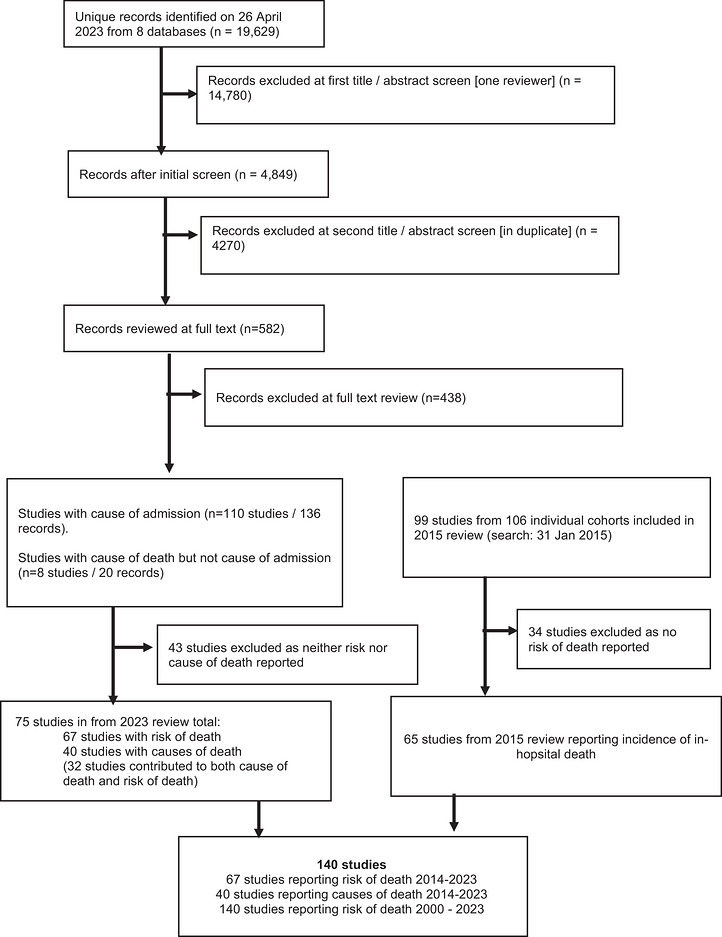
Modified PRISMA flow chart.

We identified 19,629 unique records, which yielded 75 eligible studies. Of these, 67 studies reported risk of death (2013−2023), 40 studies cause of death and 32 studies reported both (Figure 1 and Table S1). We also incorporated data on risk of death from 65 additional studies identified in the 2015 review.

Of the 75 included studies, sample sizes ranged from 20 to 26,021 admissions, with numbers of deaths ranging from 0 to 6,444. There were no studies from the Eastern Mediterranean Region and only one small study from the USA and Canada [15]. Among the 40 studies reporting cause of death, there were 9,955 deaths with 6,838 identifiable causes of death. Six studies included autopsy data: three cohorts from the CadMIA project (37 adults in Brazil, 73 adults in Mozambique, 18 children in Mozambique) [16], one from the TB Fast Track trial (34 adults in South Africa) [17], one study of 16 children in South Africa [18] and one study (48 adults and children) from Tanzania, which reported on tuberculosis prevalence at autopsy only [19]. One autopsy study was a sub‐study of a medical‐records study [20], the larger parent study is included in the meta‐analyses and the autopsy study only included in specific sub‐analyses. The non‐autopsy studies generally did not provide detailed information about diagnostics or how diagnoses were determined (Table S9). Nine cause of death studies, accounting for 922 causes of death, were assessed to be lower risk of bias [18, 21, 22, 23, 24, 25, 26, 27, 28].

Among the 12 studies reporting ART use [16, 17, 22, 24, 25, 26, 29, 30, 31, 32, 33, 34], 51% of people who died (285/556) were taking ART at hospital admission (range: 29%–83%). Of the six studies (378 people) reporting CD4 cell count among those who died [15, 17, 22, 24, 33, 35], the median was 48 cells/mm^3^ (range of medians: 34–109 cells/mm^3^). Only one study reported viral load data among people who died: 13/82 (16%) people with a measured viral load who died had an undetectable viral load [24]. Median CD4 count and viral load suppression among all admitted people are included in Table S2 [9].

### 3.1 Risk of Death Between 2014 and 2023

Sixty‐seven studies reported on risk of death for a total of 59,013 admissions among PLHIV, with an overall pooled risk of death of 16% (95% CrI: 8%–27%). Risk of death was highest among adults in ICUs (44%, 95% CrI 34%–54%), followed by adults in medical wards in Africa (19%, 95% CrI 15%–24%) and South East Asia (18%, 95% CrI 10%–31%) (Table 1). Substantial heterogeneity was observed between individual studies (Figure S1). Analysis restricted to the 60 studies measuring in‐hospital death, rather than death over a longer time period (potentially including post‐admission time), showed a similar risk of death (16%, 95% CrI 7%–27%, Table S3).

**TABLE 1:** Risk of death among hospitalized people living with HIV (2014−2023).

Patient category	Number of studies reporting risk of death	Number of deaths/number of admissions (%)	Pooled risk of death (95% CrI)
AFR: adults	20	8259/34,973 (24%)	19% (15%–24%)
AMR‐N: adults	1	9/93 (10%)	13% (4%–29%)
AMR‐S: adults	10	326/2894 (11%)	13% (9%–18%)
EUR: adults	14	275/3461 (8%)	8% (5%–11%)
SEAR: adults	3	121/556 (22%)	18% (10%–31%)
WPR: adults	2	1196/13,323 (9%)	11% (5%–20%)
Global: adults in ICU	11	1724/3233 (53%)	44% (34%–54%)
Global: children	6	73/480 (15%)	13% (7%–21%)
Overall	67	11,983/59,013 (20%)	16% (8%–27%)

*Note*: Pooled proportion of deaths is calculated using meta‐analysis with an intercept‐only Bayesian model with a random effect for each category level and study, as described in Methods.

Abbreviations: AFR, Africa region; AMR‐N, USA and Canada; AMR‐S, Americas other than USA and Canada; EUR, Europe region; ICU, intensive care unit; SEAR, South East Asia Region; WPR, Western Pacific Region; 95% Crl, 95% credible interval (based on percentile interval).

### 3.2 Causes of Death Between 2013 and 2023

**FIGURE 2:**
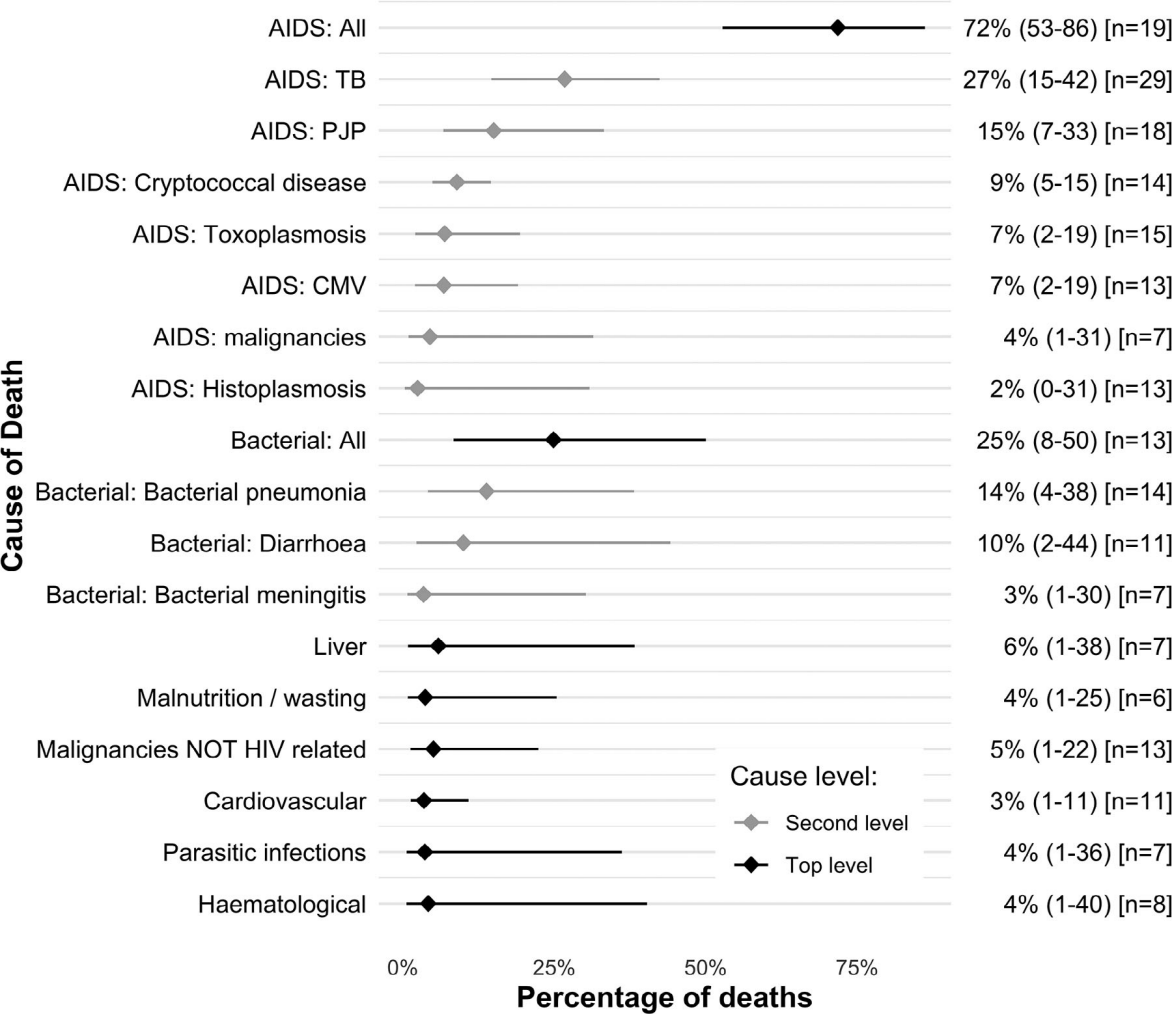
Meta‐analysis of causes of death among people living with HIV admitted to hospital. *Note*: Square brackets in the right column indicate number of studies that contributed data. Error bars represent 95% credible interval of the meta‐analysis estimate. Abbreviations: AIDS, acquired immunodeficiency syndrome; CMV, cytomegalovirus; PJP, *Pneumocystis jirovecii* pneumonia; TB, tuberculosis.

Forty studies reported 6,839 causes of death among 9,921 people who died. Among hospitalized PLHIV who died, AIDS‐related conditions were the predominant cause of death (72% of all deaths, 95% CrI 57%–85%), followed by bacterial infections (25%, 95% CrI 9%–47%) (Figure 2, Figure S2 and Table S4). Within AIDS‐related deaths, tuberculosis was the leading attributed cause (27% of all deaths, 95% CrI 15%–40%), followed by *Pneumocystis jirovecii* pneumonia (14%, 95% CrI 7%–29%) and cryptococcal disease (9%, 95% CrI 6%–13%). Bacterial infections (which were a separate category to AIDS) were the second commonest cause of death (25% of all deaths, 95% CrI 9%–47%).

**FIGURE 3:**
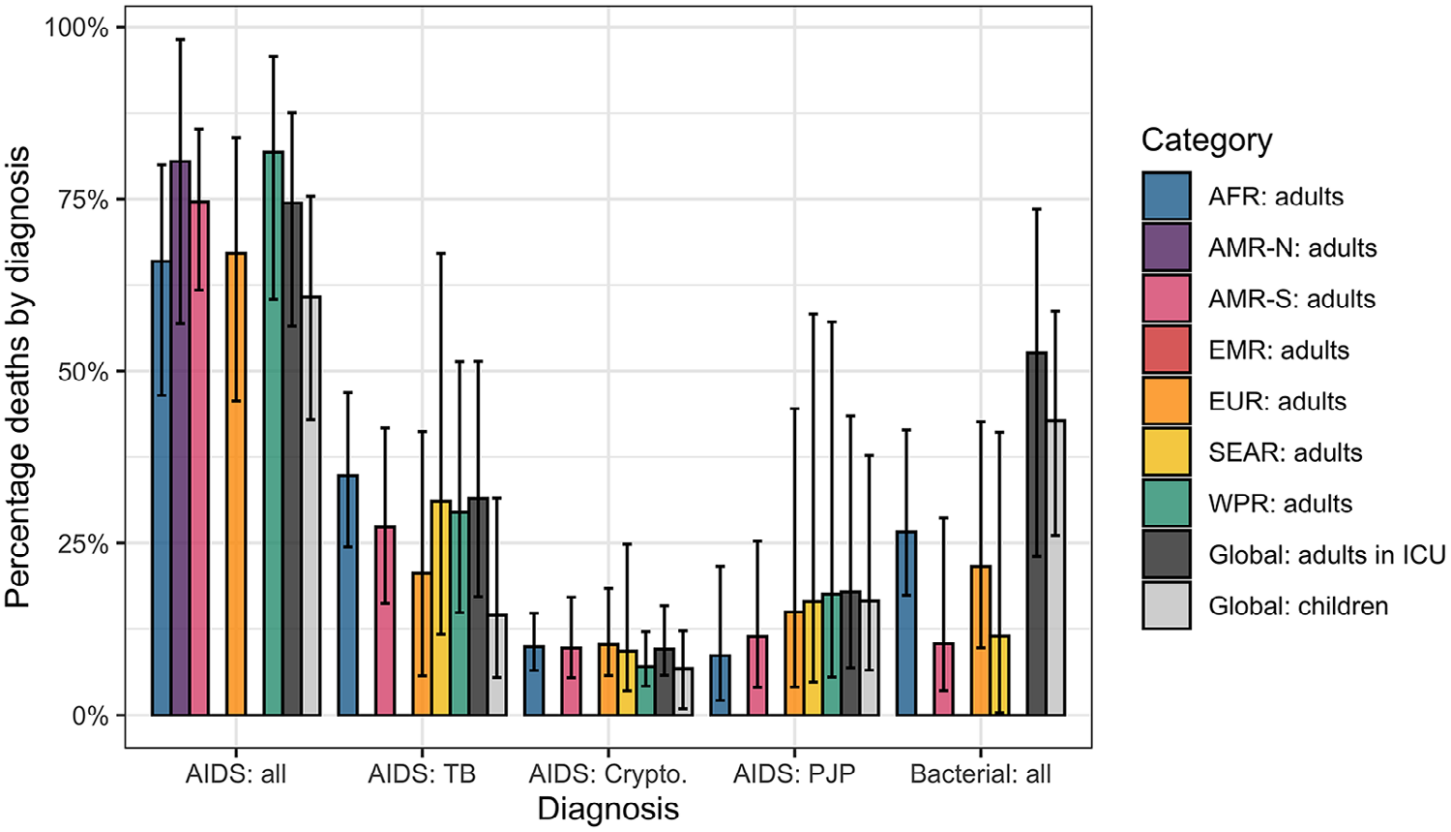
Pooled proportion of causes of death by category. Note there are missing estimates in some regions (shown as blank spaces), as there were no studies with information on some causes of death in some regions, and in two instances, cryptococcal deaths in children and bacterial infection in South East Asia, the point estimate is zero, and only the confidence interval is shown. Abbreviations: AFR, Africa region; AIDS, acquired immunodeficiency syndrome; AMR‐N, USA and Canada; AMR‐S, America other than USA and Canada; Crypto, cryptococcal meningitis; EMR, Eastern Mediterranean; EUR, Europe; ICU, intensive care unit; PJP, *Pneumocystis jirovecii* pneumonia; SEAR, South East Asia region; TB, tuberculosis; WPR, Western Pacific region.

There was substantial regional variation in causes of death, although analysis was complicated by limited regional data availability (Figures S7−S24). AIDS caused ≥50% adult deaths in all WHO regions with available data. Within the AIDS category, tuberculosis was the leading cause of death in adults in Africa (34%, 95% CrI: 24%–46%, 11 studies) [16, 21, 22, 23, 24, 36, 37, 38, 39], the Western Pacific Region (29%, 95% CrI 15%–50%, two studies), both from China [30, 40] and the Americas, excluding the USA and Canada (27%, 95% CrI 17%–41%, six studies) [16, 25, 26, 41, 42, 43]. Data on tuberculosis were limited in other regions, with single studies in Georgia [27] (12/109 deaths) and Myanmar [31] (3/6 deaths), and no data from the USA/Canada. Tuberculosis as a cause of death was reported less frequently in children (all five studies in children were in Africa; 15%, 95% CrI: 6%–31%) [16, 18, 28, 44, 45]. Cryptococcal deaths were common in adults in all regions, with point estimates ranging between 7% of deaths in the Western Pacific (based on two studies in China) [30, 40] to 10% in Africa (four studies) [16, 23, 24, 37]. Histoplasmosis as a cause of death was reported exclusively in South and Central America, causing 13% of deaths (95% CrI, 6%–22%, four studies) [16, 25, 43, 46]. Bacterial infections were particularly common as a cause of death in children (42% in children [95% CrI: 26%–57%], four studies) [16, 18, 28, 45] (Figure 3, Figure S3 and Table S5).

In autopsy studies, AIDS‐related conditions were identified in 68% of adult deaths (97/144 deaths in three studies [16, 17], 95% CrI: 54%–80%). Among autopsy studies of children, AIDS‐related conditions caused fewer deaths: 9/18 deaths in Mozambique [16] and 6/16 deaths in South Africa [18] (31% of child deaths, 95% CrI: 21%–51%). Tuberculosis caused 30% of adult deaths [16, 17, 19] (57/192 deaths in four autopsy studies, 95% CrI: 22%–39%), but was rare in children, causing 1/16 child deaths in a South African autopsy study [18] (3%, 95% CrI: 0.4%–18%) and 0/18 children deaths in a Mozambican study [16] (Figure S2). Bacterial infections caused 27% adult deaths (95% CrI 31%–64%) and 47% of child deaths (95% CrI: 31%–64%) in autopsy studies. See Figure S4 and Table S6.

Analysis restricted to the nine lower‐risk‐of bias studies which reported at least one cause of death [18, 21, 22, 23, 24, 25, 26, 27, 28] yielded broadly similar results: AIDS‐related conditions remained the predominant cause (65% of all adult deaths, 95% CrI 51%–76%, two studies), with tuberculosis deaths specifically being the most common AIDS‐related cause (32% of all adult deaths [95%CrI: 17–53], seven studies)—see Figure S5.

Where reported, non‐communicable diseases were an uncommon cause of death. Autopsy data from the CaDMIA studies [16] found only seven of 76 deaths among PLHIV from non‐infectious, non‐AIDS causes (two strokes, one each of acute myocardial infarction, dilated cardiomyopathy and acute myeloid leukaemia).

### 3.3 Change in Risk of Death 2000−2023

**FIGURE 4:**
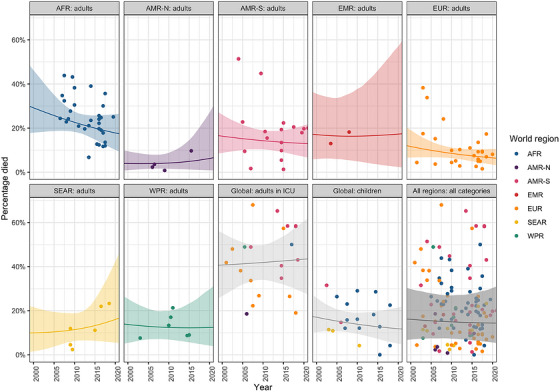
Risk of in‐hospital death among people living with HIV between 2000 and 2023. *Note*: Dots show study‐level empirical estimate of risk of death, and mid‐year of study with colour representing region of study. Lines show fitted regression line for effect of changing year, and shaded area represents 95% credible interval (percentile interval). Abbreviations: AFR, Africa region; AMR‐N, USA and Canada; AMR‐S, America other than USA and Canada; EUR, Europe; ICU, intensive care unit; SEAR, South East Asia region; WPR, Western Pacific region.

We found no strong evidence of change in risk of death among hospitalized PLHIV between 2000 and 2023 across any patient category. Globally, the point estimate for annual change in risk of death over 23 years from 2000 to 2023 was −2.2 percentage points, with 95% credible interval ranging from −16.1 to +17.0 percentage points. In most regions and subgroups, analyses suggested no change in mortality rates over time (i.e. the 95% credible interval for change was centred around zero). For adults in medical wards in Africa, there was very weak evidence for a possible decrease in risk of inpatient mortality (estimated annual change over 23 years: −13.6 percentage points, 95% CrI −41.7 to +6.6 percentage points), though we cannot exclude the possibility of no change or a small increase in deaths (Figure S6 and Tables S7 and S8). There were substantial differences in risk of death among different studies, even within regions and groupings (Figure 4).

## 4 Discussion

In this systematic review and meta‐analysis, we found that approximately one in six hospitalized PLHIV died, with substantial regional variability. AIDS‐related conditions, including tuberculosis, were the most common cause of deaths—including in six autopsy cohorts [16, 17, 18, 19]. Non‐communicable diseases were an uncommon reported cause of death in the included studies, although, of course, chronic comorbidities may increase the chance of dying from a more proximal AIDS‐related condition. We found no clear evidence of overall change in risk of death among hospitalized PLHIV between 2000 and 2023, though data suggested a possible reduction in Africa (estimated 14% reduction, however, 95% credible intervals are wide and include the possibility of no change over time, or slight increase).

Together with our previous paper on causes of hospital admission [9], and the 2015 systematic review [5], these findings demonstrate that AIDS‐related conditions remain the leading cause of hospital admission and of in‐hospital death among PLHIV, despite advances in HIV care and an increase in access to ART in the past decade. While approximately half of those who died were taking ART at admission, median CD4 counts were low (48 cells/mm^3^), suggesting that continued efforts for early diagnosis, rapid ART initiation, interventions to support adherence to ART, identification of advanced HIV disease [47] and effective treatment of opportunistic infections are crucial. Unfortunately, only one study reported viral load among those who died.

The strengths of this review include a comprehensive search strategy, including regional databases, inclusion of autopsy studies and data extraction by experienced physicians who could appropriately categorize causes of death. Limitations include the relatively small number of eligible studies leading to sparse regional data and incomplete diagnostic ascertainment in many studies. While we used statistical methods to account for uncertainty from small study numbers, quantifying the impact of diagnostic imprecision remains challenging. Some regions contributed no or very little data: for example the USA and Canada contributed only one study with nine deaths [15]. There were also relatively few studies in children, which limits inference for this group.

We only included papers that reported causes of admission or death, and it is possible there may be additional papers that only reported risk of death (and not causes of admission), which would not have been captured by this review. Additionally, our analysis used pooled data rather than individual patient data, limiting our ability to separate individual outcomes by precise date of admission or factors such as individual (as opposed to cohort) CD4/viral load.

With the exception of autopsy studies, cause of death ascertainment was poorly described in most studies, suggesting many deaths were classified based on clinical diagnosis without confirmatory testing. A comparative study of autopsy versus clinical‐pathological pre‐mortem diagnosis [48] found higher tuberculosis prevalence in post‐mortem studies than clinical record review alone, suggesting underdiagnosis of tuberculosis in clinical care. Access to specific diagnostic tests may vary both between diseases (e.g. diagnostics for tuberculosis and cryptococcal meningitis may be more available than those for histoplasmosis or *Pneumocystic jirovecci* pneumonia) and between settings (e.g. whether clinicians are able to access advanced imaging or histopathology services). This may have introduced both imprecision and potential systematic bias. However, autopsy studies showed similar patterns to non‐autopsy studies in our analysis, strengthening our confidence in the findings.

Where reported, non‐communicable diseases were an uncommon cause of death. While this may reflect diagnostic limitations, complete diagnostic autopsy data from the CaDMIA studies [16] found only seven of 76 deaths among PLHIV from non‐infectious, non‐AIDS causes. Although not a limitation of CaDMIA (which did both complete and minimally invasive autopsies), it is important to note that minimally invasive autopsies have greater accuracy for detecting infectious and malignant causes of death compared to other conditions, so other minimally invasive autopsy studies might miss non‐infectious causes of death [16].

Our pooled mortality estimate of 16% mortality is derived from the available data from very heterogeneous settings, including a disproportionate number of studies in ICUs and research‐active care settings, which may not be representative of settings where inpatients with HIV are managed globally. Mortality varies considerably but is substantial in all settings, highlighting an unacceptably high risk of death among people with HIV admitted to hospitals (indeed, the crude mortality among participants included in this study was even higher, at 20%). Further, many additional deaths occur in the immediate post‐discharge period. A recent systematic review found 14% of people who were alive at hospital discharge died within weeks of discharge [4]. A study in South Africa reported that by 6 months post‐discharge, half of the people had been readmitted at least once, while only 18% (19/121) attended outpatient follow‐up at their clinic within 1 month, mainly because of access to care difficulties [49]. There are limited data on ART initiation in hospital, but studies suggest that a proportion of patients are discharged without starting ART [50, 51].

While hospital admission rates among PLHIV may be declining in some settings [52, 53], we found no clear evidence of improving in‐hospital survival over time in any region, though data suggested possible modest improvements in Africa. Individual centre studies showed mixed trends, with increasing AIDS‐related admissions in Madagascar (2010−2016) [32], although this study did not report death trends. A study in Brazil showed an increase in mortality for PLHIV admitted to hospital, 2014–2017 [41]. This systematic search was conducted in April 2023. Although we did not systematically search the literature after 2023, we note some more recent studies from high‐income countries which did show a falling ICU mortality [54, 55]. A very large study from the USA found that the inpatient mortality rate for adults with an opportunistic infection was 6%, with no change between 2011 and 2018 [56]. There are several more recent large studies from low‐ and middle‐income countries which continue to show an extremely high mortality in these settings, including in people who were ART experienced (13% 30‐day mortality in Mexico City [57], 26% in‐hospital mortality in Uganda [58], and 28% 90‐day mortality in people with HIV or another chronic condition in Tanzania and Malawi) [59].

## 5 Conclusions

The persistently high mortality of hospitalized PLHIV, predominantly from AIDS‐related conditions and bacterial infections, necessitates renewed efforts to prevent advanced HIV through early HIV diagnosis and ART initiation as well as strengthened efforts to retain people in care. Research in various world regions using high‐quality diagnostics for inpatients and autopsies for people who died, with standard case definitions and prospective recruitment, would be helpful to fully understand the causes of death. For individuals with advanced HIV at diagnosis (CD4 count <200 cells/mm^3^), timely interventions like tuberculosis and cryptococcal screening and prophylaxis might reduce the burden of serious illness and hospitalization [60]. For those who become ill, improved diagnostics and treatment approaches for opportunistic infections are essential, as is effective post‐discharge linkage to care. Most of the causes of death identified in this review occur from treatable infections, highlighting the need to optimize existing interventions, but also to ensure accessibility of treatment to people who need them through timely referral, drug availability, diagnostics and supportive care.

## Author Contributions

NF conceived the study. RMB, NF and PM designed the study. RMB and JF designed the search strategy. JF ran the search and de‐duplication of results. RMB, JE and NS reviewed articles for inclusion, including PM, NF, RHB and JNJ as needed. RMB, JE, NS, AR, DSL and GB extracted data from the included papers. RMB, HR and PM provided input to the statistical methods. RMB conducted the statistical analysis, with assistance from PM. RMB wrote the first draft, and all authors contributed to revisions and approved the final manuscript. RMB and JE accessed and verified the data. All authors had full access to the data in the study and had final responsibility for the decision to submit for publication.

## Conflicts of Interest

RMB, DSL and JNJ all receive funding from the UK National Institute of Health Research to their institution. JNJ and DSL have also received funding from the US Centres for Disease Control to their institution. DSL has received salary support from Janssen to his institution. JNJ has served on DSMB for three trials related to hospitalized people living with HIV (Harvest, ARTIST, ASTRO trials). PM is DSMB chair for a trial related to hospitalized people living with HIV (IMPROVE trial). All other authors declare no conflicts of interest.

## Supporting information



Supporting Information: jia270134‐Sup‐0001‐SuppMat.pdf

## Data Availability

Summary data extracted from primary papers and the full search strategy for all databases is available at LSHTM Data Compass at https://datacompass.lshtm.ac.uk/id/eprint/4347/.
