# Supplementary material for: Cause of Death and Risk of Death for People Living With HIV Admitted to Hospital: A Systematic Review and Meta‐Analysis
Source: J Int AIDS Soc. 2026 Jun 9;29(6):e70134. doi: 10.1002/jia2.70134 (PMC13247553; doi:10.1002/jia2.70134)
Supplement: Supplementary file 1 — Supporting Information: jia270134‐Sup‐0001‐SuppMat.pdf [file JIA2-29-e70134-s001.pdf]

Supplementary material to:

"Cause of death and risk of death for people living with HIV admitted to hospital: a systematic review and meta-analysis."

Contents:

Summary of papers included
S Table 1: Summary of included studies and search sources

Overall incidence of death
S Table 2: CD4 and ART use by category
S Table 3: Incidence of death by time measured over
S Figure 1: Forest plot showing incidence of death with details of value in each study

Causes of death, including sensitivity analyses
S Table 4: Causes of death table
S Figure 2: Posterior draws from models to estimate causes of death
S Figure 3: Summary of posterior probability per diagnosis, by category
S Table 5: Cause of death by subgroup
S figure 4: Autopsy studies only – graph of cause of death
S Table 6: Deaths by cause in autopsy studies only
S figure 5: Lower risk of bias studies only

Change in risk of death over time
S Figure 6A: Average slopes for effect of year by category
Figure 6B: Summary of difference in posterior draws for a one-unit change in "year"
S Table 7: Slopes for effect of change over time
S Table 8: Estimated differences in risk of death over time

Details of papers included
S table 9: Papers included in cause of death: methods of diagnosis, definitions of AIDS and risk of bias judgement

Model specifications

References

Forest plots of each cause of death
S Figure 7: Forest plot for estimate of proportion of deaths due to AIDS (all)
S Figure 8: Forest plot for estimate of proportion of deaths due to TB
S Figure 9: Forest plot for estimate of proportion of deaths due to cryptococcal disease
S Figure 10: Forest plot for estimate of proportion of deaths due to Pneumocystis jirovecci pneumonia (PJP)
S Figure 11: Forest plot for estimate of proportion of deaths due to toxoplasmosis
S Figure 12: Forest plot for estimate of proportion of deaths due to AIDS related malignancies
S Figure 13: Forest plot for estimate of proportion of deaths due to CMV disease
S Figure 14: Forest plot for estimate of proportion of deaths due to histoplasmosis
S Figure 15: Forest plot for estimate of proportion of deaths due to bacterial infections (all)
S Figure 16: Forest plot for estimate of proportion of deaths due to bacterial pneumonia
S Figure 17: Forest plot for estimate of proportion of deaths due to bacterial meningitis
S Figure 18: Forest plot for estimate of proportion of deaths due to bacterial diarrhoea
S Figure 19: Forest plot for estimate of proportion of deaths due to malnutrition / wasting
S Figure 20: Forest plot for estimate of proportion of deaths due to parasitic infections (all)
S Figure 21: Forest plot for estimate of proportion of deaths due to malignancies, other than AIDS related malignancies
S Figure 22: Forest plot for estimate of proportion of deaths due to cardiovascular disease (all)
S Figure 23: Forest plot for estimate of proportion of deaths due to haematological disease (all)
S Figure 24: Forest plot for estimate of proportion of deaths due to Liver Disease (all)

PRISMA checklist

List of papers reviewed at full text and not included
S Table 10: Records considered at full text and reasons for exclusion

Note:
Further details including definitions of causes and protocol for systematic review (and search strategies) are included in a previous publication: *Burke RM, Sabet N, Ellis J, et al. Causes of hospitalisation among people living with HIV worldwide, 2014-23: a systematic review and meta-analysis. Lancet HIV. 2025;12(5):e355-e366. doi:10.1016/S2352-3018(24)00347-3*

S Table 1 : Summary of included studies and search sources

PAPER	Category	Region	Years recruitment	Source	Contributes to which analysis
Adlakha et. al. (1)	Global: adults in ICU	EUR	2006-2009	From 2015 review	Incidence death over time
Agaba et. al. (2)	AFR: adults	AFR	2007	From 2015 review	Incidence death over time
Akgun et. al. (3)	Global: adults in ICU	AMR-N	2002-2010	From 2015 review	Incidence death over time
Akinkuotu et. al. (4)	AFR: adults	AFR	2008-2009	From 2015 review	Incidence death over time
Albus et. al. (5)	AFR: adults	AFR	2017-2018	2023 cause of admission review	Cause & incidence of death
Alvarez et. al. (6)	AMR-S: adults	AMR-S	2014-2015	2023 cause of admission review	Cause & incidence of death
Andrade et. al. (7)	Global: adults in ICU	AMR-S	2014-2015	2023 cause of admission review	Cause & incidence of death
Andreu-Crespo et. al. (8)	EUR: adults	EUR	2016-2017	2023 cause of admission review	Cause & incidence of death
Apetse et. al. (9)	AFR: adults	AFR	2008	From 2015 review	Incidence death over time
Asensi-Diez et. al. (10)	EUR: adults	EUR	2016-2017	2023 cause of admission review	Cause & incidence of death
Aye et. al. (11)	SEAR: adults	SEAR	2018-2019	2023 cause of admission review	Incidence of death
Bachhuber et. al. (12)	AMR-N: adults	AMR-N	2009	From 2015 review	Incidence death over time
Balkhair et. al. (13)	EMR: adults	EMR	1999-2008	From 2015 review	Incidence death over time
Balogou et. al. (14)	Global: children	AFR	2010	From 2015 review	Incidence death over time
Barak et. al. (15)	AFR: adults	AFR	2015-2017	2023 cause of admission review	Cause & incidence of death
Barbier et. al. (16)	Global: adults in ICU	EUR	2008-2010	From 2015 review	Incidence death over time
Barrow et. al. (17)	AMR-S: adults	AMR-S	2007	From 2015 review	Incidence death over time
Bentley et. al. (18)	EUR: adults	EUR	2016	2023 cause of admission review	Incidence of death
Boniatti et. al. (19)	Global: adults in ICU	AMR-S	2012-2015	2023 cause of admission review	Cause & incidence of death
Borges et. al. (20)	AMR-S: adults	AMR-S	2021	2023 cause of admission review	Incidence of death
CaDMIA Brazil et. al. (21)	AMR-S: adults	AMR-S	2013-2015	New this review	Cause of death
CaDMIA Moz. adults et. al. (21)	AFR: adults	AFR	2013-2015	New this review	Cause of death
CaDMIA Moz. children et. al. (21)	Global: children	AFR	2013-2015	New this review	Cause of death
Caro-Vega et. al. (22)	AMR-S: adults	AMR-S	2020	2023 cause of admission review	Cause & incidence of death
Chawana et. al. (23)	Global: children	AFR	2015-2016	New this review	Cause of death
Chiang et. al. (24)	Global: adults in ICU	WPR	2001-2010	From 2015 review	Incidence death over time
Collins et. al. (25)	Global: children	SEAR	1999-2009	From 2015 review	Incidence death over time
Cordova et. al. (26)	AMR-S: adults	AMR-S	2004-2007	From 2015 review	Incidence death over time
Costales et. al. (27)	AFR: adults	AFR	2016-2019	New this review	Cause of death
Cunha et. al. (28)	Global: adults in ICU	AMR-S	2017-2019	2023 cause of admission review	Incidence of death
Dai et. al. (29)	WPR: adults	WPR	2009-2011	From 2015 review	Incidence death over time
Damasceno et. al. (30)	Global: adults in ICU	AMR-S	2018-2019	2023 cause of admission review	Incidence of death
De Oliveira et. al. (31)	AMR-S: adults	AMR-S	2011	From 2015 review	Incidence death over time
Dias et. al. (32)	EUR: adults	EUR	2005-2007	From 2015 review	Incidence death over time
Dicko et. al. (33)	Global: children	AFR	2010	From 2015 review	Incidence death over time
Dillon et. al. (34)	EUR: adults	EUR	2015-2020	2023 cause of admission review	Incidence of death
Dramowski et. al. (35)	Global: children	AFR	2007	From 2015 review	Incidence death over time
Elkhatiali et. al. (36)	Global: children	AFR	2017-2019	2023 cause of admission review	Cause & incidence of death
Falster et. al. (37)	WPR: adults	WPR	1999-2007	From 2015 review	Incidence death over time
Ferrand et. al. (38)	Global: children	AFR	2007-2008	From 2015 review	Incidence death over time

Fortes et. al. (39)	AFR: adults	AFR	2007-2008	From 2015 review	Incidence death over time
Frigati et. al. (40)	Global: children	AFR	2013-2018	2023 cause of admission review	Incidence of death
Galliet et. al. (41)	Global: adults in ICU	EUR	2016-2020	2023 cause of admission review	Incidence of death
Gama et. al. (42)	AMR-S: adults	AMR-S	2017-2018	2023 cause of admission review	Cause & incidence of death
Gel et. al. (43)	Global: children	AFR	2018	2023 cause of admission review	Incidence of death
Gonzalez-Fernandez et. al. (44)	AMR-S: adults	AMR-S	2013-2017	2023 cause of admission review	Incidence of death
Guedes et. al. (45)	AMR-S: adults	AMR-S	2014-2015	2023 cause of admission review	Cause & incidence of death
Guerro et. al. (46)	AMR-S: adults	AMR-S	2007-2012	From 2015 review	Incidence death over time
Guillen et. al. (47)	AMR-S: adults	AMR-S	2017	New this review	Cause of death
Gupta-Wright et. al. (48)	AFR: adults	AFR	2015-2017	2023 cause of admission review	Cause & incidence of death
Haachambwa et. al. (49)	AFR: adults	AFR	2017-2018	2023 cause of admission review	Incidence of death
Hajiabdolbaghi et. al. (50)	EMR: adults	EMR	2007-2009	From 2015 review	Incidence death over time
Hoffmann et. al. (51)	AFR: adults	AFR	2016	2023 cause of admission review	Incidence of death
Huerga et. al. (52)	AFR: adults	AFR	2015-2017	2023 cause of admission review	Incidence of death
Japiassu et. al. (53)	Global: adults in ICU	AMR-S	2006-2008	From 2015 review	Incidence death over time
Johnson et. al. (54)	EUR: adults	EUR	2016-2019	2023 cause of admission review	Incidence of death
Juniper et. al. (55)	EUR: adults	EUR	2018-2019	2023 cause of admission review	Incidence of death
Kang et. al. (56)	SEAR: adults	SEAR	2006-2013	From 2015 review	Incidence death over time
Kanyama et. al. (57)	AFR: adults	AFR	2016-2017	2023 cause of admission review	Cause & incidence of death
Kazibwe et. al. (58)	AFR: adults	AFR	2014-2019	2023 cause of admission review	Cause & incidence of death
Kendig et. al. (59)	AFR: adults	AFR	2012-2013	From 2015 review	Incidence death over time
Kim et. al. (60)	AMR-N: adults	AMR-N	2004-2008	From 2015 review	Incidence death over time
Kozhevnikova et. al. (61)	Global: adults in ICU	EUR	2014-2016	2023 cause of admission review	Incidence of death
Kra et. al. (62)	AFR: adults	AFR	2006-2007	From 2015 review	Incidence death over time
Krutikov et. al. (63)	EUR: adults	EUR	2016-2017	2023 cause of admission review	Cause & incidence of death
Kwara et. al. (64)	Global: children	AFR	2007-2008	From 2015 review	Incidence death over time
Laher et. al. (65)	AFR: adults	AFR	2017-2018	2023 cause of admission review	Incidence of death
Lakoh et. al. (66)	AFR: adults	AFR	2017	2023 cause of admission review	Cause & incidence of death
Lara-Medrano et. al. (67)	AMR-S: adults	AMR-S	2013-2017	2023 cause of admission review	Incidence of death
Laso et. al. (68)	EUR: adults	EUR	2014-2015	2023 cause of admission review	Cause & incidence of death
Leonard et. al. (69)	AMR-N: adults	AMR-N	2014-2017	2023 cause of admission review	Cause & incidence of death
Lewden et. al. (70)	AFR: adults	AFR	2010	From 2015 review	Incidence death over time
Li et. al. (71)	WPR: adults	WPR	2010-2019	2023 cause of admission review	Cause & incidence of death
Lima et. al. (72)	Global: adults in ICU	AMR-S	2015-2017	2023 cause of admission review	Incidence of death
Lucero et. al. (73)	EUR: adults	EUR	1996-2007	From 2015 review	Incidence death over time
Luz Brazil et. al. (74)	AMR-S: adults	AMR-S	2000-2008	From 2015 review	Incidence death over time
Luz France et. al. (74)	EUR: adults	EUR	2000-2008	From 2015 review	Incidence death over time
Maheswaran et. al. (75)	AFR: adults	AFR	2014	2023 cause of admission review	Incidence of death
Maphula et. al. (76)	Global: adults in ICU	AFR	2017	2023 cause of admission review	Incidence of death
Masoza et. al. (77)	Global: children	AFR	2014-2015	2023 cause of admission review	Cause & incidence of death
Matin et. al. (78)	SEAR: adults	SEAR	2008-2010	From 2015 review	Incidence death over time
Maxwell et. al. (79)	EUR: adults	EUR	2017-2018	2023 cause of admission review	Incidence of death
Medrano et. al. (80)	Global: adults in ICU	EUR	2005-2010	From 2015 review	Incidence death over time

Meng et. al. (81)	WPR: adults	WPR	2011-2019	2023 cause of admission review	Cause & incidence of death
Metallidis (Older cohort) et. al. (82)	EUR: adults	EUR	1998-2008	From 2015 review	Incidence death over time
Metallidis (Younger cohort) et. al. (82)	EUR: adults	EUR	1998-2008	From 2015 review	Incidence death over time
Meyers et. al. (83)	Global: children	AFR	2010-2011	From 2015 review	Incidence death over time
Miranda et. al. (84)	EUR: adults	EUR	2009-2011	From 2015 review	Incidence death over time
Mishore et. al. (85)	AFR: adults	AFR	2017	2023 cause of admission review	Incidence of death
Moreira et. al. (86)	Global: children	AMR-S	2001-2011	From 2015 review	Incidence death over time
Morquin et. al. (87)	Global: adults in ICU	EUR	1997-2008	From 2015 review	Incidence death over time
Mulu et. al. (88)	AFR: adults	AFR	2013-2014	2023 cause of admission review	Incidence of death
Naidoo et. al. (89)	AFR: adults	AFR	2015-2016	2023 cause of admission review	Cause & incidence of death
Namutebi et. al. (90)	AFR: adults	AFR	2011	From 2015 review	Incidence death over time
Nascimento et. al. (91)	AMR-S: adults	AMR-S	2012-2017	New this review	Cause & incidence of death
Neto et. al. (92)	Global: adults in ICU	AMR-S	2014-2015	2023 cause of admission review	Incidence of death
Njuguna et. al. (93)	Global: children	AFR	2013-2015	2023 cause of admission review	Cause & incidence of death
Nunez-Fernandez et. al. (94)	EUR: adults	EUR	2005-2007	From 2015 review	Incidence death over time
Nyandiko et. al. (95)	Global: children	AFR	2002-2008	From 2015 review	Incidence death over time
Ogoina et. al. (96)	AFR: adults	AFR	2006-2009	From 2015 review	Incidence death over time
Ondounda et. al. (97)	AFR: adults	AFR	2008-2010	From 2015 review	Incidence death over time
Ousley et. al. (98)	AFR: adults	AFR	2015-2017	2023 cause of admission review	Cause & incidence of death
Pandharpurkar et. al. (99)	SEAR: adults	SEAR	2016-2017	2023 cause of admission review	Incidence of death
Parry et. al. (100)	EUR: adults	EUR	2018-2019	2023 cause of admission review	Incidence of death
Paudel et. al. (101)	SEAR: adults	SEAR	2009	From 2015 review	Incidence death over time
Raga Almudever et. al. (102)	EUR: adults	EUR	2016-2018	2023 cause of admission review	Incidence of death
Rapp et. al. (103)	EUR: adults	EUR	2011	From 2015 review	Incidence death over time
Raubenheimer et. al. (104)	AFR: adults	AFR	2013-2014	2023 cause of admission review	Incidence of death
Ribeiro et. al. (105)	AMR-S: adults	AMR-S	2000-2010	From 2015 review	Incidence death over time
Ruiz et. al. (106)	Global: adults in ICU	AMR-S	2017-2019	2023 cause of admission review	Cause & incidence of death
Rukhadze et. al. (107)	EUR: adults	EUR	2014-2017	2023 cause of admission review	Cause & incidence of death
Saldarriaga-Arenas et. al. (108)	AMR-S: adults	AMR-S	2010-2011	From 2015 review	Incidence death over time
Santos et. al. (109)	Global: adults in ICU	EUR	1991-2011	From 2015 review	Incidence death over time
Schlabe et. al. (110)	Global: adults in ICU	EUR	2014-2019	2023 cause of admission review	Cause & incidence of death
Schlabe et. al. (110)	EUR: adults	EUR	2018-2022	2023 cause of admission review	Incidence of death
Senoglu et. al. (111)	EUR: adults	EUR	2015-2018	2023 cause of admission review	Incidence of death
Shahrin et. al. (112)	Global: children	SEAR	2009-2012	From 2015 review	Incidence death over time
Shrosbree et. al. (113)	Global: adults in ICU	EUR	2000-2009	From 2015 review	Incidence death over time
Sture et. al. (114)	EUR: adults	EUR	2009-2011	From 2015 review	Incidence death over time
Sudjaritruk et. al. (115)	Global: children	SEAR	2003	From 2015 review	Incidence death over time
TB Fast Track Beckwith (116)	AFR: adults	AFR	2013-2015	2023 cause of admission review	Cause & incidence of death
TB Fast Track Karat (117)	AFR: adults	AFR	2013-2015	New this review	Cause of death
Tepungipame et. al. (118)	AFR: adults	AFR	2019-2020	2023 cause of admission review	Cause & incidence of death
Thinyane et. al. (119)	AFR: adults	AFR	2010	From 2015 review	Incidence death over time
Thit et. al. (120)	SEAR: adults	SEAR	2015	2023 cause of admission review	Cause & incidence of death
Thompson et. al. (121)	AMR-N: adults	AMR-N	2003-2010	From 2015 review	Incidence death over time

Tittle Italy et. al. (122)	EUR: adults	EUR	2012	From 2015 review	Incidence death over time
Tittle UK et. al. (122)	EUR: adults	EUR	2012	From 2015 review	Incidence death over time
Traore et. al. (123)	AFR: adults	AFR	2009-2010	From 2015 review	Incidence death over time
Umeta et. al. (124)	AFR: adults	AFR	2017	2023 cause of admission review	Incidence of death
Vega Carlos et. al. (125)	AMR-S: adults	AMR-S	2018	New this review	Cause of death
Viana et. al. (126)	Global: children	AMR-S	1998-2007	From 2015 review	Incidence death over time
Vidal et. al. (127)	AMR-S: adults	AMR-S	2019-2020	2023 cause of admission review	Cause & incidence of death
Vidal et. al. (127)	AMR-S: adults	AMR-S	2014-2015	2023 cause of admission review	Incidence of death
Vidal-Cortes et. al. (128)	Global: adults in ICU	EUR	2005-2009	From 2015 review	Incidence death over time
Wen et. al. (129)	WPR: adults	WPR	2010-2012	From 2015 review	Incidence death over time
Whitehorn et. al. (130)	EUR: adults	EUR	2007-2009	From 2015 review	Incidence death over time
Xiao et. al. (131)	WPR: adults	WPR	2009-2012	From 2015 review	Incidence death over time
du Ploy et. al. (132)	Global: children	AFR	2015-2016	2023 cause of admission review	Incidence of death
van Schalkwyk et. al. (133)	AFR: adults	AFR	2015-2016	2023 cause of admission review	Incidence of death

S Table 2: CD4 and ART use by category

Median CD4 count and reported ART use at admission in studies included in this review, by category. Many studies did not report ART use or CD4 counts.

A more detailed overview of all CD4, ART use and viral loads among all hospital cohorts with cause of admission (including those that did not report risk of death or cause of death) is available at previous paper (Burke et. al, TLHIV, 2025)

Category	ART use at admission	Median CD4 (IQR)
AFR: adults	6024/8780 (69%), 17 studies	177 (81-232), 12 studies
AMR-S: adults	1112/1747 (64%), 6 studies	70 (31-174), 6 studies
EUR: adults	554/1351 (41%), 3 studies	120 (112-200), 7 studies
SEAR: adults	153/370 (41%), 1 study	104 (101-108), 2 studies
WPR: adults	308/711 (43%), 1 study	0 studies
Global: adults in ICU	892/2865 (31%), 8 studies	137 (82-148), 5 studies
Global: children	20/79 (25%), 2 studies	0 studies

S table 3: Incidence of death by time measured over

	Using broad criteria (including studies where deaths measured over a variety of times)			Using strict criteria (in hospital death only)		
Category	Number studies	Number deaths / Number admissions (crude %)	Percentage deaths by meta-analysis (95% CI)	Number studies	Number deaths / Number admissions (crude %)	Risk of death by meta-analysis (95% CrI)
AFR: adults	20	8259/34973 (24%)	19% (15-24%)	18	7721/32278 (24%)	19% (14-24%)
AMR-N: adults	1	9/93 (10%)	13% (4-28%)	1	9/93 (10%)	13% (3-26%)
AMR-S: adults	10	326/2894 (11%)	13% (9-18%)	10	326/2894 (11%)	13% (8-18%)
EUR: adults	14	275/3461 (8%)	8% (5-11%)	12	165/1940 (9%)	8% (5-11%)
Global: adults in ICU	11	1724/3233 (53%)	44% (34-55%)	9	1672/3068 (54%)	47% (36-58%)
Global: children	6	73/480 (15%)	13% (7-21%)	5	40/299 (13%)	12% (6-19%)
SEAR: adults	3	121/556 (22%)	18% (10-30%)	3	121/556 (22%)	19% (9-30%)
WPR: adults	2	1196/13323 (9%)	11% (5-20%)	2	1196/13323 (9%)	11% (4-19%)
**Overall**	**67**	11983/59013 (20%)	16% (8-27%)	60	11250/54451 (21%)	16% (7-27%)

S table 2 note: "Strict" criteria exclude seven studies where death measured over a different time period to in-hospital deaths only. These seven studies are: Njuguna et. al. (deaths measured 28 days from admission); Rukhadze et. al (deaths measured 2 weeks from discharge); Gupta-Wright et. al (deaths measure 56 days from admission); Hoffman et. al. (deaths up to six months from discharge); Johnson et. al. (timing is unclear, one death occurred); Galliet et. al. (death within 60 days of ICU admission); Damasceno et. al. (death within 28 days from admission).

S Figure 1: Forest plot showing incidence of death with details of value in each study

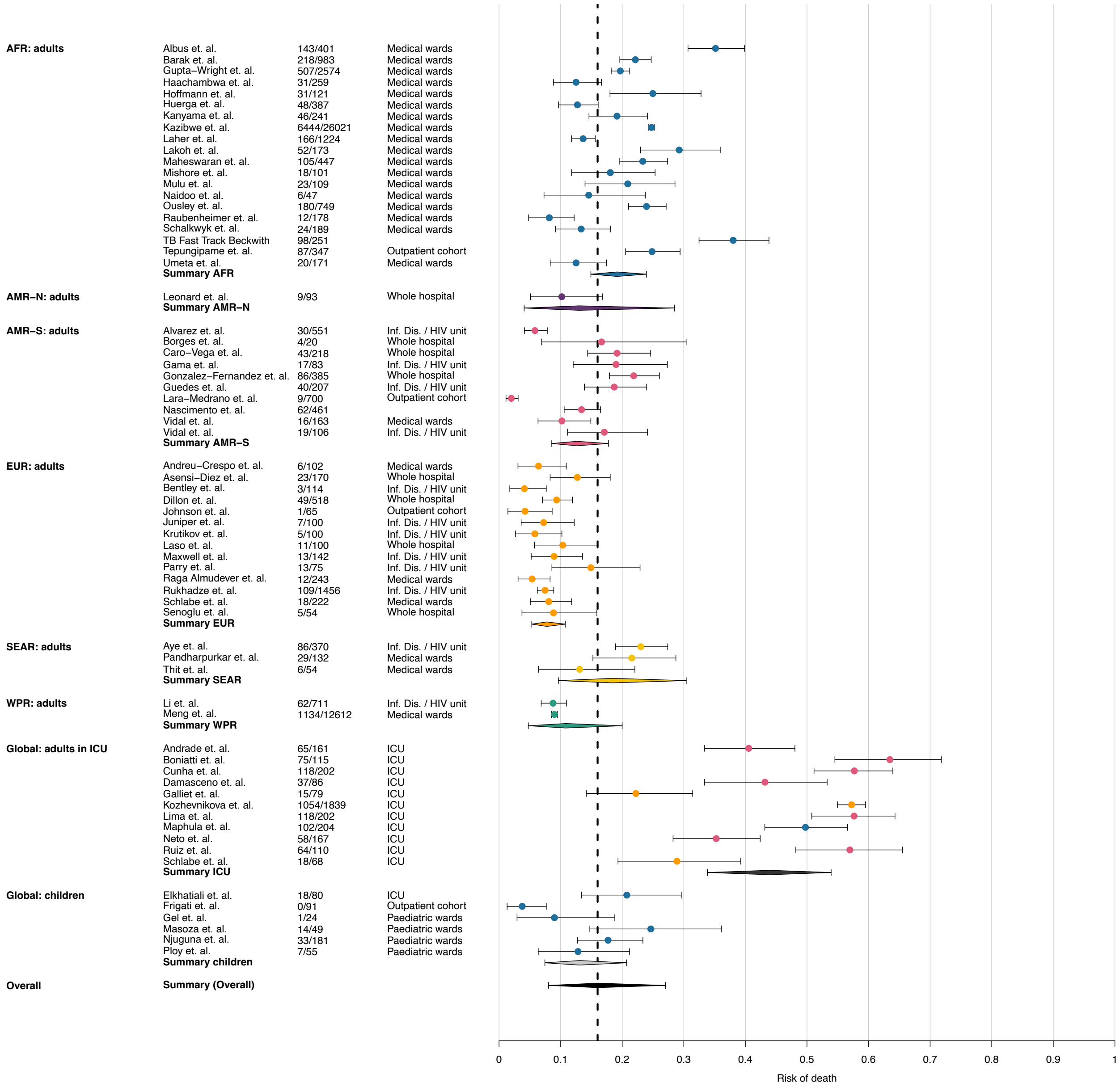

S table 4: Causes of death table

	Meta-analysis result [number of studies]	Number of people who died from cause / denominator (crude percentage deaths)
AIDS: All	72% (50 - 93) [n=19]	556/796 (70%)
AIDS: TB	28% (8 - 50) [n=29]	3241/9741 (33%)
AIDS: PJP	14% (4 – 39)[n=18]	162/899 (18%)
AIDS: Cryptococcal disease	9% (4 - 16) [n=14]	147/1924 (8%)
AIDS: Toxoplasmosis	8% (1 - 18) [n=15]	78/838 (9%)
AIDS: CMV	7% (1 - 23) [n=13]	150/1686 (9%)
AIDS: malignancies	4% (1 - 16) [n=7]	9/223 (4%)
AIDS: Histoplasmosis	1% (0 - 17) [n=13]	23/554 (4%)
Bacterial: All	26% (4 - 63) [n=13]	156/540 (29%)
Bacterial: Bacterial pneumonia	13% (3 - 44) [n=14]	777/1888 (41%)
Bacterial: Diarrhoea	9% (2 - 27) [n=11]	57/688 (8%)
Bacterial: Bacterial meningitis	3% (1 - 9) [n=7]	5/202 (2%)
Liver	5% (0 - 30) [n=7]	25/277 (9%)
Malnutrition / wasting	3% (0 - 12) [n=6]	9/266 (3%)
Malignancies NOT HIV related	4% (1 - 22) [n=13]	25/589 (4%)
Cardiovascular	3% (1 - 10) [n=11]	18/564 (3%)
Parasitic infections	3% (0-10) [n=7]	9/294 (3%)
Haematological	3% (0-18) [n=8]	47/663 (7%)

S table 4 note: If a study reported no deaths from a particular cause, zero deaths from that cause were recorded in the numerator, and the number of deaths in the denominator. Where a study did not mention a potential cause of death at all, those deaths were not included in the numerator or denominator. Accordingly, different causes of death have different numbers of studies contributing and different denominators.

Note that crude estimate for proportion of deaths from bacterial pneumonia is 41% (777 deaths / 1888 people in denominator, across 14 studies), whereas meta-regression estimate is 13% (95% CrI 3 – 44%). This is due to one very large study (Meng et. al.) with unusually high deaths from bacterial pneumonia (687 bacterial pneumonia deaths /1134 total deaths in that one study).

S Figure 2: Posterior distributions of causes of death

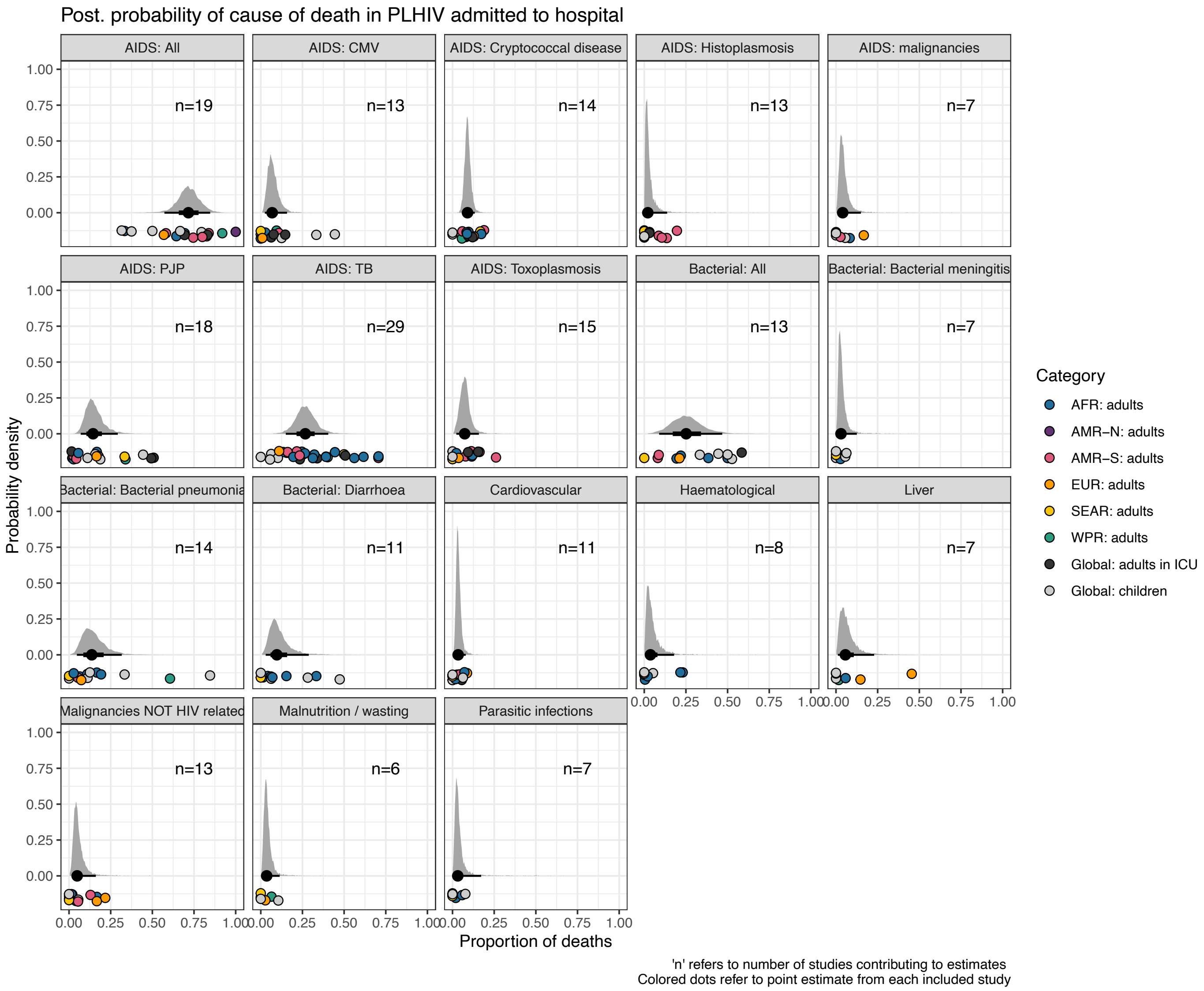

S Figure 2 note

Posterior probability density (summary of draws) from Bayesian models for proportion of deaths from each cause, point estimate and 95% credible interval (mean and quantiles) are summarised in black point and line at bottom of each posterior density plot. Each facet is a separate model.

Dots at the bottom of each plot indicate estimates of proportion of deaths for each contributing study, vertical "jitter" added to improve visualisation of points, but there remains some unavoidable overplotting on a graph this size / resolution.

S Figure 3: Summary of posterior probability per diagnosis, by category

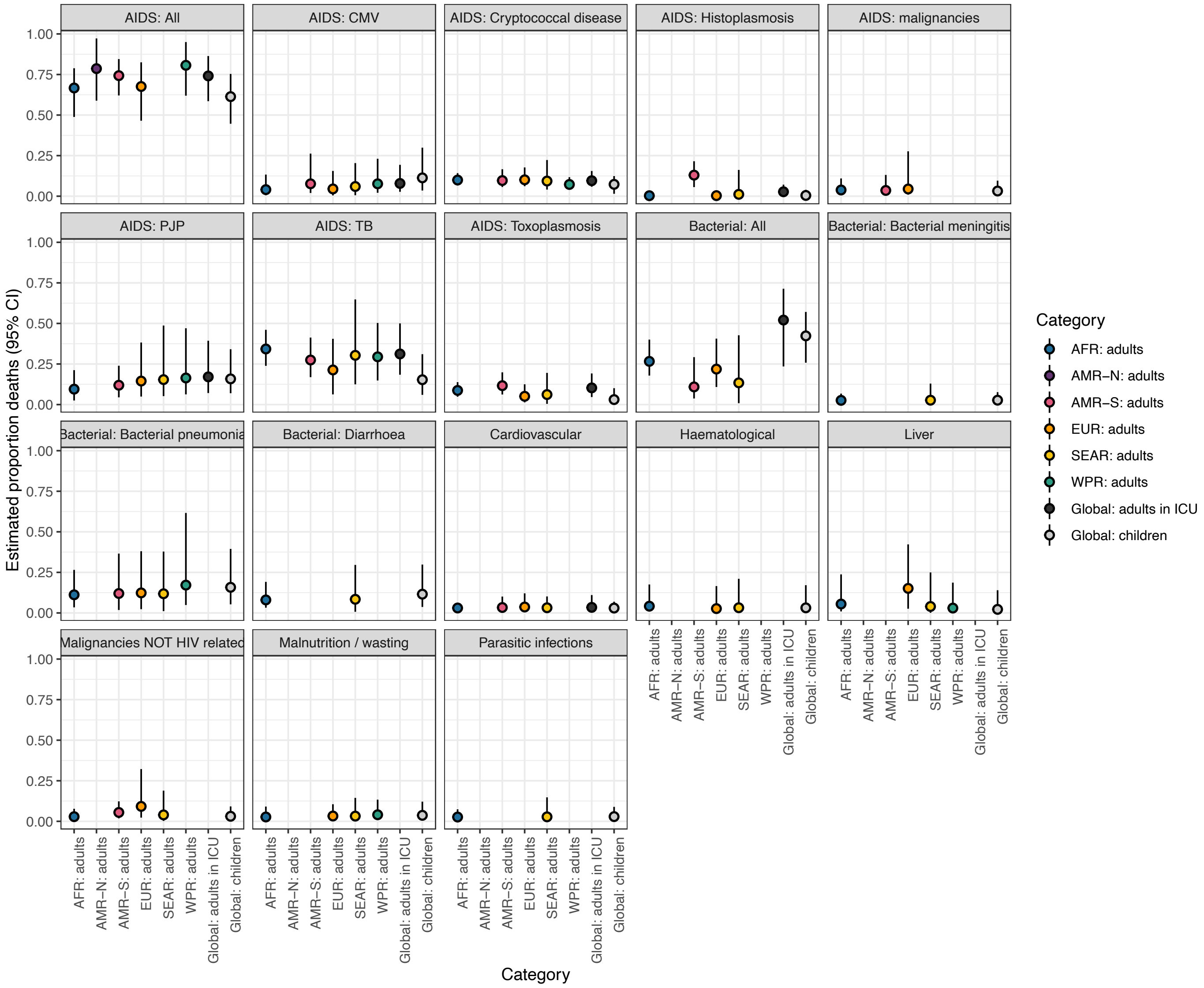

S Fig 3 notes:

Summary of median and 95% credible interval for posterior probability of deaths due to each cause (see facet panel label) as a proportion of all deaths which occurred. Only levels where at least one study (i.e. one estimate of proportion of deaths) was observed are included.

S Table 5: Cause of death by subgroup

Diagnosis	AFR: adults	AMR-N: adults	AMR-S: adults	EUR: adults	SEAR: adults	WPR: adults	Global: adults in ICU	Global: children	OVERALL
AIDS all	67% (49 - 79)	79% (59 - 97)	74% (62 - 84)	68% (47 - 82)	NA	81% (62 - 95)	74% (59 - 86)	61% (45 - 75)	72% (57 - 85)
AIDS TB	34% (24 - 46)	NA	27% (17 - 41)	21% (6 - 41)	30% (12 - 65)	29% (15 - 50)	31% (18 - 50)	15% (6 - 31)	27% (15 - 40)
AIDS Crypto	10% (7 - 14)	NA	10% (6 - 17)	10% (6 - 18)	9% (4 - 22)	7% (4 - 12)	10% (6 - 16)	7% (1 - 12)	9% (6 - 13)
AIDS PJP	9% (3 - 21)	NA	12% (4 - 24)	14% (5 - 38)	15% (5 - 49)	16% (6 - 47)	17% (7 - 39)	16% (7 - 34)	14% (7 - 29)
AIDS Toxo	9% (5 - 14)	NA	12% (6 - 20)	5% (1 - 13)	6% (0 - 20)	NA	10% (5 - 19)	3% (0 - 10)	7% (2 - 16)
AIDS malignancies	4% (1 - 11)	NA	4% (1 - 13)	4% (1 - 28)	NA	NA	NA	3% (0 - 10)	4% (1 - 15)
AIDS CMV disease	4% (1 - 13)	NA	8% (2 - 26)	4% (0 - 16)	6% (1 - 20)	8% (2 - 23)	8% (3 - 19)	11% (3 - 30)	7% (2 - 16)
AIDS histoplasmosis	0% (0 - 2)	NA	13% (6 - 22)	0% (0 - 3)	1% (0 - 16)	NA	3% (1 - 7)	0% (0 - 4)	2% (0 - 14)
Bacterial all	27% (18 - 40)	NA	11% (4 - 29)	22% (11 - 41)	13% (1 - 43)	NA	52% (23 - 71)	42% (26 - 57)	25% (9 - 47)
Bacterial Bacterial pneumonia	11% (3 - 27)	NA	12% (2 - 37)	12% (2 - 38)	12% (1 - 38)	17% (5 - 62)	NA	16% (5 - 39)	14% (5 - 32)
Bacterial Bacterial meningitis	3% (1 - 7)	NA	NA	NA	3% (0 - 13)	NA	NA	3% (1 - 8)	3% (1 - 13)
Bacterial Diaorrhea	8% (3 - 19)	NA	NA	NA	8% (1 - 30)	NA	NA	12% (4 - 30)	10% (3 - 29)
Malnutrition / wasting all	3% (0 - 9)	NA	NA	3% (1 - 11)	3% (0 - 14)	4% (1 - 13)	NA	4% (1 - 12)	4% (1 - 11)
Parasitic infections all	3% (1 - 7)	NA	NA	NA	3% (0 - 15)	NA	NA	3% (1 - 9)	3% (1 - 17)
Malignancies NOT HIV related all	3% (1 - 8)	NA	5% (2 - 12)	9% (2 - 32)	4% (0 - 19)	NA	NA	3% (1 - 9)	5% (2 - 16)
Cardiovascular all	3% (1 - 6)	NA	3% (1 - 10)	4% (1 - 12)	3% (1 - 10)	NA	3% (1 - 11)	3% (1 - 7)	3% (1 - 8)
Haematological all	4% (1 - 18)	NA	NA	3% (0 - 17)	3% (0 - 21)	NA	NA	3% (0 - 17)	4% (1 - 18)
Liver all	5% (1 - 24)	NA	NA	15% (3 - 42)	4% (0 - 25)	3% (0 - 19)	NA	2% (0 - 14)	6% (1 - 23)

S figure 4: Autopsy studies only

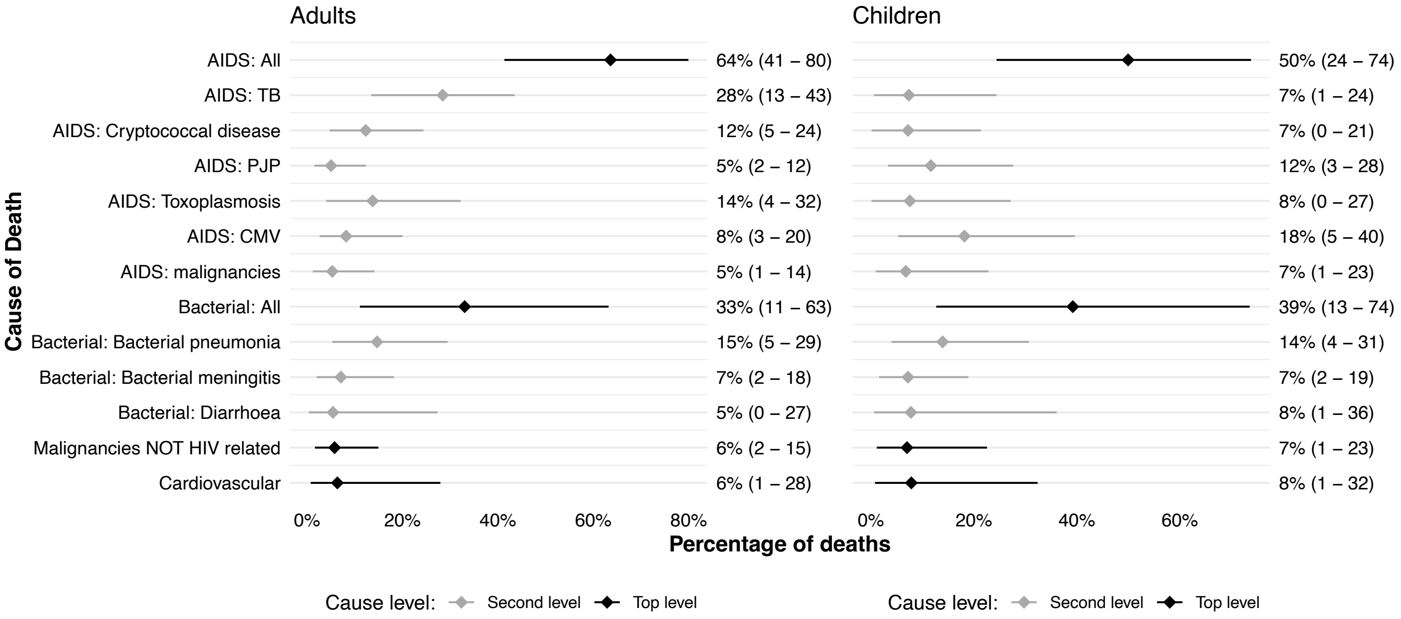

S figure 4 notes: Meta-analysis of causes of death at autopsy.

There are two studies in children (only) although not all causes were reported in both. Children studies from Mozambique (complete diagnostic autopsy, 18 children) and South Africa (minimally invasive tissue sampling, 16 children).

One study in Tanzania included both adults and children and only reported on TB presence or absence, there were 48 HIV+ive participants (included here). The entire study had 17% (36/205) children (HIV positive and negative) but didn't break down by age and HIV status. All participants in this study are included with adults for this meta-analysis – this is relevant for TB line only.

Three further studies in adults were included these are from Mozambique (complete diagnostic autopsy, 73 adults), Brazil (complete diagnostic autopsy, 37 adults) and South Africa (minimally invasive tissue sampling, 34 adults, 25 of whom were in hospital at the time of death). Numbers in square brackets indicate number of studies contributing to estimate.

S Table 6 : Deaths by cause in autopsy studies only

Cause	Adults		Children	
	Crude numbers deaths / denominator (%)	Meta-analysis proportion	Crude numbers deaths / denominator (%)	Meta-analysis proportion
AIDS: All	64% (41 - 80)	97/144 [n=3]	50% (24 - 74)	15/34 [n=2]
AIDS: TB	28% (13 - 43)	57/192 [n=4]	7% (1 - 24)	1/34 [n=2]
AIDS: Cryptococcal disease	12% (5 - 24)	18/144 [n=3]	7% (0 - 21)	0/18 [n=1]
AIDS: PJP	5% (2 - 12)	5/144 [n=3]	12% (3 - 28)	5/34 [n=2]
AIDS: Toxoplasmosis	14% (4 - 32)	15/110 [n=2]	8% (0 - 27)	0/18 [n=1]
AIDS: CMV	8% (3 - 20)	8/144 [n=3]	18% (5 - 40)	8/34 [n=2]
AIDS: malignancies	5% (1 - 14)	7/144 [n=3]	7% (1 - 23)	1/18 [n=1]
Bacterial: All	33% (11 - 63)	42/144 [n=3]	39% (13 - 74)	16/34 [n=2]
Bacterial: Bacterial pneumonia	15% (5 - 29)	21/144 [n=3]	14% (4 - 31)	4/34 [n=2]
Bacterial: Bacterial meningitis	7% (2 - 18)	6/107 [n=2]	7% (2 - 19)	2/34 [n=2]
Bacterial: Diarrhoea	5% (0 - 27)	1/73 [n=1]	8% (1 - 36)	1/16 [n=1]
Malignancies NOT HIV related	6% (2 - 15)	5/110 [n=2]	7% (1 - 23)	1/18 [n=1]
Cardiovascular all	2/73 (3%)	3% (1-10) [n=1]	1/16 (6%)	6% (1-34) [n=1]

S figure 5: Lower risk of bias studies

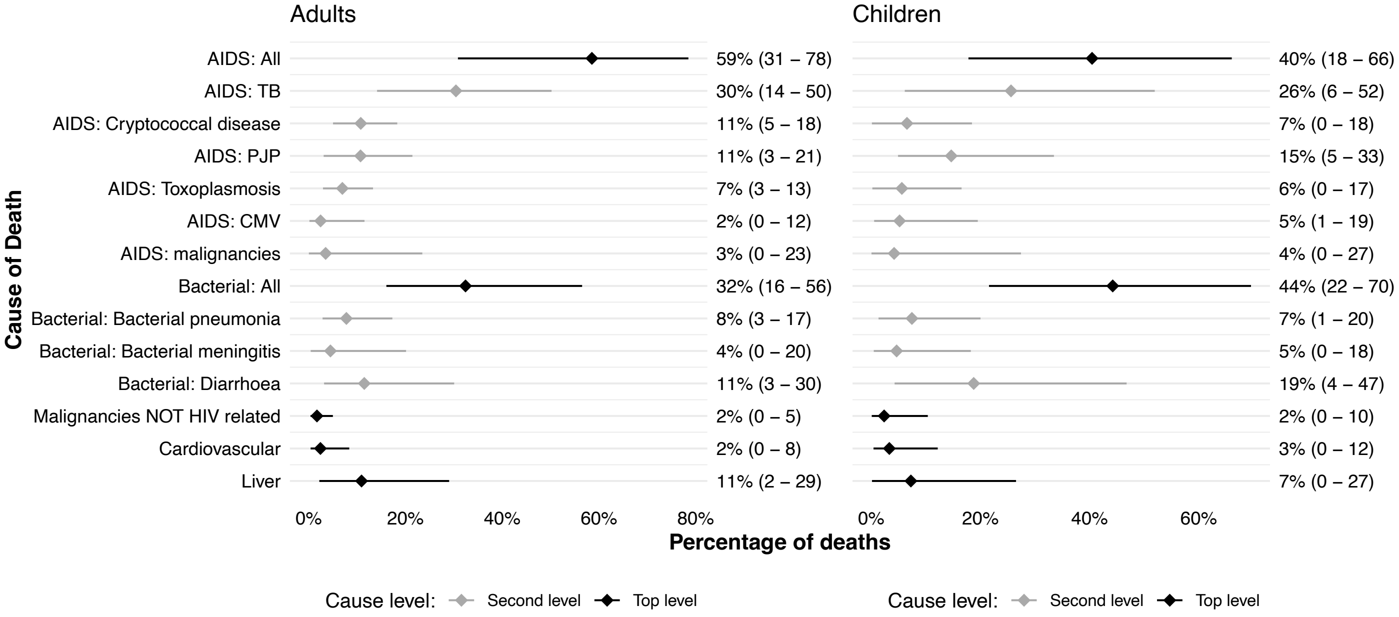

S figure 5 notes: Meta-analysis of causes of death in lower risk of bias studies.

Bias was assessed via a modified Newcastle Ottawa Scale. Representativeness of the cohort was assessed as whether a general group was recruited compared to more restrictive recruitment (e.g. based on CD4 criteria) and whether all participants were recruited post 1st Jan 2014 or whether the study spanned before and after this date. Assessment of outcome was based on whether all diagnoses were potentially included or just one, whether reporting of diagnoses was adequate and whether the study was a full paper vs. an abstract only. Studies were judged a lower risk of bias if none of the above issues were present, and at higher risk of bias if there were some concerns in one or more domain(s).

Nine studies were judged to have lower risk of bias. These include two studies in children (an autopsy study in South Africa and a medical records review study in Tanzania) and seven studies in adults (in Botswana, Sierra Leone, Democratic Republic of the Congo, Guinea, Venezula, Brazil and Georgia – all medical record review studies).

S Figure 6A: Average slopes for effect of year by category

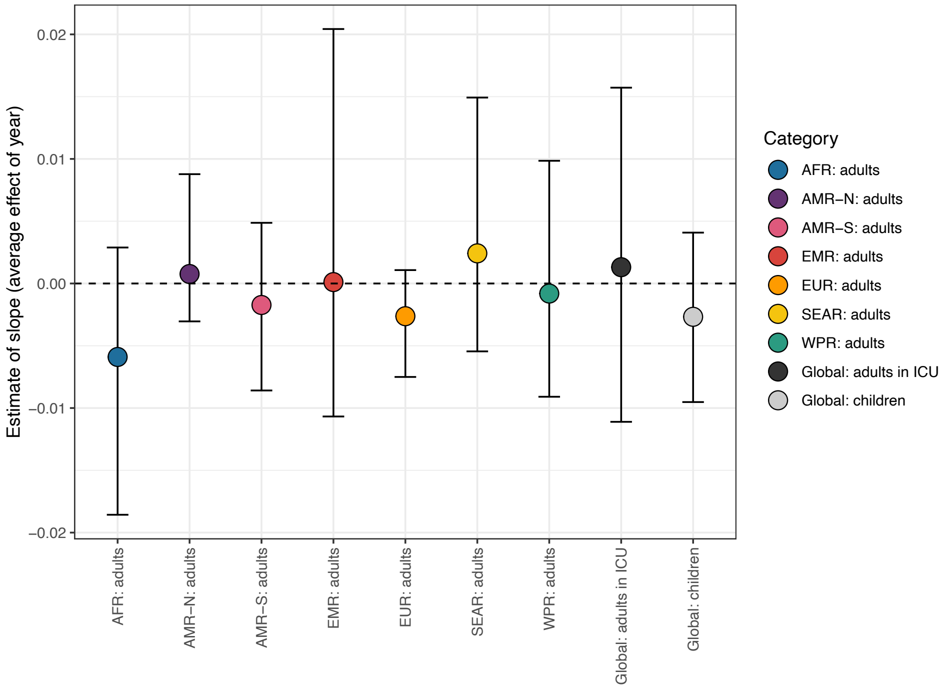

Dotted line is line of null effect (i.e. a slope of zero). Slopes are the average slope on the response scale for a change of 1 year, together with confidence intervals. All confidence intervals cross no effect. Calculated by marginaleffects package.

Figure 6B: Summary of difference in posterior draws for a one-unit change in "year"

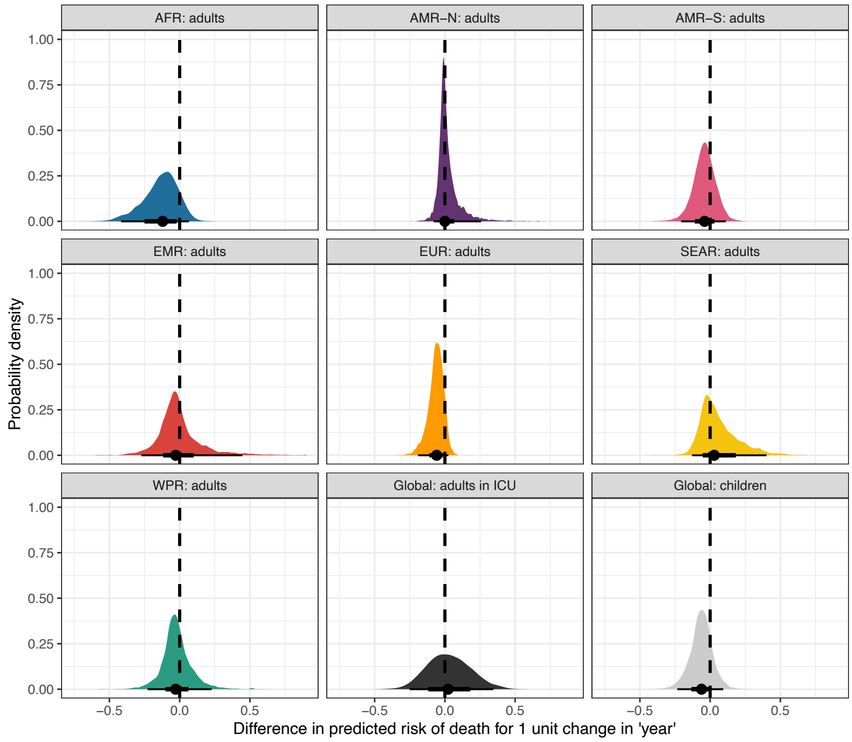

S Table 7: Slopes for effect of change over time

Category	Slope estimate	Lower bound 95% CrI	Upper bound 95% CrL
AFR: adults	-0.0059	-0.0186	0.00289
AMR-N: adults	0.000762	-0.00304	0.00878
AMR-S: adults	-0.00173	-0.00859	0.00487
EMR: adults	0.000112	-0.0107	0.0204
EUR: adults	-0.00263	-0.0075	0.00107
SEAR: adults	0.00242	-0.00545	0.0149
WPR: adults	-0.00081	-0.00909	0.00985
Global: adults in ICU	0.00131	-0.0111	0.0157
Global: children	-0.00267	-0.00952	0.00408

Average effect of one year change on number of people who died (i.e. slopes), expressed as proportions (i.e. on response scale)

S table 8: Estimated differences in risk of death over time

		**Estimated risk of death (proportion, 95% CrI)**		**Difference in risk of death from 2000 to 2023 (difference in proportion, 95% CrI)**
**Category**	**Number of studies (total)**	**Year = 2000**	**Year = 2023**	
AFR: adults	32	0.31 (0.17-0.53)	0.18 (0.1-0.26)	-0.136 (-0.417-0.066)
AMR-N: adults	4	0.04 (0.01-0.11)	0.06 (0.01-0.27)	0.022 (-0.08-0.258)
AMR-S: adults	17	0.17 (0.08-0.3)	0.13 (0.07-0.21)	-0.041 (-0.206-0.112)
EMR: adults	2	0.18 (0.05-0.39)	0.17 (0.02-0.57)	-0.004 (-0.272-0.446)
EUR: adults	26	0.13 (0.07-0.23)	0.06 (0.03-0.11)	-0.064 (-0.191-0.025)
SEAR: adults	6	0.1 (0.01-0.23)	0.16 (0.06-0.42)	0.062 (-0.129-0.401)
WPR: adults	6	0.14 (0.04-0.3)	0.12 (0.04-0.3)	-0.02 (-0.228-0.228)
Global: adults in ICU	21	0.4 (0.23-0.58)	0.43 (0.28-0.61)	0.029 (-0.251-0.345)
Global: children	18	0.18 (0.1-0.3)	0.12 (0.05-0.21)	-0.064 (-0.234-0.093)

Values and 95% credible interval (percentile intervals) from posterior estimates, for year 2000 (year since 2000 = 0) and year 2023 (year = 2023), together with summary of posterior distribution of difference between those years.

S table 9: Papers included in cause of death: methods of diagnosis, definitions of AIDS and risk of bias judgement

PAPER	Category	Country	Method diagnosis	Definition AIDS	Items related to risk of bias						Overall risk of bias
					Abstract only	Restrict population?	How restricted?	Spans 1st Jan 2014	Limited range diagnoses considered	Limited reporting	Overall risk of bias
Albus et. al.	AFR: adults	Guinea	Some information on available tests (e.g.malaria, and sputum Xpert tests), but not specific definitions for OI.	NA	Full paper	No	*NA*	No	No	No	Lower ROB
Alvarez et. al .	AMR-S: adults	Colombia	No information on how Ois diagnosed or available diagnostics in context	"AIDS definining" ("the definitions were based on previous studies ad those establised by National Institutes of Health")	Full paper	No	*NA*	No	No	Yes, only focuses one diagnosis	Higher ROB
Andrade et. al.	Global: adults in ICU	Brazil	Minimal information	AIDS as defined by CDC	Full paper	Yes	*NA*	No	No	No	Higher ROB
Andreu-Crespo et. al .	EUR: adults	Spain	No information on how Ois diagnosed or available diagnostics in context	Not defined	Abstract	No	*NA*	No	No	Yes, only focuses one diagnosis	Higher ROB
Asensi-Diez et. al.	EUR: adults	Spain	No information on how Ois diagnosed or available diagnostics in context; definitions based on ICD-10.	Doesnt mention. Uses ICD-10 but doesn't split of AIDS vs. other diagnoses	Full paper	No	*NA*	No	Yes	Yes, only focuses one diagnosis	Higher ROB
Barak et. al.	AFR: adults	Botswana	No specific information on diagnostics or definitions, based on medical record review.	NA	Full paper	No	*NA*	No	No	No	Lower ROB
Boniatti et. al.	Global: adults in ICU	Brazil	Includes clinical-laboratory case definitions for Ois.	Presumably CDC-C criteria	Full paper	Yes	ART naïve	Includes old (some pre-2014)	No	No	Higher ROB
Caro-Vega et. al.	AMR-S: adults	Mexico	No specific information on diagnostics or definitions, based on medical record review.	Not defined	Full paper	Yes	During 2020 COVID only	No	Yes	Yes, only focuses one diagnosis	Higher ROB
Chawana et. al.	Global: children	South Africa	MIA	CDC-C (every person has cause of death, so categorised for this review according to our definition)	Full paper	No	*NA*	No	No	No	Lower ROB
Elkhatiali et. al.	Global: children	South Africa	No information on how Ois diagnosed or available diagnostics in context	CDC-C (every person has cause of death, so categorised for this review according to our definition)	Full paper	Yes	ICU children under 2 years	No	No	No	Higher ROB
Gama et. al.	AMR-S: adults	Brazil	No information on how relevant Ois diagnosed or	NA	Full paper	No	*NA*	No	No	No	Lower ROB

			available diagnostics in context (does have information about some laboratory data and serology for some pathogens). No information about TB diagnostics or definitions.								
Guedes et. a l.	AMR-S: adults	Brazil	Study about visceral leishmaniasis only, detailed diagnostics provided.	NA	Full paper	No	*NA*	No	Yes	Yes, only focuses one diagnosis	Higher ROB
Guillen et. al .	AMR-S: adults	Venezuela	No specific information on diagnostics or definitions, based on medical record review.	CDC-C (every person has cause of death, so categorised for this review according to our definition)	Full paper	Yes	Only if full record found	No	No	No	Higher ROB
Gupta-Wright et. al .	AFR: adults	Malawi and South Africa	TB diagnosed based on sputum Xpert (provided to everyone), urine LAM and Xpert (half participants), chest Xray (if requested) and clinical decision making.	NA	Full paper	No	*NA*	No	Yes	Yes, only focuses one diagnosis	Higher ROB
Kanyama et. al.	AFR: adults	Malawi	Serum CrAg, urine LAM and urine Xpert offered to everyone in principle (although some people missed due to operational reasons / lack of test availability). Sputum Xpert also commonly requested by clinical teams. Radiology at clinical discretion.	NA	Full paper	No	*NA*	No	Yes	Yes, only focuses one diagnosis	Higher ROB
Kazibwe et. al.	AFR: adults	Uganda	Minimal information on how Ois diagnosed or available diagnosetics in context	NA	Full paper	No	*NA*	No	Yes	Yes, only focuses one diagnosis	Higher ROB
Krutikov et. al.	EUR: adults	UK	No specific information on diagnostics or definitions, based on medical record review.	Not stated	Abstract	No	*NA*	No	No	Yes	Higher ROB
Lakoh et. al.	AFR: adults	Sierra Leone	Clear clinical-laboratory definitions provided, no mandatory diagnostics provided.	CDC-C (every person has cause of death, so categorised for this review according to our definition)	Full paper	No	*NA*	No	No	No	Lower ROB
Laso et. al.	EUR: adults	Spain	No information on how Ois diagnosed or available diagnostics in context	NA	Abstract	No	*NA*	No	No	Yes, only focuses one diagnosis	Higher ROB
Leonard et. al.	AMR-N: adults	USA	No information on how Ois diagnosed or available diagnostics in context	"AIDS related complications"	Abstract	Yes	New HIV diagnoses	No	Yes	Yes, only focuses one diagnosis	Higher ROB

Li et. al.	WPR: adults	China	No information on how Ois diagnosed or available diagnostics in context	"AIDS related" (not otherwise defined)	Full paper	Yes	ID inpatients	Includes old (some pre-2014)	No	No	Higher ROB
Masoza et. al.	Global: children	Tanzania	No information on how Ois diagnosed or available diagnostics in context	CDC-C (every person has cause of death, so categorised for this review according to our definition)	Full paper	No	Children 1 month to 12 years	No	No	No	Lower ROB
Meng et. al.	WPR: adults	China	No specific information definitions, very limited information on diagnostics used, based on medical record review.	NA	Full paper	No	ID hospital	Includes old (some pre-2014)	No	No	Higher ROB
Naidoo et. al.	AFR: adults	South Africa	No specific information on diagnostics or definitions, based on medical record review.	WHO AIDS defining	Full paper	Yes	50 years +	No	No	No	Higher ROB
Nascimento et. al.	AMR-S: adults	Brazil	No definitions provided for causes	NA	Full paper	No	*NA*	Includes old (some pre-2014)	No	No	Higher ROB
Njuguna et. al.	Global: children	Kenya	No clear information on how Ois diagnosed or available diagnostics in context, based on information in a substudy there might have been systematic use of TB testing (LAM, gastric aspirates on Xpert)	WHO Stage III / stage IV	Full paper	Yes	Children all ART naive	Includes old (some pre-2014)	Yes	No	Higher ROB
Ousley et. al.	AFR: adults	Kenya and DRC	Some information on available tests (Xpert MTB/rif, LAM, CrAg, malaria RDTs), but not specific definitions for OI.	NA	Full paper	No	*NA*	No	No	Yes, only focuses one diagnosis	Higher ROB
Ruiz et. al.	Global: adults in ICU	Colombia		NA	Full paper	Yes	*NA*	No	No	No	Higher ROB
Rukhadze et. al.	EUR: adults	Georgia	No specific information on diagnostics or definitions, based on medical record review.	"AIDS-defining conditions included those from the clinical US CDC, as well as Hodgkin's lymphoma and non-Hodgkin's lymphoma of all cell types, and visceral leishmaniasis"	Full paper	No	ID ward	No	No	No	Lower ROB
Schlabe et. al.	Global: adults in ICU	Germany	No information on how Ois diagnosed or available diagnostics in context; does contain clear definitions for AIDS	NA	Full paper	Yes	*NA*	No	No	No	Higher ROB
Tepungipame et. al.	AFR: adults	DRC	No specific information on diagnostics or definitions,	AIDs related isn't clearly defined	Full paper	No	*NA*	No	No	No	Lower ROB

			based on medical record review.								
Thit et. al.	SEAR: adults	Myanmar	Detailed criteria provided, mostly clinical with supportive diagnostics. Xpert available for TB, but empiric clinically diagnoses also allowed	NA	Full paper	No	*NA*	No	Yes	No	Higher ROB
Vega Carlos et. al.	AMR-S: adults	Venezula	Some information about TB diagnostics (sputum Xpert), empiric TB diagnoses also included. Urine LAM tests done, but not included in TB definitions.	"Related to AIDS" (no definition given)	Full paper	No	*NA*	No	No	No	Lower ROB
Vidal et. al.	AMR-S: adults	Brazil	No information on how Ois diagnosed or available diagnostics in context	NA	Full paper	Yes	CD4 <200	No	Yes	Yes, only focuses one diagnosis	Higher ROB
CaDMIA Brazil et. al.	AMR-S: adults	Brazil	Testing using galactomannan and histoplasma PCR on people with CD4 <200	CDC-C (every person has cause of death, so categorised for this review according to our definition)	Full paper	No	*NA*	Includes old (some pre-2014)	No	No	Higher ROB
CaDMIA Moz. adults et. al.	AFR: adults	Mozambique	CDA	CDC-C (every person has cause of death, so categorised for this review according to our definition)	Full paper	No	*NA*	Includes old (some pre-2014)	No	No	Higher ROB
CaDMIA Moz. children et. al.	Global: children	Mozambique	CDA	CDC-C (every person has cause of death, so categorised for this review according to our definition)	Full paper	No	*NA*	Includes old (some pre-2014)	No	No	Higher ROB
Costales et. al.	AFR: adults	Tanzania	CDA	NA	Full paper	No	*NA*	No	Yes	Yes, only focuses one diagnosis	Higher ROB
TB Fast Track Beckwith	AFR: adults	South Africa	Mixture of CDA / MIA	"Admissions secondary to WHO clinical stage IV diagnoses" plus TB	Full paper	Yes	CD4 <150, ART naïve	Includes old (some pre-2014)	No	No	Higher ROB
TB Fast Track Karat	AFR: adults	South Africa	No standardised diagnostics, but clear information provided about how diagnosis arrived at after review of medical records.	NA	Full paper	Yes	CD4 <150, ART naïve	Includes old (some pre-2014)	No	No	Higher ROB

MIA = Minimally invasive autopsy, CDA = Complete diagnostic autopsy, CDC-C = Centres for disease control and prevention criteria for AIDS, list "C", ID inpatients = Inpatients in infectious disease ward, ROB = Risk of Bias, OI = Opportunistic infection.

Appendix: Model specifications

A: Model for pooled incidence of death (not including effect of year)

Model Specification – binomial model with intercepts only

$$n_{\{died,ij\}} \sim Binominal\left(n_{\{trials,ij\}}, p_{\{ij\}}\right\}$$
$$logit\left(p_{\{ij\}}\right) = \mu + u_j + v_{ij}$$
$$u_j \sim N(0, \sigma^2_{category})$$
$$v_{ij} \sim N(0, \sigma^2_{study})$$

Where:

$n_{\{died,ij\}}$: number of deaths in study *i* in category *j*
$n_{\{trials,ij\}}$: number of participants in study *i* in category *j* (i.e. the binomial denominator)
p_{ij} : probability that a participant in study *i* and category *j* dies
μ : overall intercept (grand mean on the logit scale)
u_j: random effect for category *j*
v_{ij}: random effect for study *i* within category *j*
$\sigma_{category}$: standard deviation of category-level random effects
σ_{study}: standard deviation of study-level random effects

Coded in "brms" as: **n_died | trials(n_people) ~ 1 + (1 | category/study).**

We used the default brms priors, specifically μ, $\sigma_{category}$ and σ_{study}were assigned a Student-t prior with 3 degrees of freedom, mean 0 and scale 2.5.

B: Model for death by cause

This is run as 18 separate models; one for each cause of death of interest.

Model Specification – binomial model with intercepts only

$$n_{\{cause,ij\}} \sim Binominal\left(n_{\{died,ij\}}, p_{\{ij\}}\right\}$$
$$logit\left(p_{\{ij\}}\right) = \mu + u_j + v_{ij}$$
$$u_j \sim N(0, \sigma^2_{category})$$
$$v_{ij} \sim N(0, \sigma^2_{category})$$

Where:

$n_{\{cause,ij\}}$: number of deaths from specified cause in study *i* in category *j*
$n_{\{died,ij\}}$: total number of deaths in study *i* in category *j* (i.e. the binomial denominator)
p_{ij} : probability that a death in study *i* and category *j* was due to the specified cause
μ : overall intercept (grand mean on the logit scale)
u_j: random effect for category *j*
v_{ij}: random effect for study *i* within category *j*
$\sigma_{category}$: standard deviation of category-level random effects
σ_{study}: standard deviation of study-level random effects

Coded in "brms" as: **n_cause | trials(n_died) ~ 1 | category/study**

Priors

This model (but not the others) includes weakly informative prior of the intercept, to enable models with relatively sparse data to run.

$$\mu \sim N(-1, 1.68^2)$$

This prior of a mean log-odds (logit scale) of 1.68 is equivalent to a probability about 27%, two standard deviations either side range from 1% probability to 91% probability.

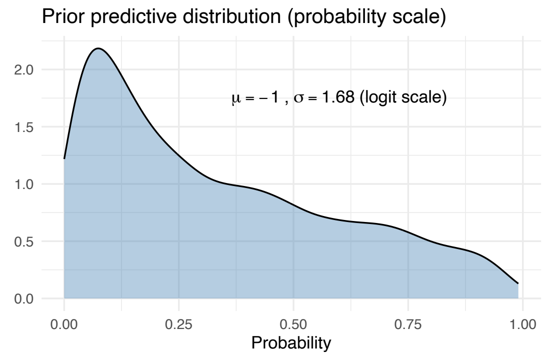

The remainder of the coefficients (other than μ were supplied according to the brms default priors – all Student-t prior with 3 degrees of freedom, mean 0 and scale 2.5.

C: Model for effect of year on incidence of death

Model Specification – binomial model with random slopes and random intercepts

$$n_{\{died_{ij}\}} \sim Binomial\ (n_{people_{ij}}, p_{\{ij\}})$$
$$logit(p_{\{ij\}}) = \mu + \beta_{year} * year_{\{ij\}} + u_{\{0j\}} + u_{\{1j\}} year_{\{ij\}} + v_{\{0ij\}} + v_{\{1j\}} year_{\{ij\}}$$
$$(u_{\{0j\}}, u_{\{1j\}})^T \sim N(0, \Sigma_u)^T$$
$$(v_{\{0ij\}}, v_{\{1ij\}})^T \sim N(0, \Sigma_v)^T$$

Where:

$n_{died\{ij\}}$: number of deaths observed in study *i* within category *j*
$n_{people\{ij\}}$: total number of people within study *i* within category *j*
$p_{\{ij\}}$: probability of death in study *i* within category *j*
$year_{\{ij\}}$: calendar year in of midpoint of study *i* within category *j* (in years since 1997)
μ: overall (grand mean) intercept on logit scale
β_{year}: fixed effect (population-level slope) of year
$u_{\{0j\}}, u_{\{1j\}}$: category level random intercept and random slope for year
$v_{\{0ij\}}, v_{\{1ij\}}$: study level random intercept for year (study *i* within category *j)*
Σ_u : 2x2 variance – covariance matrix of category level random effects
Σ_v: 2x2 variance – covariance matrix of category level random effects

Coded in "brms" as: **n_died | trials(n_people) ~ 1 + year + ((1 + year) | category/study)**

Fitting and summarising

All models were fitted in "brms" R package, as an interface to Stan. Each model was fun with six chains and 2000 iterations per chain (half of which were warm-up/burn in). Posterior predictions were generated using "tidybayes" R package, with 6000 draws for each model parameter set. All summary measures are based on mean of posterior draws and 95% credible interval based on quantiles. Where needed, slopes were calculated using the "marginaleffects" R package.

References

1. Adlakha A, Pavlou M, Walker DA, Copas AJ, Dufty N, Batson S et al. Survival of HIV-infected patients admitted to the intensive care unit in the era of highly active antiretroviral therapy. International Journal of STD and AIDS 2011;22:498–504.
2. Agaba PA, Digin E, Makai R, et al. Clinical characteristics and predictors of mortality in hospitalized HIV-infected Nigerians. Journal of Infection in Developing Countries 2011; 5(5): 377-82.
3. Akgun KM, Tate JP, Pisani M, et al. Medical ICU admission diagnoses and outcomes in human immunodeficiency virus-infected and virus-uninfected veterans in the combination antiretroviral era. Critical Care Medicine 2013; 41(6): 1458-67.
4. Akinkuotu A, Roemer E, Richardson A, et al. In-hospital mortality rates and HIV: a medical ward review, Lilongwe, Malawi. International Journal of STD & AIDS 2011; 22(8): 465-70.
5. Albus SL, Harrison RE, Moudachirou R, et al. Poor outcomes 35 among critically ill HIV-positive patients at hospital discharge and post-discharge in Guinea, Conakry: a retrospective cohort study. PLoS One 2023; 18: e0281425.
6. Álvarez Barreneche MF, Restrepo Castro CA, Hidrón Botero A, et al. Hospitalization causes and outcomes in HIV patients in the late antiretroviral era in Colombia. AIDS Res Ther 2017; 14: 60.
7. Andrade HB, da Silva I, Ramos GV, et al. Short- and medium-term prognosis of HIV-infected patients receiving intensive care: a Brazilian multicentre prospective cohort study. HIV Med 2020;
8. Andreu-Crespo A, Llibre JM, Cardona-Peitx G, et al. Hospital admissions due to medical conditions in a public health care system with free access to antiretroviral treatment. J Int AIDS Soc 2018; 21: 61–62.
9. Apetse K, Assogba K, Kevi K, Balogou AA, Pitche P, Grunitzky E. [Opportunistic infections of the HIV/AIDS in adults in hospital settings in Togo]. Bulletin de la Societe de Pathologie Exotique 2011; 104(5): 352-4.
10. Asensi-Diez R, Fernández-Cuerva C, Alcaraz Sánchez JJ, Muñoz-Castillo I. Diagnóstico al alta y causas de mortalidad de pacientes VIH+ ingresados en un hospital de tercer nivel. Rev Esp Quimioter 2019; 32: 317–26.
11. Aye, K.-P., Phyu, S., Mon, T.-S. & Soe, T.-Z. #21: Pattern and Outcome of Opportunistic Infections in Hospitalized HIV- Infected Patients in Specialist Hospital Waibargi, Myanmar. Journal of the Pediatric Infectious Diseases Society 10, S19–S19 (2021).
12. Bachhuber MA, Southern WN. Hospitalization rates of people living with HIV in the United States, 2009. Public Health Reports 2014; 129(2): 178-86.
13. Balkhair AA, Al-Muharrmi ZK, Ganguly S, Al-Jabri AA. Spectrum of AIDS Defining Opportunistic Infections in a Series of 77 Hospitalised HIV-infected Omani Patients. Sultan Qaboos University Medical Journal 2012; 12(4): 442-8.
14. Balogou AA, Saka B, Kombate D, et al. Causes of mortality associated with HIV/AIDS in health-care facilities in Togo: a six-month prospective study. Tropical Doctor 2011; 41(4): 215-7.
15. Barak T, Neo DT, Tapela N, et al. HIV-associated morbidity and mortality in a setting of high ART coverage: prospective surveillance results from a district hospital in Botswana. J Int AIDS Soc 2019;
16. Barbier F, Roux A, Canet E, et al. Temporal trends in critical events complicating HIV infection: 1999-2010 multicentre cohort study in France. Intensive Care Medicine 2014; 40(12): 1906-15.
17. Barrow G, Clarke TR, Carrington D, Harvey K, Barton EN. An analysis of three opportunistic infections in an outpatient HIV clinic in Jamaica. The West Indian Medical Journal 2010; 59(4): 393-9.
18. Bentley A, Lawrence D, Roche M, Richardson D. Characterising 40 admissions to a specialist HIV inpatient centre: demographics, diagnosis and ideas for service development. Sex Transm Infect 2017;93 (suppl 1): A58–59.
19. Boniatti MM, Pellegrini JAS, Marques LS, et al. Early antiretroviral therapy for HIV-infected patients admitted to an intensive care unit (EARTH-ICU): a randomized clinical trial. PLoS One 2020;15: e0239452.
20. Borges, M. A. S. B., Sales, L. F. S., Terceiro, C. A. E., Filho, J. A. D. A. & Turchi, M. D. Prevalência da antigenemia criptocócica utilizando lateral flow assay (lfa) em pacientes com hiv/aids sintomáticos triados em unidade de referência em goiás. The Brazilian Journal of Infectious Diseases 26, 102143 (2022).
21. Letang E, Rakislova N, Martinez MJ, Carlos Hurtado J, Carrilho C, Bene R et al. Minimally Invasive Tissue Sampling: A Tool to Guide Efforts to Reduce AIDS-Related Mortality in Resource-Limited Settings. Clin Infect Dis 2021;73:S343–S350.
22. Caro-Vega Y, Torres-Guerrero E, Lira-Moreno J, et al. Characteristics 52 of PLWH requiring hospitalization at tertiary healthcare institutions 30 during COVID-19 pandemic in Mexico City. HIV Med 2021;22: 203–04.
23. Chawana R, Baillie V, Izu A, Solomon F, Bassat Q, Blau DM et al. Potential of Minimally Invasive Tissue Sampling for Attributing Specific Causes of Childhood Deaths in South Africa: A Pilot, Epidemiological Study. Clin Infect Dis 2019;69:S361–S373.
24. Chiang HH, Hung CC, Lee CM, et al. Admissions to intensive care unit of HIV-infected patients in the era of highly active antiretroviral therapy: etiology and prognostic factors. Critical Care 2011; 15(4): R202.
25. Collins IJ, Cairns J, Jourdain G, et al. Hospitalization trends, costs, and risk factors in HIV- infected children on antiretroviral therapy. Aids 2012; 26(15): 1943-52.
26. Cordova V, Lopez M, Arteta Z, Correa F. VIH-SIDA en la clínica médica: descripción de una población hospitalaria. Arch Med interna 2009; XXXI;4:99-101.
27. Costales C, Crump JA, Mremi AR, Amsi PT, Kalengo NH, Kilonzo KG et al. Performance of Xpert Ultra nasopharyngeal swab for identification of tuberculosis deaths in northern Tanzania. Clinical Microbiology and Infection 2022;28:1150.e1-1150.e6.
28. Cunha, G. H. D. et al. Mortality, survival and prognostic factors of people with AIDS in intensive care unit. Rev. esc. enferm. USP 55, e20210121 (2021).
29. Dai L, Mahajan SD, Guo C, et al. Spectrum of central nervous system disorders in hospitalized HIV/AIDS patients (2009-2011) at a major HIV/AIDS referral center in Beijing, China. Journal of the Neurological Sciences 2014; 342(1-2): 88-92.
30. Damasceno, L. S., Pessoa, G. M. F., Maia, A. C. C. & Gondim, R. A. M. EVOLUCAO CLINICA DE PACIENTES HIV POSITIVOS EM UMA UNIDADE DE TERAPIA INTENSIVA, NO NORDESTE DO BRASIL. Brazilian Journal of Infectious Diseases 26, 102128 (2022).

31. de Oliveira RB, Atobe JH, Souza SA, de Castro Lima Santos DW. Epidemiology of invasive fungal infections in patients with acquired immunodeficiency syndrome at a reference hospital for infectious diseases in Brazil. Mycopathologia 2014; 178(1-2): 71-8.
32. Dias SS, Andreozzi V, Martins MO, Torgal J. Predictors of mortality in HIV-associated hospitalizations in Portugal: a hierarchical survival model. BMC Health Services Research 2009; 9: 125.
33. Dicko F, Desmonde S, Koumakpai S, et al. Reasons for hospitalization in HIV-infected children in West Africa. Journal of the International AIDS Society 2014; 17: 18818.
34. Dillon, R. et al. Malignancies in HIV infection: A 5 year retrospective analysis. HIV Medicine 22, 166–167 (2021).
35. Dramowski A, Coovadia A, Meyers T, Goga A. Identifying missed opportunities for early intervention among HIV-infected paediatric admissions at Chris Hani Baragwanath Hospital, Soweto, South Africa. Southern Africa Journal of HIV Medicine, December 2011. 16-23.
36. Elkhatiali, E. E. & Jeena, P. M. An evaluation of challenges with the South African PMTCT HIV programme seen from the perspective of HIV-positive children admitted to the PICU. S. Afr. j. child health vol.16 n.1 Pretoria Apr. 2022 http://dx.doi.org/10.7196/sajch.2022.v16i1.1770
37. Falster K, Wand H, Donovan B, et al. Hospitalizations in a cohort of HIV patients in Australia, 1999-2007. Aids 2010; 24(9): 1329-39.
38. Ferrand RA, Bandason T, Musvaire P, et al. Causes of acute hospitalization in adolescence: burden and spectrum of HIV-related morbidity in a country with an early-onset and severe HIV epidemic: a prospective survey. PLoS Medicine 2010; 7(2): e1000178.
39. Fortes Deguenonvo L, Manga NM, Diop SA, et al. [Current profile of HIV-infected patients hospitalized in Dakar (Senegal)]. Bulletin de la Societe de Pathologie Exotique 2011; 104(5): 366-70.
40. Frigati LJ, Brown K, Cotton MF, Myer L, Zar HJ. Hospitalization in South African Adolescents with perinatally acquired HIV on antiretroviral therapy. Pediatr Infect Dis J 2020; 39: 1035–39.
41. Gaillet A, Azoulay E, de Montmollin E, et al. Outcomes in critically Ill HIV-infected patients between 1997 and 2020: analysis of the OUTCOMEREA multicenter cohort. Crit Care 2023; 27: 108.
42. Gama, W. M. et al. Immunologic biomarkers, morbidity and mortality among HIV patients hospitalised in a Tertiary Care Hospital in the Brazilian Amazon. BMC Infect Dis 21, 876 (2021).
43. Gel G, Kitaka SB, Musiime V. Prevalence, clinical pattern and immediate outcomes of HIV-infected children admitted to Al Sabah Children’s Hospital, South Sudan. South Sudan Med J 2019; 12: 85–88.
44. Gonzalez-Fernandez E, Medina-Pinon I, Ramos-Jimenez J. 35 Advanced presentation among HIV/AIDS patients despite universal access to antiretroviral therapy in northern Mexico. J Int AIDS Soc 55 2018; 21: 26–27.
45. Guedes, D. L. et al. Visceral Leishmaniasis in Hospitalized HIV-Infected Patients in Pernambuco, Brazil. The American Journal of Tropical Medicine and Hygiene 99, 1541–1546 (2018).
46. Guerro AC, Andretta IB, Bello SL, Trevisol DJ, Schuelter-Trevisol F. Causes of hospital admission of AIDS patients in southern Brazil, 2007 to 2012. Revista da Sociedade Brasileira de Medicina Tropical 2014; 47(5): 632-6.
47. Guillén A, Siso R, Comegna M. Causas de muerte en pacientes infectados con VIH en el año 2017. Hospital Vargas de Caracas.
48. Gupta-Wright, A. et al. Rapid urine-based screening for tuberculosis in HIV-positive patients admitted to hospital in Africa (STAMP): a pragmatic, multicentre, parallel-group, double-blind, randomised controlled trial. The Lancet 392, 292–301 (2018).
49. Haachambwa, L. et al. Care Continuum and Postdischarge Outcomes Among HIV-Infected Adults Admitted to the Hospital in Zambia. Open Forum Infect Dis 6, ofz336 (2019).
50. Hajiabdolbaghi M, Jafari S, Mansouri S, Hedayat Yaghoobi M. Hospitalizations and its related factors in HIV/AIDS patients in Tehran, Iran. Medical journal of the Islamic Republic of Iran 2014; 28: 70.
51. Hoffmann CJ, Milovanovic M, Kinghorn A, et al. Readmission and death following hospitalization among people with HIV in South Africa. PLoS ONE 2019; 14: e0218902."
52. Huerga, H. et al. Urine Lipoarabinomannan Testing for All HIV Patients Hospitalized in Medical Wards Identifies a Large Proportion of Patients With Tuberculosis at Risk of Death. Open Forum Infectious Diseases 8, ofaa639 (2021).
53. Japiassu AM, Amancio RT, Mesquita EC, et al. Sepsis is a major determinant of outcome in critically ill HIV/AIDS patients. Critical Care 2010; 14(4): R152.
54. Johnson SM, Kaudki S, Rakhit D, et al. Hospitalisation across the ages: transitioning young people with perinatally acquired HIV (PaHIV). HIV Med 2020; 21: 54–55.
55. Juniper T, Oliveira A, Childs K, Hamlyn E. The majority of HIV inpatient admissions in south London occur in previously diagnosed patients: is poor engagement in care the final hurdle? HIV Med 2020; 21: 56.
56. Kang MW, Kim YJ, Kim SI, Korean K. Opportunistic diseases among HIV-infected patients: Korea HIV/AIDS Cohort study, 2006-2013. International Journal of Infectious Diseases. Conference: 16th International Congress on Infectious Diseases, 2014.
57. Kanyama, C. et al. Implementation of tuberculosis and cryptococcal meningitis rapid diagnostic tests amongst patients with advanced HIV at Kamuzu Central Hospital, Malawi, 2016-2017. BMC Infect Dis 22, 224 (2022).
58. Kazibwe, A. et al. HIV, tuberculosis, diabetes mellitus and hypertension admissions and premature mortality among adults in Uganda from 2011 to 2019: is the tide turning? Trop Med Health 50, 54 (2022).
59. Kendig CE, McCulloch DJ, Rosenberg NE, et al. Prevalence of HIV and Disease Outcomes on the Medical and Surgical Wards at Kamuzu Central Hospital, Lilongwe, Malawi. Tropical Medicine and Health 2013; 41(4): 163-70.
60. Kim JH, Psevdos G, Jr., Gonzalez E, Singh S, Kilayko MC, Sharp V. All-cause mortality in hospitalized HIV-infected patients at an acute tertiary care hospital with a comprehensive outpatient HIV care program in New York City in the era of highly active antiretroviral therapy (HAART). Infection 2013; 41(2): 545-51.
61. Peoples Friendship University of Russia (RUDN University), Moscow, Russia et al. Opportunistic diseases in patients with HIV infection in the intensive care unit. Ther. Arch. 90, 13–17 (2018).
62. Kra O, Aba YT, Yao KH, et al. [Clinical, biological, therapeutic and evolving profile of patients with HIV infection hospitalized at Infectious and tropical diseases unit in Abidjan (Ivory Coast)]. Bulletin de la Societe de Pathologie Exotique 2013; 106(1): 37-42.

63. Krutikov M, Parry S, Drury L, Matin N, Orkin C. HIV-related medical admissions to an HIV specialist inpatient unit: Quality standards and outcomes. HIV Med 2018; 19: S102–03.
64. Kwara A, Shah D, Renner LA. Outcome of hospital admissions in HIV-infected children at the Korle Bu Teaching Hospital, Accra, Ghana. West African Journal of Medicine 2010; 29(6): 379-83.
65. Laher AE, Paruk F, Richards GA, Venter WDF. Predictors of prolonged hospital stay in HIV-positive patients presenting to the emergency department. PLoS One 2021; 16: e0249706.
66. Lakoh S, Rickman H, Sesay M, et al. Prevalence and mortality of cryptococcal disease in adults with advanced HIV in an urban tertiary hospital in Sierra Leone: a prospective study. BMC Infect Dis 2020; 20: 141.
67. Lara-Medrano R, Sánchez-Domínguez KE, Sánchez-Landeros A, et al. Prolonged hospital stays and associated factors in patients receiving care in a HIV/AIDS clinic in Mexico City. J Int AIDS Soc 2018; 21: 30–31.
68. Laso, E. et al. Analysis of HIV patients hospitalization, clinical situation and related factors. International Journal of Clinical Pharmacy 39, 293 (2017).
69. Leonard M, Figueroa-Sierra M, Kindlick D. Late presentation among patients diagnosed with HIV in an inpatient setting. Open Forum Infect Dis 2017; 4 (suppl 1): S423.
70. Lewden C, Drabo YJ, Zannou DM, et al. Disease patterns and causes of death of hospitalized HIV-positive adults in West Africa: a multicountry survey in the antiretroviral treatment era. Journal of the International AIDS Society 2014; 17: 18797.
71. Li CB, Zhou Y, Wang Y, Liu S, Wang W, Lu X et al. In-hospital Mortality and Causes of Death in People Diagnosed With HIV in a General Hospital in Shenyang, China: A Cross-Sectional Study. Front Public Health 2021;9:774614.
72. Lima RCR de O. Mortalidade, sobrevida e fatores prognósticos de pessoas com AIDAS em Unidade de Terapia Intensiva. 2018;110–110.
73. Lucero C, Torres B, Leon A, et al. Rate and predictors of non-AIDS events in a cohort of HIV- infected patients with a CD4 T cell count above 500 cells/mm(3). AIDS Research and Human Retroviruses 2013; 29(8): 1161-7.
74. Luz PM, Bruyand M, Ribeiro S, et al. AIDS and non-AIDS severe morbidity associated with hospitalizations among HIV-infected patients in two regions with universal access to care and antiretroviral therapy, France and Brazil, 2000-2008: hospital-based cohort studies. BMC Infectious Diseases 2014; 14: 278.
75. Maheswaran H, Petrou S, Cohen D, et al. Economic costs and health-related quality of life outcomes of hospitalised patients with high HIV prevalence: a prospective hospital cohort study in Malawi. PLoS One 2018; 13: e0192991.
76. Maphula, R., Laher, A. & Richards, G. Patterns of presentation and survival of HIV-infected patients admitted to a tertiary- level intensive care unit. HIV Medicine 21, 334–341 (2020).
77. Masoza TS, Rwezaula R, Msanga DR, et al. Prevalence and outcome of HIV infected children admitted in a tertiary hospital in northern Tanzania. BMC Pediatr 2022; 22: 101.
78. Matin N, Shahrin L, Pervez MM, et al. Clinical profile of HIV/AIDS-infected patients admitted to a new specialist unit in Dhaka, Bangladesh--a low-prevalence country for HIV. Journal ofHealth, Population, and Nutrition 2011; 29(1): 14-9.
79. Maxwell A, Cormack I, Barbour A. A changing pattern of HIV inpatient admissions and complexity: from late diagnoses to defaulters. HIV Med 2019; 20: 46–47.
80. Medrano J, Alvaro-Meca A, Boyer A, Jimenez-Sousa MA, Resino S. Mortality of patients infected with HIV in the intensive care unit (2005-2010): significant role of chronic hepatitis C and severe sepsis. Critical Care 2014; 18(4): 475.
81. Meng, S. et al. Spectrum and mortality of opportunistic infections among HIV/AIDS patients in southwestern China. Eur J Clin Microbiol Infect Dis 42, 113–120 (2023).
82. Metallidis S, Tsachouridou O, Skoura L, et al. Older HIV-infected patients--an underestimated population in northern Greece: epidemiology, risk of disease progression and death. International Journal of Infectious Diseases 2013; 17(10): e883-91.
83. Meyers T, Dramowski A, Schneider H, Gardiner N, Kuhn L, Moore D. Changes in pediatric HIV-related hospital admissions and mortality in Soweto, South Africa, 1996-2011: light at the end of the tunnel? Journal ofAcquired Immune Deficiency Syndromes 2012; 60(5): 503-10.
84. Miranda A, Fernandes D, Peres S, et al. Hospital admissions of HIV-infected patients at a Lisbon reference centre: Comparison among previously known and in-ward HIV-diagnosed patients. Journal of the International AIDS Society. Conference: 11th International Congress on Drug Therapy in HIV Infection Glasgow United Kingdom. 2012.
85. Mishore KM, Hussein N, Huluka SA. Hospitalization and predictors of inpatient mortality among HIV-infected patients in Jimma University Specialized Hospital, Jimma, Ethiopia: prospective observational study. Aids Res Treat 2020; 2020: 1872358.
86. Moreira-Silva SF, Zandonade E, Frauches DO, et al. Comorbidities in children and adolescents with AIDS acquired by HIV vertical transmission in Vitoria, Brazil. PloS ONE 2013; 8(12): e82027.
87. Morquin D, Le Moing V, Mura T, et al. Short- and long-term outcomes of HIV-infected patients admitted to the intensive care unit: impact of antiretroviral therapy and immunovirological status. Annals of Intensive Care 2012; 2(1): 25.
88. Mulu H, Hamza L, Alemseged F. Prevalence of malnutrition and associated factors among hospitalized patients with acquired immunodeficiency syndrome in Jimma University Specialized Hospital, Ethiopia. Ethiop J Health Sci 2016; 26: 217–26.
89. Naidoo VA, Martinson NA, Moodley P, et al. HIV prevalence and morbidity in older inpatients in a high HIV prevalence setting. AIDS Res Hum Retroviruses 2020; 36: 186–92.
90. Namutebi AM, Kamya MR, Byakika-Kibwika P. Causes and outcome of hospitalization among HIV-infected adults receiving antiretroviral therapy in Mulago hospital, Uganda. African Health Sciences 2013; 13(4): 977-85.
91. Nascimento L, Improta-Caria AC, Brites C. Mortality in hospitalized HIV-infected patients in a referral center in Bahia, Brazil. The Brazilian Journal of Infectious Diseases 2022;26:102716.
92. Neto NB, Marin LG, de Souza BG, Moro AL, Nedel WL. HIV treatment non-adherence is associated with ICU mortality in HIV- positive critically ill patients. J Intensive Care Soc 2021; 22: 47–51.
93. Njuguna IN, Cranmer LM, Otieno VO, et al. Urgent versus post-stabilisation antiretroviral treatment in hospitalised HIV-infected children in Kenya (PUSH): a randomised controlled trial. Lancet HIV 2018; 5: e12–22.

94. Nyandiko WM, Mwangi A, Ayaya SO, et al. Characteristics of HIV-infected children seen in Western Kenya. East African Medical Journal 2009; 86(8): 364-73.
95. Ogoina D, Obiako RO, Muktar HM, et al. Morbidity and Mortality Patterns of Hospitalised Adult HIV/AIDS Patients in the Era of Highly Active Antiretroviral Therapy: A 4-year Retrospective Review from Zaria, Northern Nigeria. AIDS Research and Treatment 2012; 2012: 940580.
96. Ondounda M, Magne C, Mounguengui D, Gaudong Mbethe L, Nzenze JR. [Morbidity and mortality in HIV-infected patients in the Military Hospital in Libreville (Gabon)]. Medecine et Sante Tropicales 2012; 22(3): 334-5.
97. Ousley J, Niyibizi AA, Wanjala S, et al. High Proportions of Patients With Advanced HIV Are Antiretroviral Therapy Experienced: Hospitalization Outcomes From 2 Sub-Saharan African Sites. Clin Infect Dis 2018; 66: S126-31."
98. Pandharpurkar, D., Devulapally, N., Gouthami, B. & Krishna, G. Spectrum of opportunistic infections in relation to CD4 counts in HIV/AIDS patients admitted in the department of general medicine of a tertiary care hospital. Int J Adv Med 6, 845 (2019).
99. Parry S, Williamson M, Orkin C, Matin N, Dhairyawan R. HIV-related admissions to a London specialist unit: who, what and why? HIV Med 2020; 21: 54.
100. Paudel BN, Dhungana GP. Scenario of HIV/AIDS patients in a government hospital of Nepal. Journal of Nepal Health Research Council 2010; 8(2): 103-6.
101. Raga Almudever J, Cervero M, Torres R, et al. Changing pattern of hospital admissions due to medical conditions in HIV-infected subjects in a European public health care system with free access to antiretroviral treatment. HIV Med 2019; 20: 202.
102. Rapp C, Reggad A, Aoun A, Ficko C, Andriamanantena D, Flateau C. Hospitalisation causes of HIV-infected patients in 2011 in an HIV reference center in the Paris region, France. Journal of the International AIDS Society. Conference: 11th International Congress on Drug Therapy in HIV Infection Glasgow United Kingdom. 2012.
103. Raubenheimer PJ, Day C, Abdullah F, Manning K, Cupido C,
104. Peter J. The utility of a shortened palliative care screening tool to predict death within 12 months—a prospective observational study in two south African hospitals with a high HIV burden. BMC Palliat Care 2019; 18: 101.
105. Ribeiro SR, Luz PM, Campos DP, et al. Incidence and determinants of severe morbidity among HIV-infected patients from Rio de Janeiro, Brazil, 2000-2010. Antiviral Therapy 2014; 19(4): 387-97.
106. Ruiz GO, Herrera CFL, Bohórquez JAM, Betancur JE. Mortality in patients with acquired human immunodeficiency virus infection hospitalized in an intensive care unit during the period 2017–2019. Sci Rep 2022; 12: 15644.
107. Rukhadze N, Kirk O, Chkhartishvili N, et al. Causes and outcomes of hospitalizations among people living with HIV in Georgia’s referral institution, 2012–2017. Int J STD AIDS 2021; 32: 662–70.
108. Saldarriaga-Arenas PA, Rodriguez-Morales AJ. Epidemiology of opportunistic diseases in AIDS patients from Pereira municipality, Colombia, 2010-2011. Journal of Infection and Public Health 2013; 6(6): 496- 8.
109. Santos AS, Ferreira A, Pineiro C, et al. Epidemiology of HIV-infected patients admitted to an intensive care unit: A 20-year study. Intensive Care Medicine. Conference: 25th Annual Congress of the European Society of Intensive Care Medicine, ESICM 2012 Lisbon Portugal. 2012.
110. Schlabe S, Kuhlmann A, Schmidt K, et al. Analysis of the inpatient care spectrum of patients with HIV infection in the infectiology department of a tertiary referral centre before and during the COVID-19 pandemic. HIV Med 2023; 24: 93–94.
111. Senoglu, S., Yesilbag, Z., Karaosmanoglu, H. K. & Aydin, O. A. Epidemiological differences and risk factors for hospitalization in people living with HIV in Istanbul, Turkey. International journal of STD & AIDS 30, 1284–1289 (2019).
112. Shahrin L, Leung DT, Matin N, Kawser CA, Pervez MM, Chisti MJ. Clinical profile of hospitalized HIV-infected children in Bangladesh, a low-HIV-prevalence country. Paediatrics and International Child Health 2014; 34(2): 133-7.
113. Shrosbree J, Campbell LJ, Ibrahim F, et al. Late HIV diagnosis is a major risk factor for intensive care unit admission in HIV-positive patients: a single centre observational cohort study. BMC Infectious Diseases 2013; 13: 23.
114. Sture G, Rozentale B, Zeltina I, Januskevica I, Sangirejeva A. Reasons of hospitalization for HIV- positive patients in the Infectology Center of Latvia in the period from 2009 to 2011. Abstracts of the Eleventh International Congress on Drug Therapy in HIV Infection. Journal of the International AIDS Society 2012.15;Suppl 4:18405.
115. T Sudjaritruk, P Oberdorfer, T Puthanakit, T Sirisanthana and V Sirisanthana. Causes of first hospitalization among 1121 HIV-infected children: comparison of the pre-Pneumocystis jiroveci pneumonia prophylaxis, pre-antiretroviral therapy and antiretroviral therapy periods. Int J STD AIDS 2012 23: 335.
116. Beckwith PG, Tlali M, Charalambous S, et al. Causes and outcomes 5 42 of admission and investigation of tuberculosis in adults with advanced HIV in South African hospitals: data from the TB Fast Track Trial. Am J Trop Med Hyg 2021; 105: 1662–71.
117. Karat AS, Omar T, von Gottberg A, Tlali M, Chihota VN, Churchyard GJ et al. Autopsy Prevalence of Tuberculosis and Other Potentially Treatable Infections among Adults with Advanced HIV Enrolled in Out-Patient Care in South Africa. PLoS One 2016;11. doi:10.1371/journal.pone.0166158.
118. Tepungipame AT, Tonen-Wolyec S, Kalla GC, et al. Predictors of AIDS-related death among adult HIV-infected inpatients in Kisangani, the Democratic Republic of Congo. Pan Afr Med J 2020; 44 37: 144.
119. Thinyane K, Cooper V. Clinical Profiles of HIV-Infected, HAART-Naive Patients Admitted to a Tertiary Level Hospital in Maseru, Lesotho. The Internet Journal of Infectious Diseases; 11: 1.
120. Thit, S. S. et al. The clinical utility of the urine-based lateral flow lipoarabinomannan assay in HIV-infected adults in Myanmar: an observational study. BMC Med 15, 145 (2017).
121. Thompson LH1, Sochocki M, Friesen T, Bresler K, Keynan Y, Kasper K, Becker M. Medical ward admissions among HIV-positive patients in Winnipeg, Canada, 2003-10. International journal of STD & AIDS 2012 23: 287.
122. Tittle V, Cenderello G, Pasa A, et al. A comparison of inpatient admissions in 2012 from two European countries. Journal of the International AIDS Society 2014; 17(4 Suppl 3): 19712.
123. Traore AM, Minta DK, Fomba M, et al. [Epidemiological, clinical and evolving HIV-positive patients referred to the University Hospital of Point G, Bamako, Mali.]. Bulletin de la Societe de Pathologie Exotique 2013.

124. Umeta GT, Chelkeba L, Tefera GM, Jemal K, Goro KK. Causes and 1 40 predictors of hospitalization and in-hospital mortality among HIV/AIDS patients on highly active antiretroviral therapy in secondary and tertiary care hospitals in Oromia Regional State: multi-center cross-sectional study. HIV AIDS Rev 2021; 20: 102–08.
125. Vega Carlos PV, Ana G, Severis C, Estiben S, Joseph G, Manuel G et al. Mortalidad en pacientes hospitalizados con diagnóstico de virus de inmunodeficiencia humana.
126. Viani RM, Araneta MR, Lopez G, Chacon-Cruz E, Spector SA. Clinical Outcomes and Hospitalizations among Children Perinatally Infected with HIV-1 in Baja California, Mexico. Journal of the International Association of Physicians in AIDS Care 2011; 10(4): 223-8.
127. Vidal JE, Toniolo C, Paulino A, et al. Asymptomatic cryptococcal antigen prevalence detected by lateral flow assay in hospitalised HIV-infected patients in São Paulo, Brazil. Trop Med Int Health 2016; 21: 1539–44.
128. Vidal-Cortes P, Lameiro-Flores P, Mourelo-Farina M, Aller-Fernandez A, Gomez-Lopez R, Fernandez-Ugidos P, Alves-Perez M, Rodriguez-Garcia E. Sepsis in HIV patients admitted to the ICU. Biomedical Research 2012. 23: 139-142.
129. Wen Y, Zhou Y, Wang W, et al. Baseline factors associated with mortality within six months after admission among hospitalized HIV-1 patients in Shenyang, China. Internal medicine 2014; 53(21): 2455-61.
130. Whitehorn J, Edwards SG, Cartledge JD, Miller RF. Outcome of HIV-infected patients transferred to a specialist inpatient unit. International journal of STD & AIDS 2011; 22(4): 225-7.
131. Xiao J, Gao G, Li Y, et al. Spectrums of opportunistic infections and malignancies in HIV- infected patients in tertiary care hospital, China. PloS one 2013; 8(10): e75915.
132. du Plooy E, Frigati L, Slogrove A, Cotton MF, Rabie H. Profile of young South African children hospitalized with HIV: cause for concern. Pediatr Infect Dis J 2020; 39: 840–42.
133. van Schalkwyk C, Mahy M, Johnson LF, Imai-Eaton JW. Updated data and methods for the 2023 UNAIDS HIV estimates.J Acquir Immune Defic Syndr 2024; 95 (suppl): e1–4.

S Figure 7: Forest plot for estimate of proportion of deaths due to AIDS

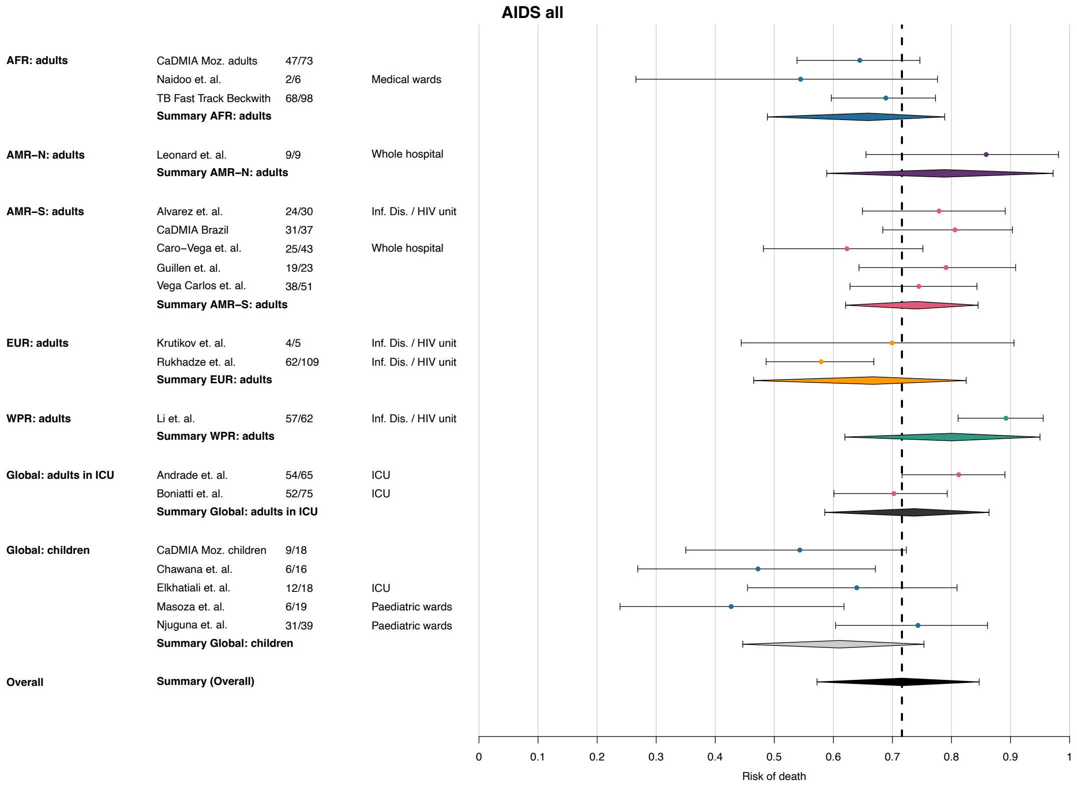

S Figure 8: Forest plot for estimate of proportion of deaths due to TB

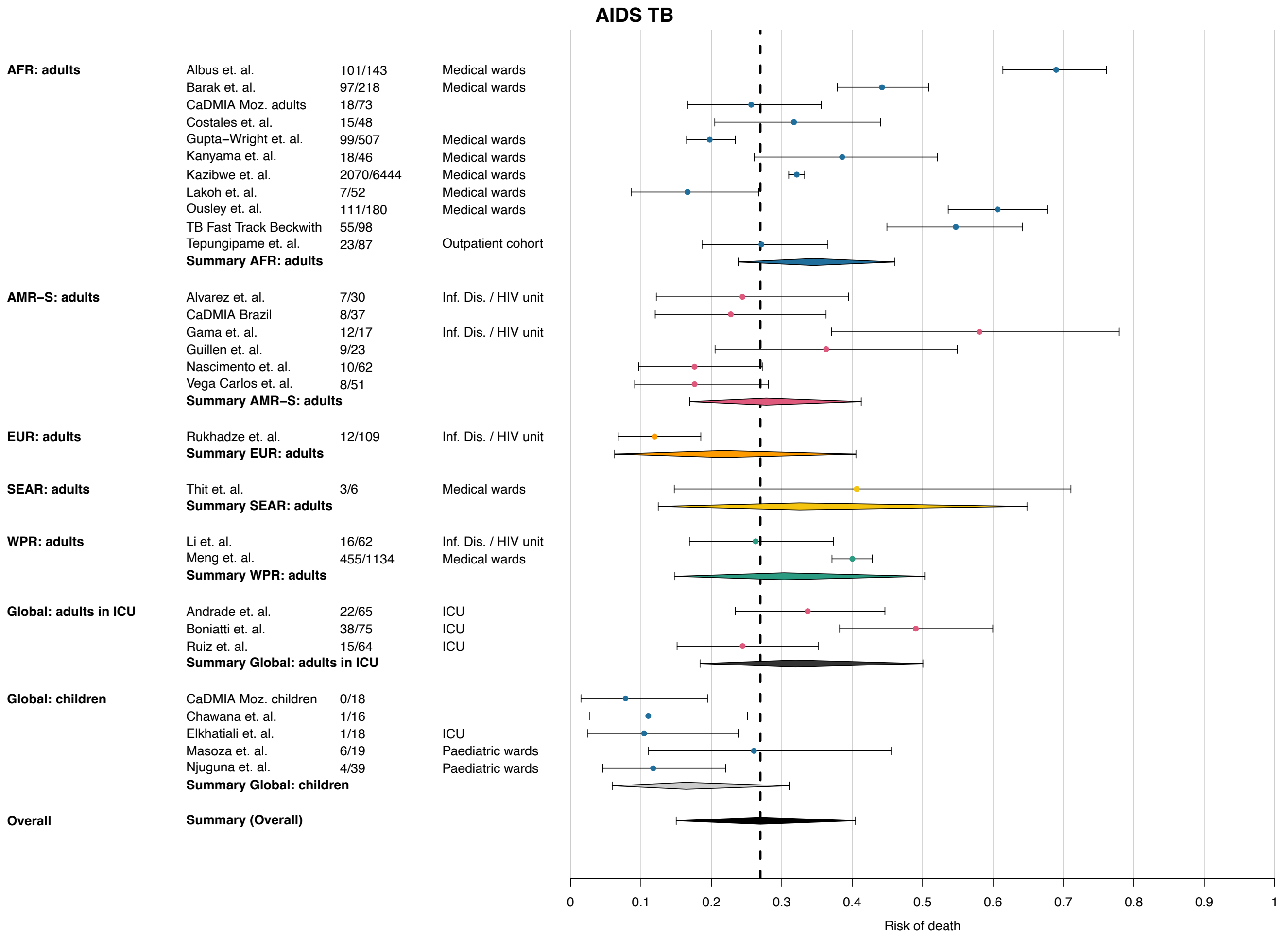

S Figure 9: Forest plot for estimate of proportion of deaths due to cryptococcal disease

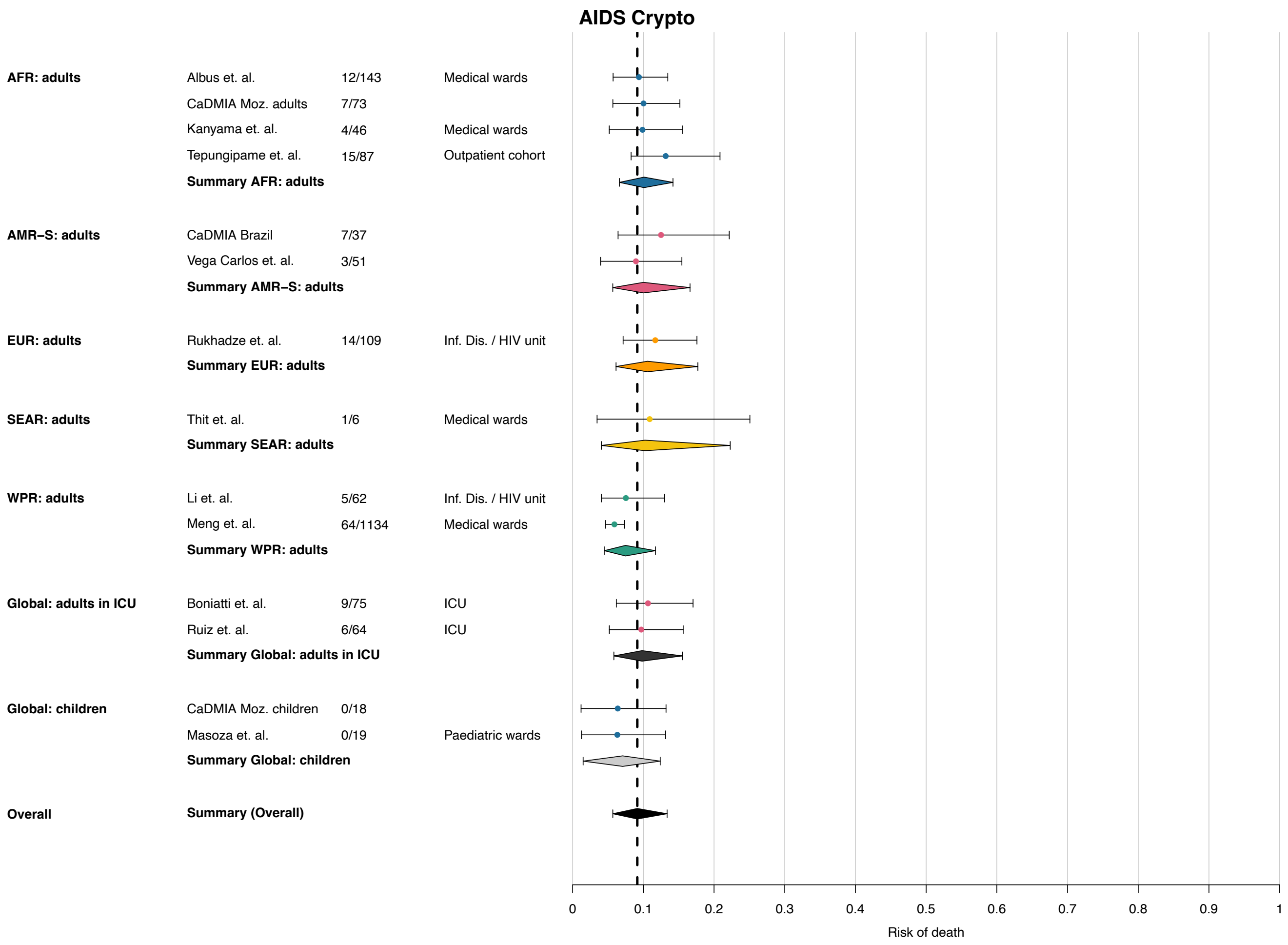

S Figure 10: Forest plot for estimate of proportion of deaths due to Pneumocystis jirovecci pneumonia (PJP)

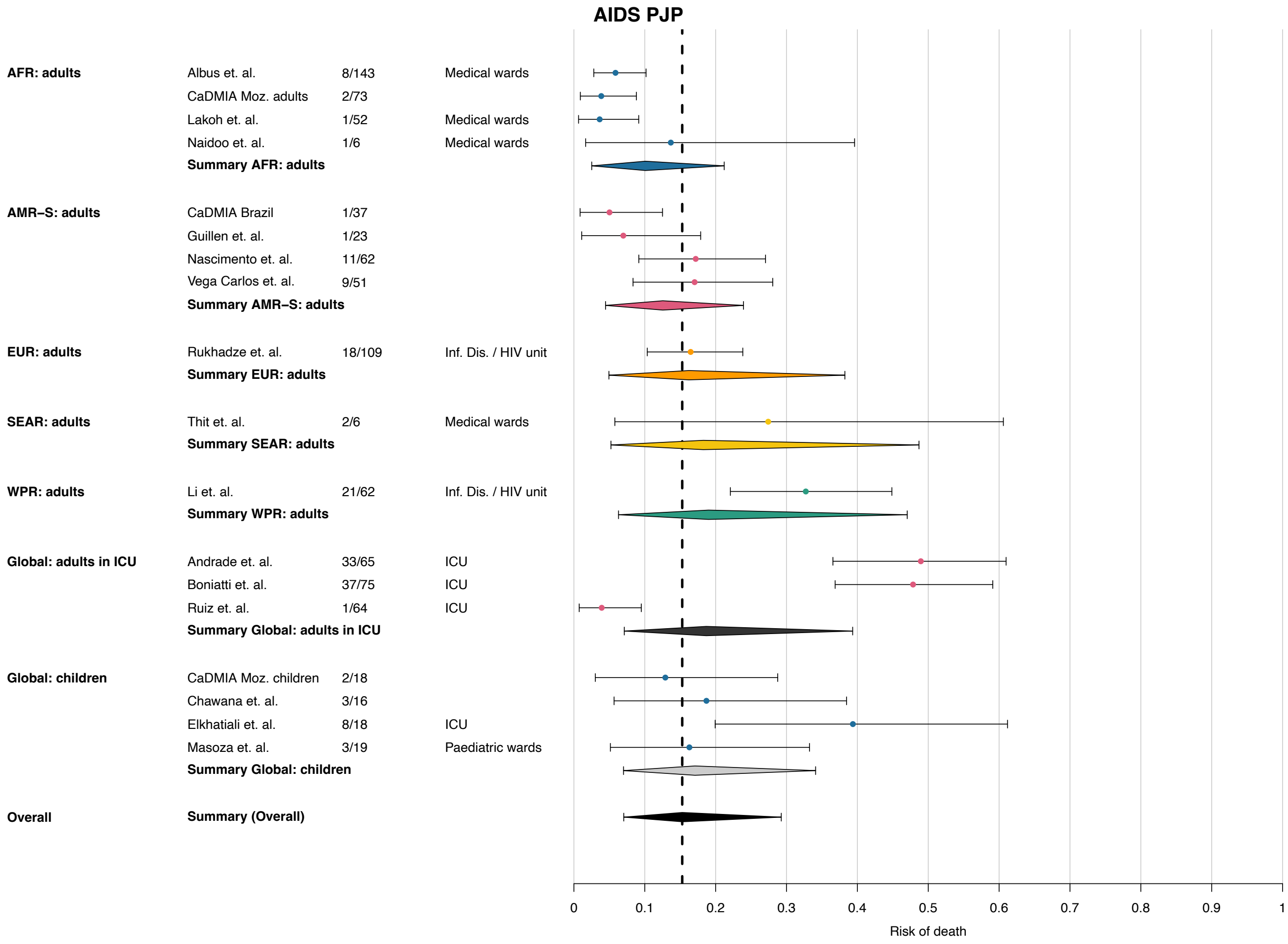

S Figure 11: Forest plot for estimate of proportion of deaths due to toxoplasmosis

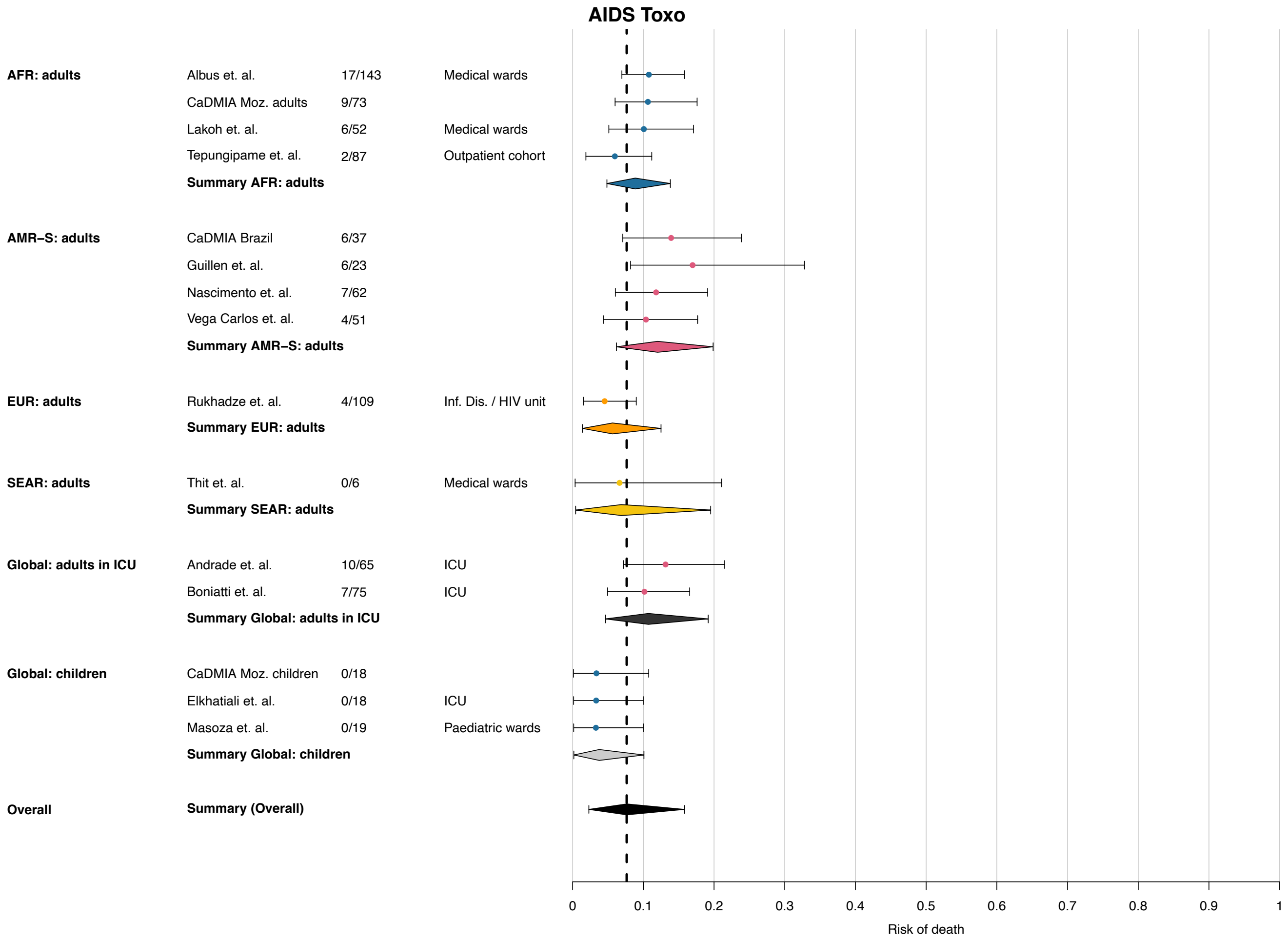

S Figure 12: Forest plot for estimate of proportion of deaths due to AIDS related malignancies

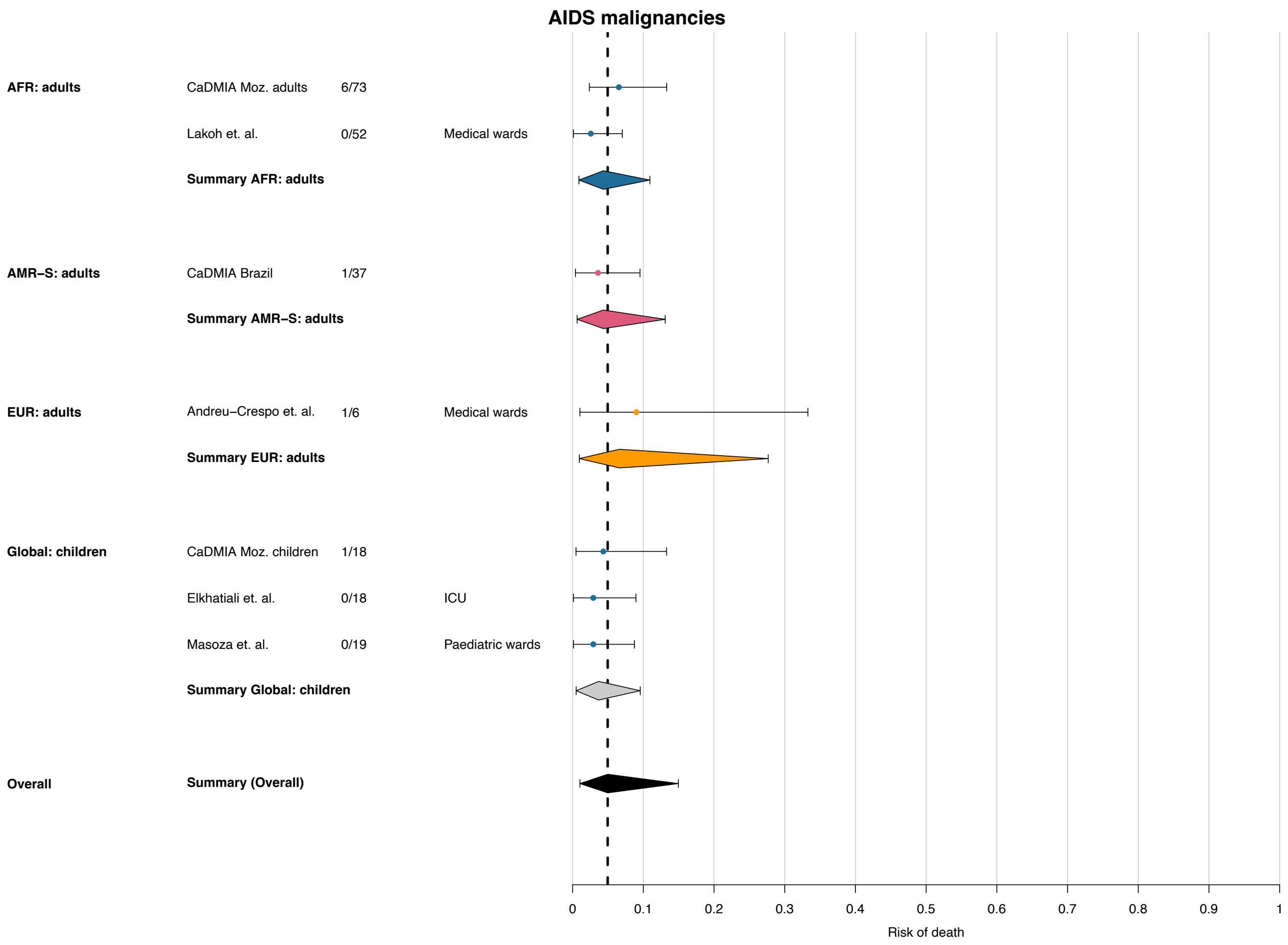

S Figure 13: Forest plot for estimate of proportion of deaths due to CMV disease

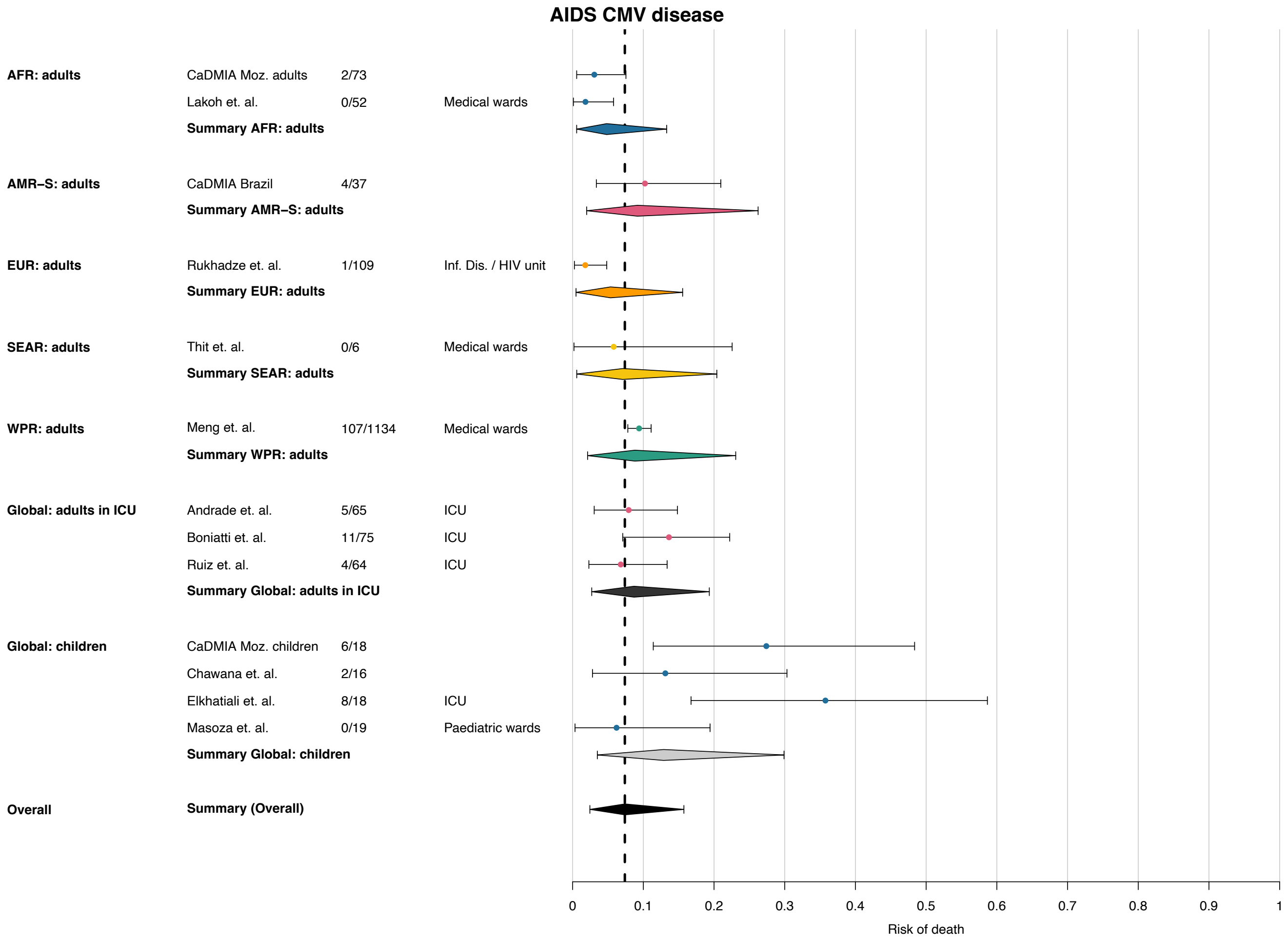

S Figure 14: Forest plot for estimate of proportion of deaths due to histoplasmosis

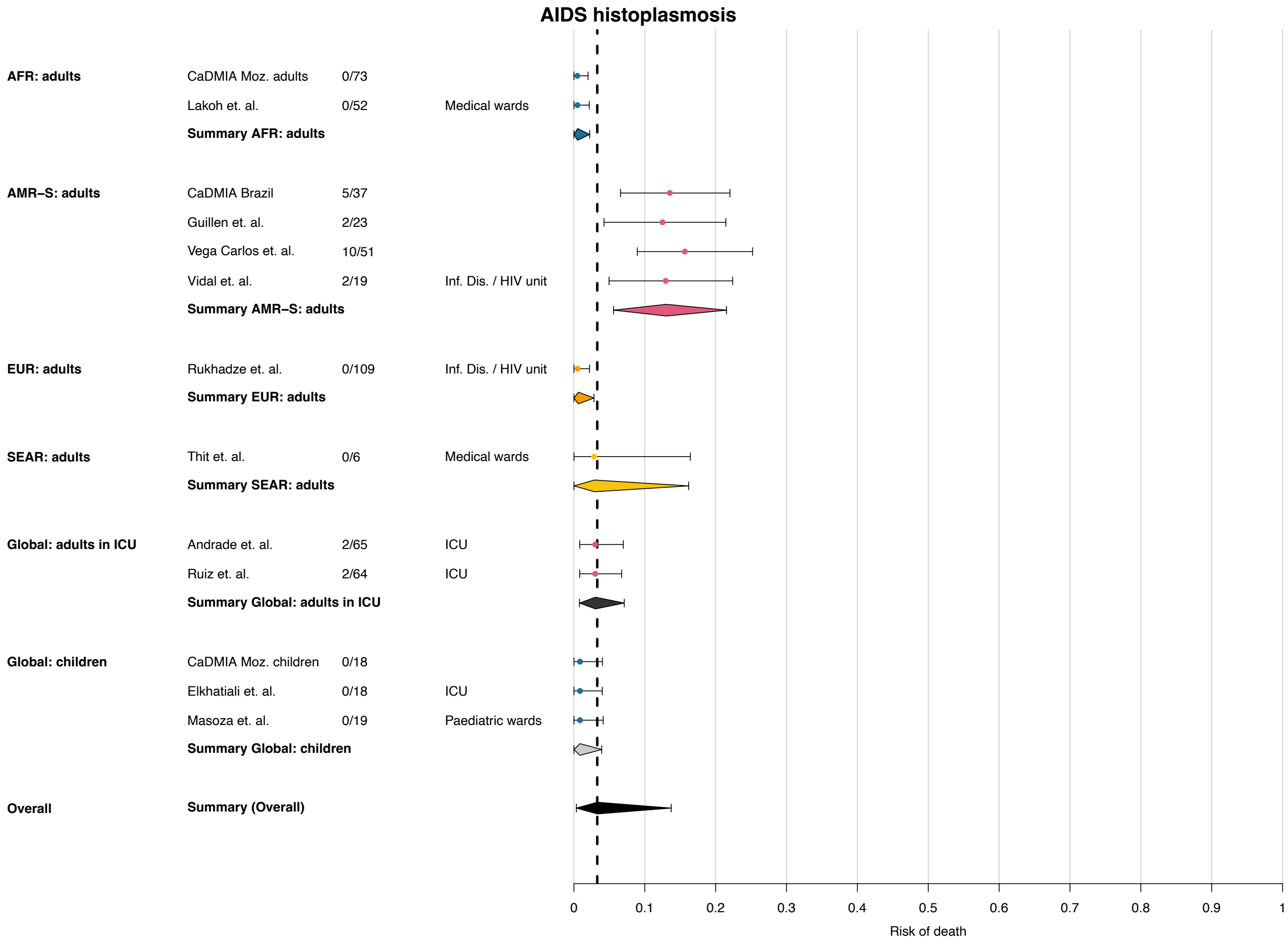

S Figure 15: Forest plot for estimate of proportion of deaths due to bacterial infections (all)

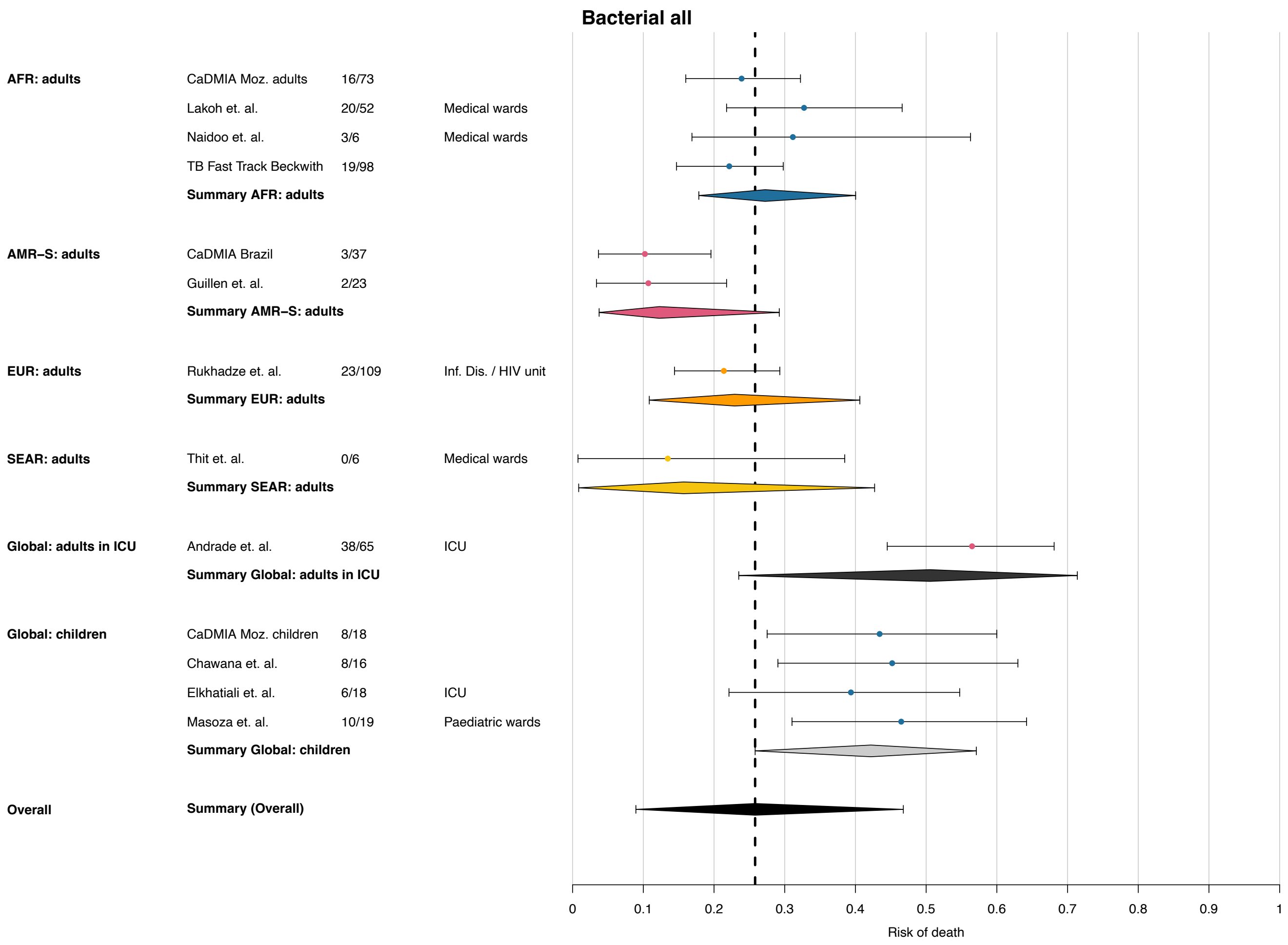

S Figure 16: Forest plot for estimate of proportion of deaths due to bacterial pneumonia

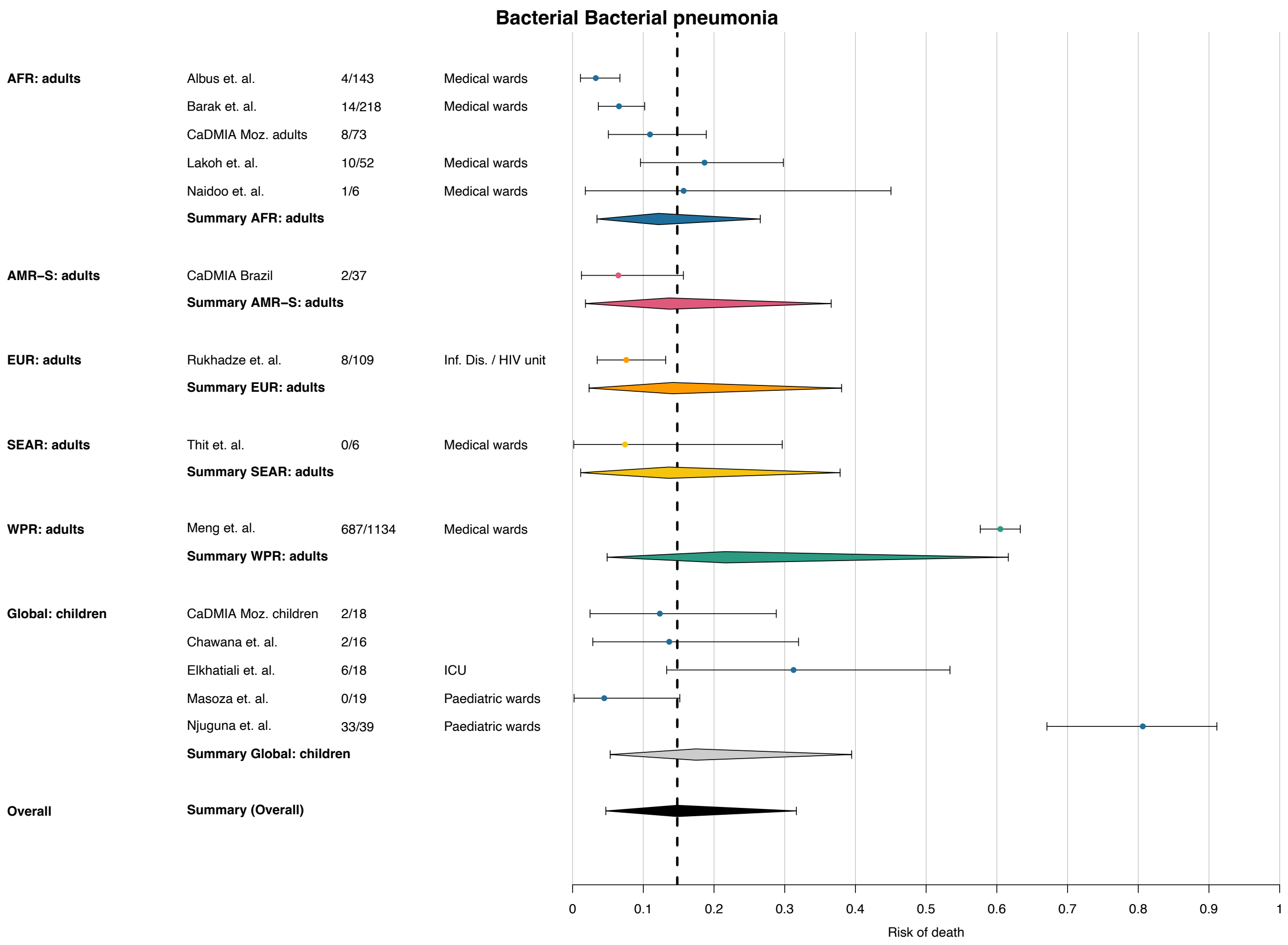

S Figure 17: Forest plot for estimate of proportion of deaths due to bacterial meningitis

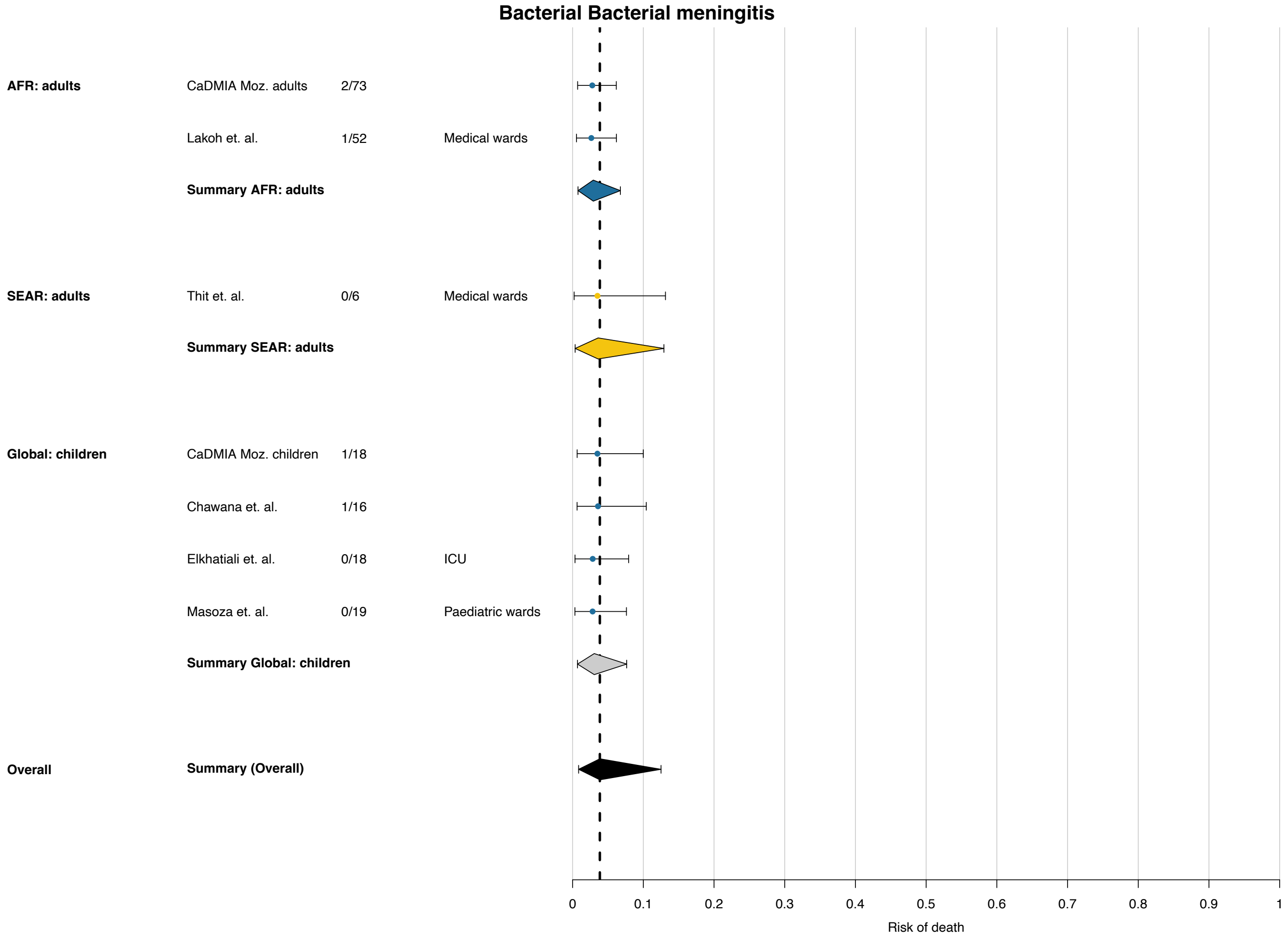

S Figure 18: Forest plot for estimate of proportion of deaths due to bacterial diarrhoea

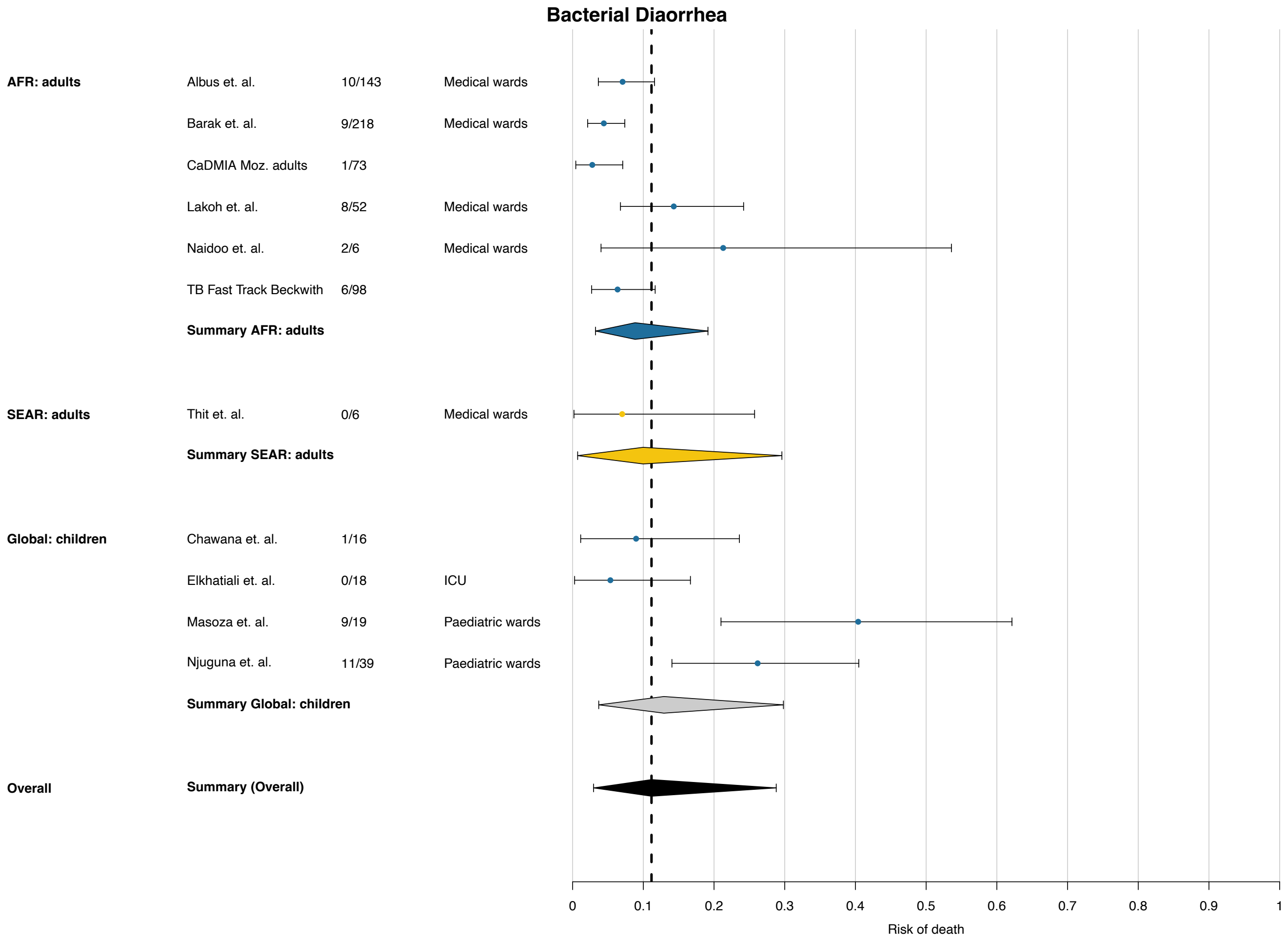

S Figure 19: Forest plot for estimate of proportion of deaths due to malnutrition / wasting

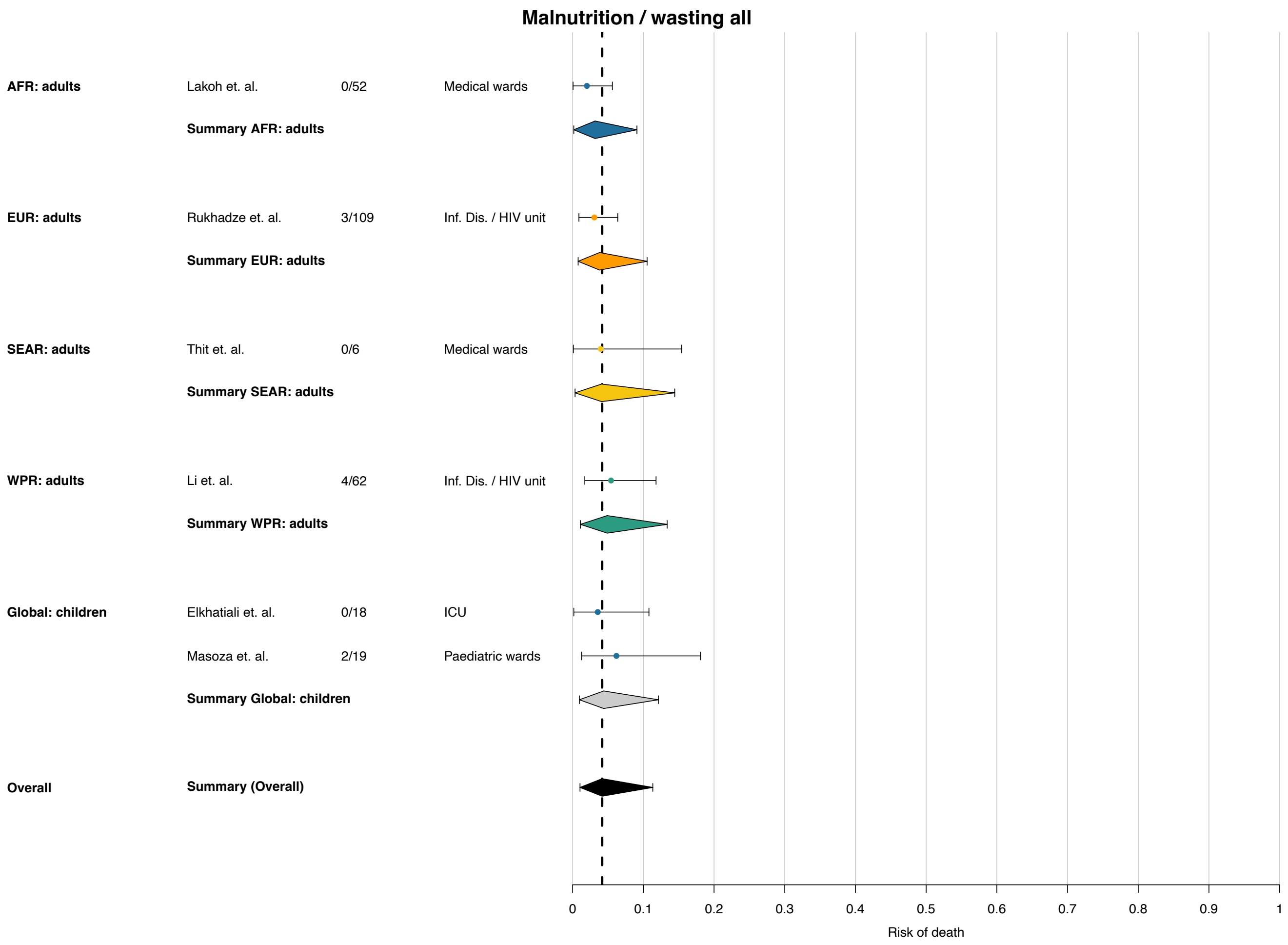

S Figure 20: Forest plot for estimate of proportion of deaths due to parasitic infections (all)

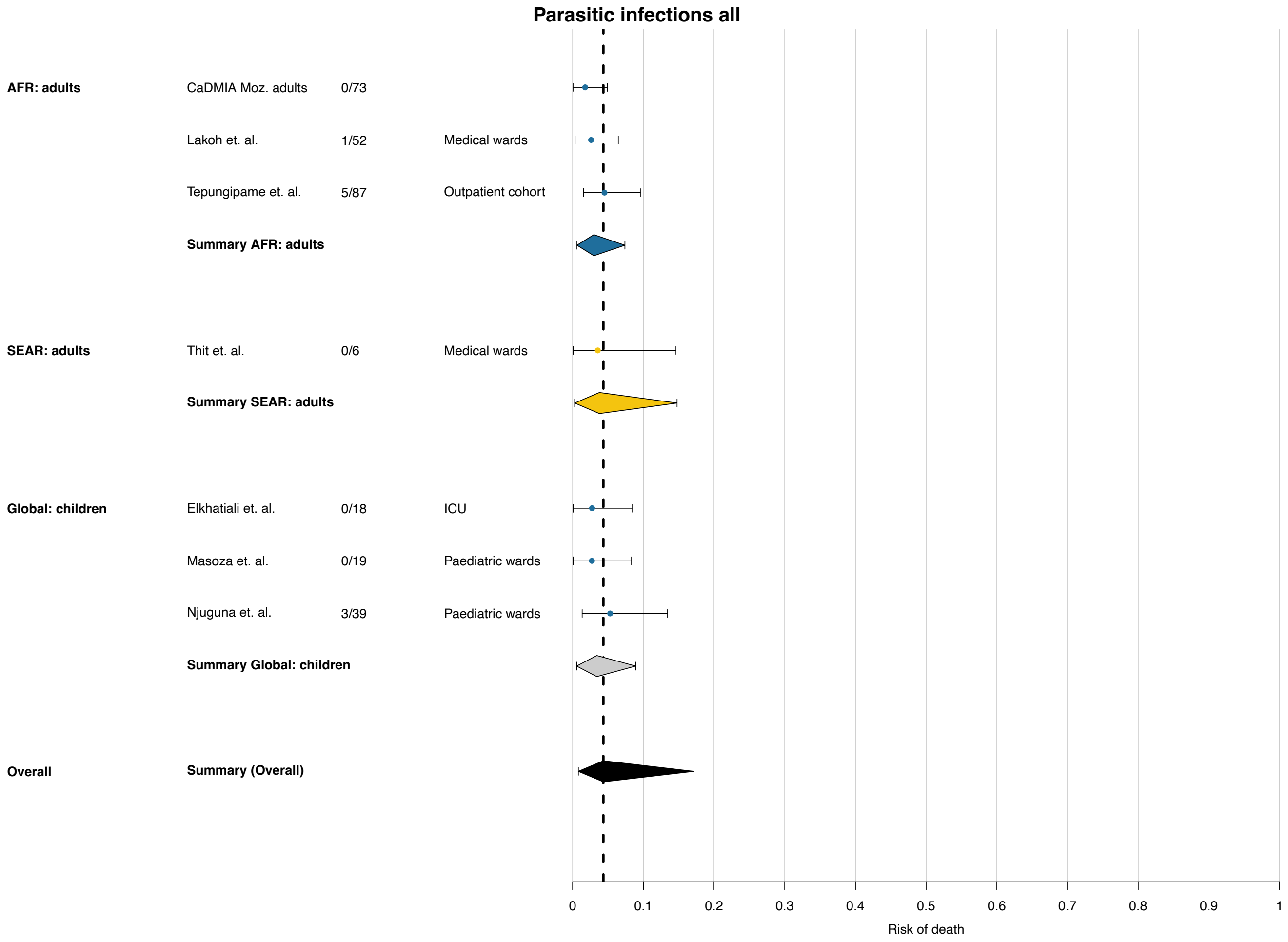

S Figure 21: Forest plot for estimate of proportion of deaths due to malignancies, other than AIDS related malignancies

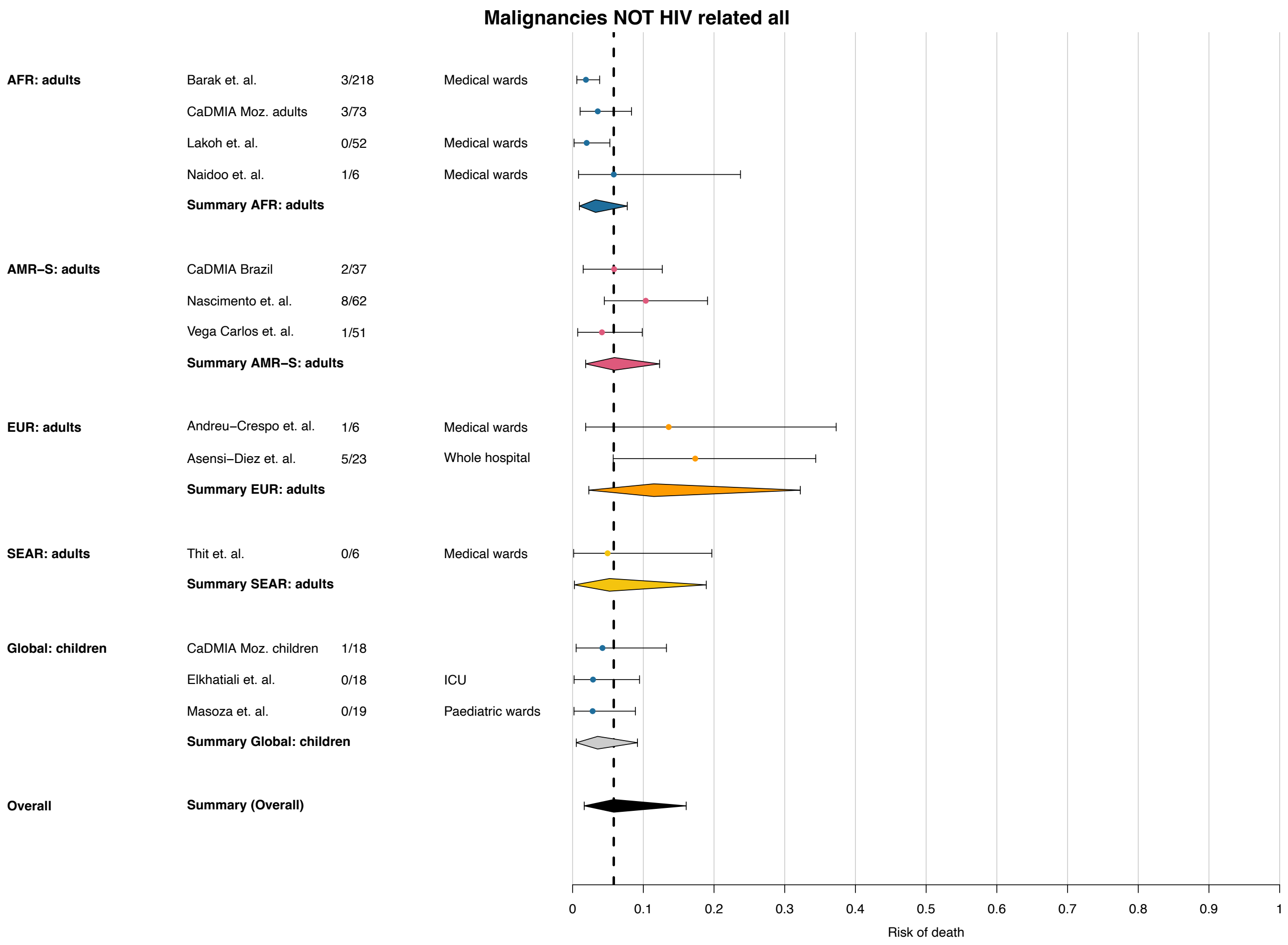

S Figure 22: Forest plot for estimate of proportion of deaths due to cardiovascular disease (all)

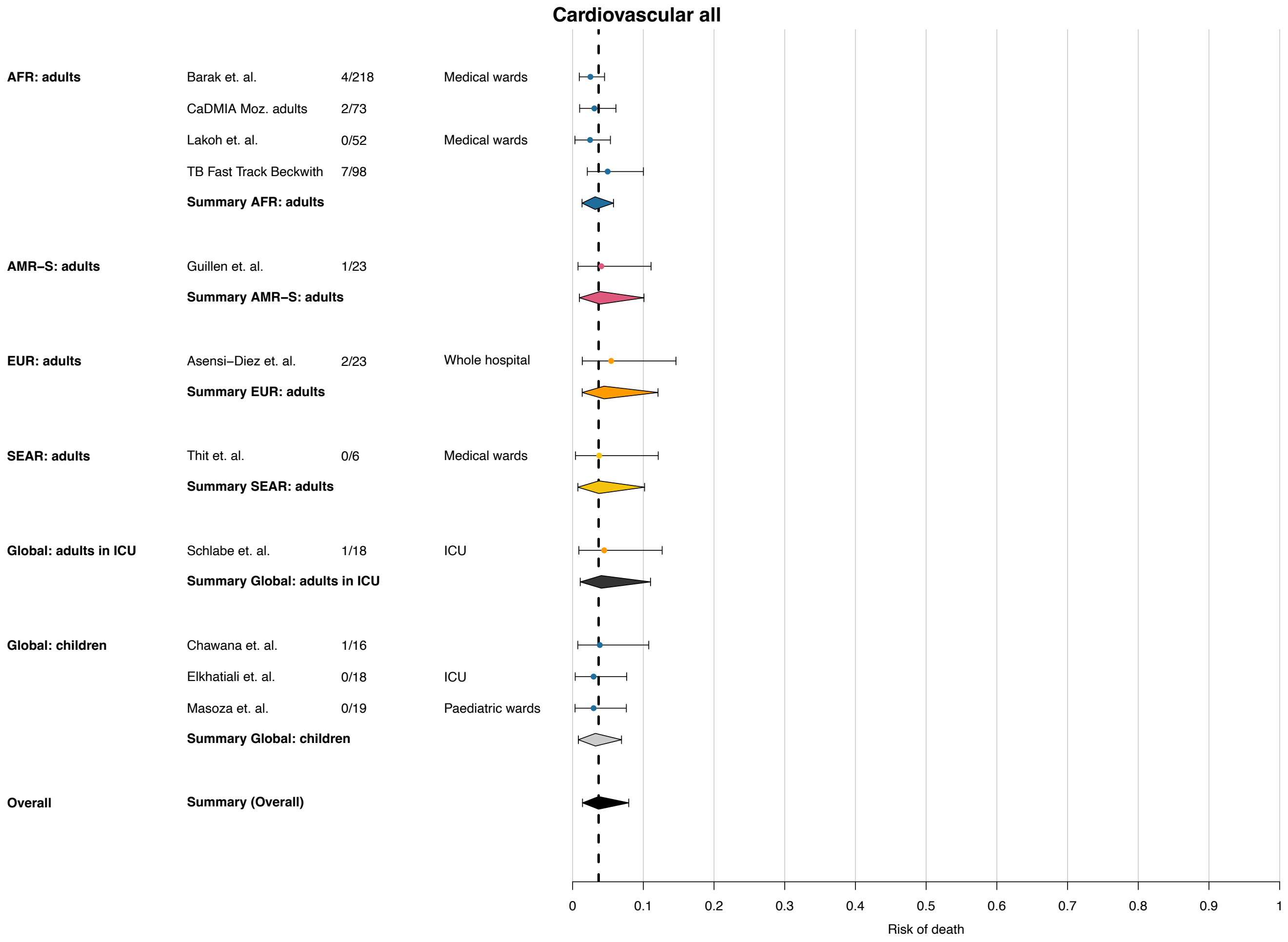

S Figure 23: Forest plot for estimate of proportion of deaths due to haematological disease (all)

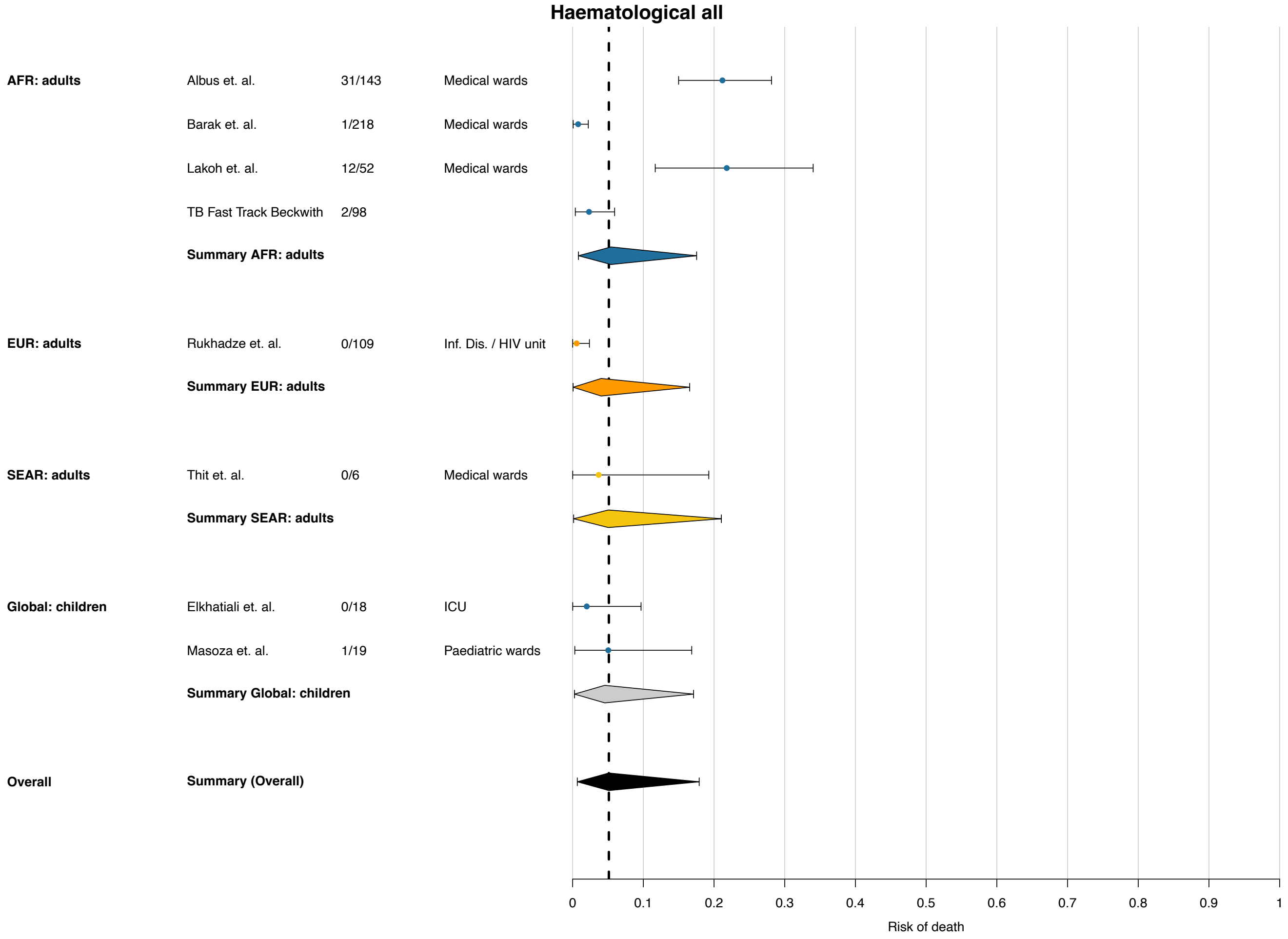

S Figure 24: Forest plot for estimate of proportion of deaths due to Liver Disease (all)

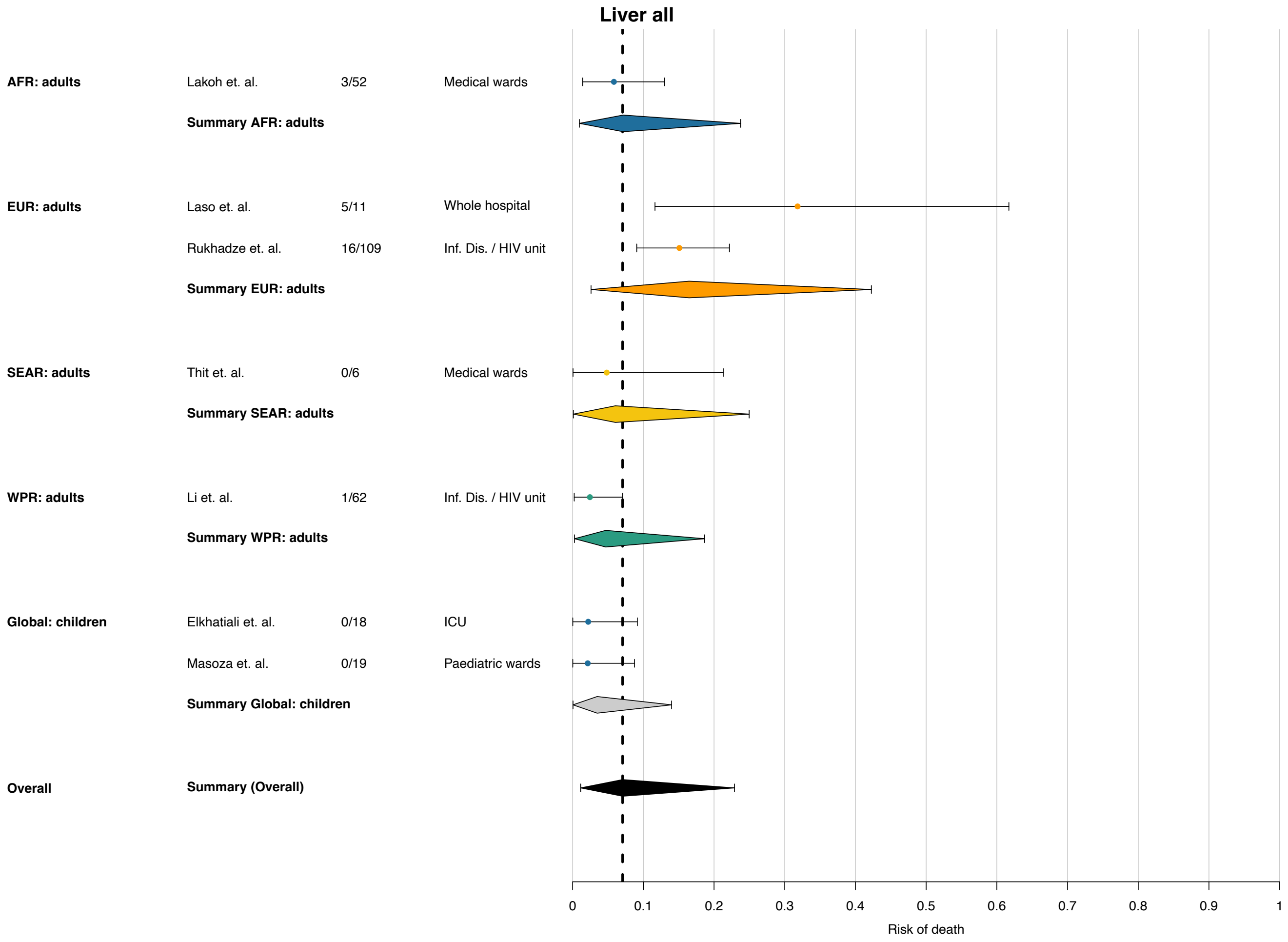

Section and Topic	Item #	Checklist item	Location where item is reported
**TITLE**			
Title	1	Identify the report as a systematic review.	Title
**ABSTRACT**			
Abstract	2	See the PRISMA 2020 for Abstracts checklist.	NA
**INTRODUCTION**			
Rationale	3	Describe the rationale for the review in the context of existing knowledge.	p. 3
Objectives	4	Provide an explicit statement of the objective(s) or question(s) the review addresses.	p. 3
**METHODS**			
Eligibility criteria	5	Specify the inclusion and exclusion criteria for the review and how studies were grouped for the syntheses.	p.3 / 4 (and appendix)
Information sources	6	Specify all databases, registers, websites, organisations, reference lists and other sources searched or consulted to identify studies. Specify the date when each source was last searched or consulted.	p.3 / 4 (and appendix)
Search strategy	7	Present the full search strategies for all databases, registers and websites, including any filters and limits used.	Appendix to TLHIV paper (reference #9)
Selection process	8	Specify the methods used to decide whether a study met the inclusion criteria of the review, including how many reviewers screened each record and each report retrieved, whether they worked independently, and if applicable, details of automation tools used in the process.	p.4 and PRISMA diagram
Data collection process	9	Specify the methods used to collect data from reports, including how many reviewers collected data from each report, whether they worked independently, any processes for obtaining or confirming data from study investigators, and if applicable, details of automation tools used in the process.	p. 4, appendix and appendix to TLHIV paper (reference #9)
Data items	10a	List and define all outcomes for which data were sought. Specify whether all results that were compatible with each outcome domain in each study were sought (e.g. for all measures, time points, analyses), and if not, the methods used to decide which results to collect.	p. 4, appendix and appendix to TLHIV paper (reference #9)
	10b	List and define all other variables for which data were sought (e.g. participant and intervention characteristics, funding sources). Describe any assumptions made about any missing or unclear information.	p. 4, appendix and appendix to TLHIV paper (reference #9)
Study risk of bias assessment	11	Specify the methods used to assess risk of bias in the included studies, including details of the tool(s) used, how many reviewers assessed each study and whether they worked independently, and if applicable, details of automation tools used in the process.	p. 4, appendix #9)
Effect measures	12	Specify for each outcome the effect measure(s) (e.g. risk ratio, mean difference) used in the synthesis or presentation of results.	p.5
Synthesis methods	13a	Describe the processes used to decide which studies were eligible for each synthesis (e.g. tabulating the study intervention characteristics and comparing against the planned groups for each synthesis (item #5)).	p.5
	13b	Describe any methods required to prepare the data for presentation or synthesis, such as handling of missing summary statistics, or data conversions.	NA
	13c	Describe any methods used to tabulate or visually display results of individual studies and syntheses.	NA – although this is apparent in results
	13d	Describe any methods used to synthesize results and provide a rationale for the choice(s). If meta-analysis was performed, describe the model(s), method(s) to identify the presence and extent of statistical heterogeneity, and software package(s) used.	p. 5 and appendix
	13e	Describe any methods used to explore possible causes of heterogeneity among study results (e.g. subgroup analysis,	p. 5 and appendix

Section and Topic	Item #	Checklist item	Location where item is reported
		meta-regression).	
	13f	Describe any sensitivity analyses conducted to assess robustness of the synthesized results.	Appendix
Reporting bias assessment	14	Describe any methods used to assess risk of bias due to missing results in a synthesis (arising from reporting biases).	NA
Certainty assessment	15	Describe any methods used to assess certainty (or confidence) in the body of evidence for an outcome.	Picked up in discussion
**RESULTS**			
Study selection	16a	Describe the results of the search and selection process, from the number of records identified in the search to the number of studies included in the review, ideally using a flow diagram.	Fig 1 and p. 6
	16b	Cite studies that might appear to meet the inclusion criteria, but which were excluded, and explain why they were excluded.	Appendix
Study characteristics	17	Cite each included study and present its characteristics.	Appendix
Risk of bias in studies	18	Present assessments of risk of bias for each included study.	Appendix
Results of individual studies	19	For all outcomes, present, for each study: (a) summary statistics for each group (where appropriate) and (b) an effect estimate and its precision (e.g. confidence/credible interval), ideally using structured tables or plots.	Appendix, and Fig 2 / 3 / 4
Results of syntheses	20a	For each synthesis, briefly summarise the characteristics and risk of bias among contributing studies.	Appendix
	20b	Present results of all statistical syntheses conducted. If meta-analysis was done, present for each the summary estimate and its precision (e.g. confidence/credible interval) and measures of statistical heterogeneity. If comparing groups, describe the direction of the effect.	Appendix, and Fig 2 / 3 / 4
	20c	Present results of all investigations of possible causes of heterogeneity among study results.	Heterogeneity by region and type of ward is discussed throughout
	20d	Present results of all sensitivity analyses conducted to assess the robustness of the synthesized results.	Appendix (autopsy studies and low ROB only), also p.10
Reporting biases	21	Present assessments of risk of bias due to missing results (arising from reporting biases) for each synthesis assessed.	NA
Certainty of evidence	22	Present assessments of certainty (or confidence) in the body of evidence for each outcome assessed.	All estimates have confidence intervals, see discussion for more detail.
**DISCUSSION**			
Discussion	23a	Provide a general interpretation of the results in the context of other evidence.	p.11
	23b	Discuss any limitations of the evidence included in the review.	p.12
	23c	Discuss any limitations of the review processes used.	p.12
	23d	Discuss implications of the results for practice, policy, and future research.	p.13
**OTHER INFORMATION**			
Registration and protocol	24a	Provide registration information for the review, including register name and registration number, or state that the review was not registered.	NA – not pre-registered

Section and Topic	Item #	Checklist item	Location where item is reported
	24b	Indicate where the review protocol can be accessed, or state that a protocol was not prepared.	Reference #9
	24c	Describe and explain any amendments to information provided at registration or in the protocol.	NA
Support	25	Describe sources of financial or non-financial support for the review, and the role of the funders or sponsors in the review.	Declarations
Competing interests	26	Declare any competing interests of review authors.	Declarations
Availability of data, code and other materials	27	Report which of the following are publicly available and where they can be found: template data collection forms; data extracted from included studies; data used for all analyses; analytic code; any other materials used in the review.	Declarations – some data already available at a LSHTM repo and rest will be uploaded.

S Table 10: Records considered at full text and reasons for exclusion

Author	Title	Journal	Reasons for exclusion
Abati et al.	HIV testing and clinical status upon admission to a specialized health care unit in Para, Brazil	Revista de saude publica	e) Not hospitalised
Abayneh et al.	Acute respiratory infections (ARIs) and factors associated with their poor clinical outcome among children under-five years attending pediatric wards of public hospital in Southwest district of Ethiopia: A prospective observational cohort study	European Journal of Inflammation	c) Not all PLHIV
Adakun et al.	Validation of a tuberculous meningitis case definition in Mbarara regional referral hospital, Uganda	American Journal of Tropical Medicine and Hygiene	h) All have a syndrome
Adegoke et al.	Mortality pattern and causes at the adult medical unit of the Accident and Emergency Department of a tertiary health care facility in Lagos, Nigeria	Nigerian Quarterly Journal of Hospital Medicine	g) No diagnoses
Adjei et al.	Unrecognized human immunodeficiency virus infection and risk factors among elderly medical patients at the Korle Bu teaching hospital, Accra, Ghana	Tropical diseases, travel medicine and vaccines	g) No diagnoses
Agazhe et al.	Prevalence of anaemia and its associated factors among HIV-infected adults at the time of ART initiation at Debre Markos Comprehensive Specialized Hospital, Northwest Ethiopia: a retrospective cross-sectional study	BMJ Open	e) Not hospitalised
Agudelo et al.	Outcomes and complications of hospitalised patients with HIV-TB co-infection	Tropical medicine & international health : TM & IH	f) Everyone has same diagnosis
Agudelo-Gonzalez et al.	Opportunistic infections in patients with HIV in Neiva's University Hospital, Colombia. 2007-2012	Infectio	i) Pre 2014
Ahmed et al.	Prospective validation of the universal vital assessment (UVA) score to predict the in-hospital mortality of patients with acute illness admitted to a government district hospital in KwaZulu-Natal, South Africa	Open Forum Infectious Diseases	g) No diagnoses
Akech et al.	Tackling post-discharge mortality in children living in LMICs to reduce child deaths	The Lancet Child and Adolescent Health	c) Not all PLHIV
Akgun et al.	Hospital readmission and incident skilled nursing facility placement in micu survivors from a nationally representative cohort of HIV-infected and uninfected veterans: 1996-2010	American Journal of Respiratory and Critical Care Medicine	g) No diagnoses
Akgun et al.	An adapted frailty-related phenotype and the VACS index as predictors of hospitalization and mortality in HIV-infected and uninfected individuals	Journal of acquired immune deficiency syndromes (1999)	g) No diagnoses
Akgun et al.	Delirium among people aging with and without HIV: Role of alcohol and Neurocognitively active medications	Journal of the American Geriatrics Society	g) No diagnoses
Akodjenou et al.	Death of infants born to HIV-positive mothers admitted to intensive care at the University Hospital Center of Abomey-Calavi (CHUZ-AC)	Deces des nourrissons nes des meres seropositives admis aux soins intensifs du Centre Hospitalier Universitaire de Zone d'Abomey-Calavi (CHUZ-AC).	l) Cause of admission only
Akor et al.	Infectious diseases co-morbidities among patients attending Kogi State University Teaching Hospital: A ten-year retrospective study	African Journal of Clinical and Experimental Microbiology	i) Pre 2014
Al Shamsi et al.	Patterns of Pulmonary manifestations of HIV/AIDS among patients admitted to Rashid Hospital, Dubai; 2015-2017	Journal of Infection and Public Health	l) Cause of admission only
Alaoui et al.	Tuberculosis and HIV Coinfection: A Review of 135 Cases Experience of the Infectious Diseases Department- CHU Mohamed VI- Marrakech	Open Forum Infectious Diseases	f) Everyone has same diagnosis
Albus et al.	Poor outcomes among critically ill HIV-positive patients at hospital discharge and post-discharge in Guinea, Conakry: A retrospective cohort study	PloS one	INCLUDED
Alemu et al.	Utilization of Healthcare Resources by HIV-Positive Children in the United States: A National Perspective	Southern medical journal	i) Pre 2014

Ali et al.	Evaluation of an HIV-Related Mortuary Surveillance System - Nairobi, Kenya, Two Sites, 2015	Morbidity and mortality weekly report. Surveillance summaries (Washington, D.C. : 2002)	g) No diagnoses
Allain et al.	127 A DESCRIPTIVE STUDY OF PATTERNS OF DISEASE AND CLINICAL OUTCOMES IN OLDER ADULTS ADMITTED TO MEDICAL WARDS IN A CENTRAL HOSPITAL IN MALAWI	Age & Ageing	c) Not all PLHIV
Allain et al.	Age Related Patterns of Disease and Mortality in Hospitalised Adults in Malawi	PloS one	g) No diagnoses
Almirol et al.	Hospitalization and emergency room visits: The opportunity for re-engagement for people living with HIV/AIDs (PLWHA)	Open Forum Infectious Diseases	g) No diagnoses
Aluisio et al.	Assessment of Standard HIV Testing Services Delivery to Injured Persons Seeking Emergency Care in Nairobi, Kenya: A Prospective Observational Study	medRxiv	h) All have a syndrome
Alvarez Barreneche et al.	Hospitalization causes and outcomes in HIV patients in the late antiretroviral era in Colombia	AIDS research and therapy	INCLUDED
Alvarez et al.	Epidemiologic and clinical profile of hospitalized HIV Colombian patients	Open Forum Infectious Diseases	g) No diagnoses
Andama et al.	The transition to Xpert MTB/RIF ultra: diagnostic accuracy for pulmonary tuberculosis in Kampala, Uganda	BMC infectious diseases	a) Irrelevant
Anderson et al.	Hospitalization among infants who initiate antiretroviral therapy before 3 months of age	AIDS (London, England)	k) Duplicate record
Andrade et al.	Short- and medium-term prognosis of HIV-infected patients receiving intensive care: a Brazilian multicentre prospective cohort study	HIV medicine	INCLUDED
Andrade et al.	Short,Äê and medium,Äêterm prognosis of HIV,Äêinfected patients receiving intensive care: a Brazilian multicentre prospective cohort study	HIV Medicine	l) Cause of admission only
Andrade et al.	Central nervous system infection in the intensive care unit: Development and validation of a multi-parameter diagnostic prediction tool to identify suspected patients	PloS one	c) Not all PLHIV
Andreia et al.	Mortality predictive factors of People Living with Human-immunodeficiency-virus and Bloodstream Infection	International journal of infectious diseases : IJID : official publication of the International Society for Infectious Diseases	i) Pre 2014
Andreu-Crespo et al.	Hospital admissions due to medical conditions in a public health care system with free access to antiretroviral treatment	Journal of the International AIDS Society	l) Cause of admission only
Andrews et al.	Simplified severe sepsis protocol: a randomized controlled trial of modified early goal-directed therapy in Zambia	Critical care medicine	h) All have a syndrome
Anonymous	Guidelines for HIV Mortality Measurement	NA	a) Irrelevant
Antinori et al.	Fungal meningitis in England and Wales	The Lancet Infectious Diseases	a) Irrelevant
Antoine et al.	HIV-infected patients hospitalized in intensive care from 1997 to 2020: analysis of the OUTCOMEREA multicenter cohort	Annals of Intensive Care	INCLUDED
Antoniou et al.	Comparison of comorbidity classification methods for predicting outcomes in a population-based cohort of adults with human immunodeficiency virus infection	Annals of epidemiology	g) No diagnoses
Arodiwe et al.	Medical causes of death in a teaching hospital in South-Eastern Nigeria: A 16 year review	Nigerian journal of clinical practice	g) No diagnoses
Asensi-Diez et al.	[Hospital admission and mortality causes of HIV patients in a third level hospital]	Diagnostico al alta y causas de mortalidad de pacientes VIH+ ingresados en un hospital de tercer nivel.	INCLUDED

Ashatha et al.	Prevelance of Central Nervous System Cryptococcosis in Human Immunodeficiency Virus Reactive Hospitalised Patients	International Journal of Pharmaceutical and Clinical Research	a) Irrelevant
Asiimwe et al.	Frequency of vital signs monitoring and its association with mortality among adults with severe sepsis admitted to a general medical ward in Uganda	PloS one	h) All have a syndrome
Asmarawati et al.	Opportunistic infection manifestation of HIV-AIDS patients in Airlangga university hospital Surabaya	1st International Conference on Tropical Medicine and Infectious Diseases (ICTROMI)	l) Cause of admission only
Assefa et al.	Missed pulmonary tuberculosis: a cross sectional study in the general medical inpatient wards of a large referral hospital in Ethiopia	BMC infectious diseases	c) Not all PLHIV
Assy et al.	High prevalence of Cryptococcal antigenemia using a finger-prick lateral flow assay in individuals with advanced HIV disease in Santarem Municipality, Brazilian Amazon Basin	Medical mycology	INCLUDED
Aston et al.	Etiology and Risk Factors for Mortality in an Adult Community-acquired Pneumonia Cohort in Malawi	American journal of respiratory and critical care medicine	h) All have a syndrome
Avina et al.	Burden of Mental Illness among Primary HIV Discharges: A Retrospective Analysis of Inpatient Data	Healthcare (Basel, Switzerland)	g) No diagnoses
Avoundjian et al.	Evaluation of an Emergency Department and Hospital-Based Data Exchange to Improve HIV Care Engagement and Viral Suppression	Sexually transmitted diseases	g) No diagnoses
Aye et al.	Pattern and outcome of opportunistic infections in hospitalized hiv-infected patients in specialist hospital waibargi, Myanmar	Journal of the Pediatric Infectious Diseases Society	z) Duplicate paper in same study
Ayieko et al.	Characteristics of admissions and variations in the use of basic investigations, treatments and outcomes in Kenyan hospitals within a new Clinical Information Network	Archives of disease in childhood	c) Not all PLHIV
Aylward et al.	Risk factors and outcomes of acute kidney injury in South African critically ill adults: a prospective cohort study	BMC nephrology	g) No diagnoses
Azovtseva et al.	Cerebral toxoplasmosis in HIV-infected patients over 2015-2018 (a case study of Russia)	Epidemiology and infection	f) Everyone has same diagnosis
Bachhuber et al.	Hospitalization rates of people living with HIV in the United States, 2009	Public health reports (Washington, D.C. : 1974)	i) Pre 2014
Baesi et al.	Antiretroviral drug resistance among antiretroviral-naive and treatment experienced patients infected with HIV in Iran	Journal of medical virology	e) Not hospitalised
Bahceci et al.	Eight Year Evaluation of HIV/Tuberculosis co-infection in Patients Admitted to Our Hospital	Eastern Journal of Medicine	h) All have a syndrome
Bai et al.	Non-AIDS bacterial infections are the main cause of hospital admissions in HIV-infected patients over 2010-2017: Data from the San Paolo Infectious Diseases (SPID) cohort	Journal of the International AIDS Society	i) Pre 2014
Baik et al.	Undetected tuberculosis at enrollment and after hospitalization in medical and oncology wards in Botswana	PloS one	l) Cause of admission only
Baker et al.	Vital Signs Directed Therapy: Improving Care in an Intensive Care Unit in a Low-Income Country	PloS one	c) Not all PLHIV
Baker et al.	HIV inpatient admissions and antiretroviral treatment interventions	HIV Medicine	INCLUDED
Bakewell et al.	Estimating the risk of mortality attributable to late HIV diagnosis following admission to the intensive care unit: a single-centre observational cohort study	Journal of the International Aids Society	i) Pre 2014
Balakrishna et al.	Decreasing Incidence and Determinants of Bacterial Pneumonia in People With HIV: The Swiss HIV Cohort Study	The Journal of infectious diseases	f) Everyone has same diagnosis
Balakrishnan et al.	Infectious Disease Complications in Hospitalized Patients with Opioid Use Disorder at a Southern County Hospital	Open Forum Infectious Diseases	c) Not all PLHIV

Ballot et al.	Retrospective cross-sectional review of survival rates in critically ill children admitted to a combined paediatric/neonatal intensive care unit in Johannesburg, South Africa, 2013-2015	BMJ open	c) Not all PLHIV
Banda et al.	Prevalence and Factors Associated with Renal Dysfunction in HIV Positive and Negative Adults at the University Teaching Hospital, in Lusaka	Medical Journal of Zambia	g) No diagnoses
Banda et al.	Common impairments and functional limitations of HIV sequelae that require physiotherapy rehabilitation in the medical wards at Queen Elizabeth Central Hospital, Malawi: A cross sectional study	Malawi medical journal : the journal of Medical Association of Malawi	g) No diagnoses
Banda et al.	MON-337 PREDICTORS OF ACUTE KIDNEY INJURY AND MORTALITY IN THE INTENSIVE CARE UNIT AT A TEACHING TERTIARY HOSPITAL	Kidney International Reports	h) All have a syndrome
Banerdt et al.	Risk factors for delirium among hospitalized patients in Zambia	PloS one	c) Not all PLHIV
Barak et al.	HIV-associated morbidity and mortality in a setting of high ART coverage: prospective surveillance results from a district hospital in Botswana	Journal of the International AIDS Society	INCLUDED
Barbier et al.	Temporal trends in critical events complicating HIV infection: 1999-2010 multicentre cohort study in France	Intensive care medicine	i) Pre 2014
Basso et al.	Disseminated Histoplasmosis in Persons with HIV/AIDS, Southern Brazil, 2010-2019	Emerging infectious diseases	f) Everyone has same diagnosis
Bates et al.	Burden of respiratory tract infections at post mortem in Zambian children	BMC medicine	i) Pre 2014
Batra et al.	Impact of an electronic medical record on the incidence of antiretroviral prescription errors and HIV pharmacist reconciliation on error correction among hospitalized HIV-infected patients	Antiviral therapy	g) No diagnoses
Beck et al.	SAT-165 THE INCIDENCE AND SEVERITY OF COMMUNITY-ACQUIRED ACUTE KIDNEY INJURY AMONGST UNSELECTED MEDICAL ADMISSIONS IN A RURAL DISTRICT HOSPITAL IN SIERRA LEONE	Kidney International Reports	g) No diagnoses
Beckwith et al.	Causes and Outcomes of Admission and Investigation of Tuberculosis in Adults with Advanced HIV in South African Hospitals: Data from the TB Fast Track Trial	The American journal of tropical medicine and hygiene	e) Not hospitalised
Bell et al.	The indirect effects of COVID-19 on the morbidity and mortality of people living with HIV	HIV Medicine	z) Duplicate paper in same study
Bellino et al.	Trends of hospitalisations rates in a cohort of HIV-infected persons followed in an Italian hospital from 1998 to 2016	Epidemiology and infection	z) Duplicate paper in same study
Benites et al.	Hospitalizav√ßv£o de adultos com Aids em unidade de terapia intensiva: estudo anal√≠tico	Online braz. j. nurs. (Online)	l) Cause of admission only
Bennett et al.	Multicenter Study of Outcomes Among Persons With HIV Who Presented to US Emergency Departments With Suspected SARS-CoV-2	Journal of acquired immune deficiency syndromes (1999)	h) All have a syndrome
Bentley et al.	CHARACTERISING ADMISSIONS TO A SPECIALIST HIV INPATIENT CENTRE: DEMOGRAPHICS, DIAGNOSIS AND IDEAS FOR SERVICE DEVELOPMENT	Sexually Transmitted Infections	l) Cause of admission only
Berry et al.	Trends in hospitalizations among children and young adults with perinatally acquired HIV	The Pediatric infectious disease journal	i) Pre 2014
Bertagnolio et al.	Clinical features of, and risk factors for, severe or fatal COVID-19 among people living with HIV admitted to hospital: analysis of data from the WHO Global Clinical Platform of COVID-19	The lancet. HIV	f) Everyone has same diagnosis
Besse Dv≠az et al.	Aspectos cl√≠nicos y epidemiol√≥gicos en pacientes con s√≠ndrome de inmunodeficiencia adquirida de la provincia de Santiago de Cuba	Medisan	INCLUDED
Bielick et al.	National and Regional Rates of Hospitalizations and In-Hospital Mortality for Opportunistic Infections for People with HIV in the United States, 2012-2018	Journal of the International Aids Society	h) All have a syndrome
Biglia et al.	Prognosis of patients with hiv infection upon admission to an intensive care unit	International Journal of Infectious Diseases	i) Pre 2014
Birajdar et al.	Study of correlation between clinical profile, CD4 count and total lymphocyte count in HIV infected patients at rural tertiary care institute	NA	g) No diagnoses

Bishnu et al.	Assessment of clinico-immunological profile of newly diagnosed HIV patients presenting to a teaching hospital of Eastern India	Indian Journal of Medical Research	e) Not hospitalised
Bishop et al.	Prognostic value of the Quick Sepsis-related Organ Failure Assessment (qSOFA) score among critically ill medical and surgical patients with suspected infection in a resource-limited setting	African journal of thoracic and critical care medicine	h) All have a syndrome
Bisnauth et al.	Improving antiretroviral therapy initiation in hospital and after discharge in Johannesburg, South Africa	Journal of the International AIDS Society	g) No diagnoses
Bizune et al.	Treatment Complexities Among Patients with Tuberculosis in a High HIV Prevalence Cohort in the United States	AIDS research and human retroviruses	f) Everyone has same diagnosis
Bjerrum et al.	Diagnostic accuracy of the rapid urine lipoarabinomannan test for pulmonary tuberculosis among HIV-infected adults in Ghana-findings from the DETECT HIV-TB study	BMC infectious diseases	i) Pre 2014
Blan et al.	Biomarker detection for the diagnosis of disseminated histoplasmosis in people living with HIV/ AIDS in Southern Brazil: A year of implementation	Medical Mycology	INCLUDED
Blanc et al.	Severe bacterial non-AIDS infections in persons with HIV: the epidemiology and evolution of antibiotic resistance over an 18-year period (2000-2017) in the ANRS CO3 AquiVih-Nouvelle-Aquitaine cohort	Clinical infectious diseases : an official publication of the Infectious Diseases Society of America	f) Everyone has same diagnosis
Bock et al.	Acute care - an important component of the continuum of care for HIV and tuberculosis in developing countries	Anaesthesia	d) Not primary research
Bogoch et al.	Identifying HIV care continuum gaps with verbal autopsy	The lancet. HIV	d) Not primary research
Bogoni et al.	Cytomegalovirus retinitis in hospitalized people living with HIV in the late antiretroviral therapy era in Sao Paulo, Brazil	International journal of STD & AIDS	h) All have a syndrome
Boniatti et al.	Early antiretroviral therapy for HIV-infected patients admitted to an intensive care unit (EARTH-ICU): A randomized clinical trial	PloS one	z) Duplicate paper in same study
Bonnet et al.	Burden of comorbidities in hospitalized HIV patients in France: National French Medical Information System Database, 2014	Antiviral Therapy	i) Pre 2014
Borges et al.	PREVALENCIA DA ANTIGENEMIA CRIPTOCOCICA UTILIZANDO LATERAL FLOW ASSAY (LFA) EM PACIENTES COM HIV/AIDS SINTOMATICOS TRIADOS EM UNIDADE DE REFERENCIA EM GOIAS	Brazilian Journal of Infectious Diseases	l) Cause of admission only
Bornstein et al.	Point of care screening for cryptococcal disease among hospitalized HIV infected adults in ethiopia	Journal of Investigative Medicine	f) Everyone has same diagnosis
Bottieau et al.	Clinical spectrum, main etiologies and outcome of neurological disorders in the rural Hospital of Mosango, Province of Bandundu, Democratic Republic of the Congo	Tropical Medicine and International Health	c) Not all PLHIV
Boubouchairopoulou et al.	Estimation of the Direct Cost of HIV-Infected Patients in Greece on an Annual Basis	Value in health regional issues	e) Not hospitalised
Bouee et al.	Impact of Comorbidities on the Over-Mortality of People Living With Human Immunodeficiency Viruses (PLHIV)	Value in Health	e) Not hospitalised
Brandao et al.	Community-acquired pneumonia requiring hospitalization in HIV-infected patients	European Respiratory Journal	f) Everyone has same diagnosis
Brink et al.	A network approach to ensure high-quality HIV outcomes: The experience of a remote small unit	HIV Medicine	e) Not hospitalised
Brits et al.	Child deaths at national district hospital, free state: One a month is better than one a week	South African Family Practice	i) Pre 2014
Brizzi et al.	Effects of a pharmacist-driven antiretroviral stewardship and transitions of care service in persons living with HIV/AIDS	Open Forum Infectious Diseases	g) No diagnoses
Broli et al.	Aspectos de interv©s cl√≠nico epidemiol√≥gico en las infecciones respiratorias de pacientes VIH/SIDA	Archivos de Medicina Interna	h) All have a syndrome

Brotherton et al.	Between an auroc and a hard place: Comparison of three mortality scores and one regression model in a resource limited ICU in Kenya	American Journal of Respiratory and Critical Care Medicine	c) Not all PLHIV
Brown et al.	Risk factors for 30-day readmission among patients with human immunodeficiency virus infection in an urban teaching hospital	Journal of the American Pharmacists Association	i) Pre 2014
Bunn et al.	Evaluation of human immunodeficiency virus medication errors in a community hospital following the implementation of a pharmacist-led antiretroviral stewardship program	JACCP Journal of the American College of Clinical Pharmacy	z) Duplicate paper in same study
Burke et al.	Enhanced tuberculosis screening using computer-aided X-ray diagnosis and novel point of care urine lipoarabinomannan assay among adults with HIV admitted to hospital (CASTLE study): a cluster randomised trial	Journal of the International Aids Society	l) Cause of admission only
Burns et al.	Slipping through the cracks: a qualitative study to explore pathways of HIV care and treatment amongst hospitalised patients with advanced HIV in Kenya and the Democratic Republic of the Congo	AIDS care	g) No diagnoses
Buss et al.	Validating a novel index (SWAT-Bp) to predict mortality risk of community-acquired pneumonia in Malawi	Malawi medical journal : the journal of Medical Association of Malawi	h) All have a syndrome
Bwakura-Dangarembizi et al.	A randomized trial of prolonged co-trimoxazole in HIV-infected children in Africa	The New England journal of medicine	e) Not hospitalised
Byass et al.	InterVA-4 as a public health tool for measuring HIV/AIDS mortality: a validation study from five African countries	Global Health Action	i) Pre 2014
Byass et al.	Special Issue: INDEPTH network cause-specific mortality	Special Issue: INDEPTH network cause-specific mortality.	d) Not primary research
Byass et al.	Comparing verbal autopsy cause of death findings as determined by physician coding and probabilistic modelling: a public health analysis of 54 000 deaths in Africa and Asia	Journal of global health	i) Pre 2014
Cammarota et al.	Impact of comorbidity on the risk and cost of hospitalization in HIV-infected patients: real-world data from Abruzzo Region	ClinicoEconomics and outcomes research : CEOR	i) Pre 2014
Camon et al.	Reason for HIV patients consultation to the emergency department in the HAART era: incidence and mortality	Journal of AIDS and Clinical Research	i) Pre 2014
Campbell et al.	Inpatient initiation of art improves short-term mortality in people living with HIV	Open Forum Infectious Diseases	g) No diagnoses
Cao et al.	Prevalence of anaemia and the associated factors among hospitalised people living with HIV receiving antiretroviral therapy in Southwest China: a cross-sectional study	BMJ open	z) Duplicate paper in same study
Carey et al.	Prospective cohort study to identify prevalence, risk factors and outcomes of infection associated kidney disease in a regional hospital in Malawi	BMJ open	g) No diagnoses
Caro-Vega et al.	Characteristics of PLWH requiring hospitalization at tertiary healthcare institutions during COVID-10 pandemic in Mexico City	HIV Medicine	g) No diagnoses
Casado et al.	Low risk of bacterial co-infection, opportunistic diseases, and persistent immunosuppression in people living with HIV and COVID-19	Infection	f) Everyone has same diagnosis
Castillo et al.	Validity of a Minimally Invasive Autopsy for Cause of Death Determination in Adults in Mozambique: An Observational Study	PLoS Medicine	INCLUDED
Castro-Lima et al.	Impact of human immunodeficiency virus infection on mortality of patients who acquired healthcare associated-infection in critical care unit	Medicine	c) Not all PLHIV
Catumbela et al.	HIV disease burden, cost, and length of stay in Portuguese hospitals from 2000 to 2010: a cross-sectional study	BMC health services research	i) Pre 2014
Cavalcante et al.	Death-related factors in HIV/AIDS patients undergoing hemodialysis in an intensive care unit	Revista do Instituto de Medicina Tropical de Sao Paulo	g) No diagnoses

Cenderello et al.	Inpatient admissions of patients living with HIV in two European centres (UK and Italy); comparisons and contrasts	The Journal of infection	i) Pre 2014
Cerme√±o et al.	Infecciones f√ngicas en pacientes infectados por VIH en el Complejo Hospitalario Universitario "Ruiz y P√°ez"	Bol. venez. infectol	h) All have a syndrome
Chaka et al.	Presentation and outcome of suspected sepsis in a high-HIV burden, high antiretroviral coverage setting	International Journal of Infectious Diseases	h) All have a syndrome
Challe et al.	Pattern of all-causes and cause-specific mortality in an area with progressively declining malaria burden in Korogwe district, north-eastern Tanzania	Malaria journal	e) Not hospitalised
Chan-Carusone et al.	The lived experience of the hospital discharge 'plan': A case study of adults living with HIV/AIDS and complex lives	Canadian Journal of Infectious Diseases and Medical Microbiology	h) All have a syndrome
Chang et al.	Brief Report: COVID-19 Testing, Characteristics, and Outcomes Among People Living With HIV in an Integrated Health System	Journal of acquired immune deficiency syndromes (1999)	f) Everyone has same diagnosis
Channabasappa et al.	A prospective study of hiv positive autopsies conducted at aniims, port blair, andaman & nicobar islands	Indian Journal of Forensic Medicine and Toxicology	INCLUDED
Chaudhary et al.	A Study of Cardiovascular Abnormalities in HIV Positive Patients in a Tertiary Care Hospital in Northern India	The Journal of the Association of Physicians of India	i) Pre 2014
Chawana et al.	Potential of Minimally Invasive Tissue Sampling for Attributing Specific Causes of Childhood Deaths in South Africa: A Pilot, Epidemiological Study	Clinical infectious diseases : an official publication of the Infectious Diseases Society of America	INCLUDED
Chawla et al.	Policy to practice: impact of GeneXpert MTB/RIF implementation on the TB spectrum of care in Lilongwe, Malawi	Transactions of the Royal Society of Tropical Medicine and Hygiene	h) All have a syndrome
Chen et al.	Serum cryptococcal antigen titre as a diagnostic tool and a predictor of mortality in HIV-infected patients with cryptococcal meningitis	HIV medicine	z) Duplicate paper in same study
Chen et al.	Clinical characteristics of patients with acquired immunodeficiency syndrome having respiratory symptoms as the initial manifestations: A retrospective study	European Journal of Inflammation	h) All have a syndrome
Chenciner et al.	Prognostic relevance of lymphocyte-CRP ratio and CRP-albumin ratio as markers of inflammation in hospitalised adults with HIV	Hiv Medicine	g) No diagnoses
Chewe et al.	Tuberculosis Mortalities Among In-patients at a Tertiary Hospital in Zambia Between 2018 and 2019 - The Spectrum of Clinical Presentations	medRxiv	f) Everyone has same diagnosis
Chiampas et al.	Evaluation of the occurrence and type of antiretroviral and opportunistic infection medication errors within the inpatient setting	Pharmacy practice	i) Pre 2014
Chihota et al.	Noncommunicable diseases as reasons for admission among hivinfected adults in Zambia	Topics in Antiviral Medicine	h) All have a syndrome
Chow et al.	Hospitalization rates among persons with HIV who gained medicaid or private insurance in 2014	Open Forum Infectious Diseases	l) Cause of admission only
Chow et al.	Brief Report: Hospitalization Rates Among Persons With HIV Who Gained Medicaid or Private Insurance After the Affordable Care Act in 2014	Journal of acquired immune deficiency syndromes (1999)	l) Cause of admission only
Chow et al.	Hospitalization rates among persons with HIV who gained Medicaid or private insurance after the Affordable Care Act in 2014	JAIDS, Journal of Acquired Immune Deficiency Syndromes	INCLUDED
Chuaychoo et al.	Clinical manifestations and outcomes of respiratory syncytial virus infection in adult hospitalized patients	Journal of Clinical Virology	f) Everyone has same diagnosis
Cichowitz et al.	Hospitalization and post-discharge care in South Africa: A critical event in the continuum of care	PloS one	l) Cause of admission only
Cidral et al.	Assessment of the risk of pressure ulcer development among hospitalized HIV/Aids patients	Revista brasileira de enfermagem	i) Pre 2014

Claire Ndayisaba et al.	CAUSE of Death Assignment Using Minimally Invasive Tissue Sampling in Low Resource Settings: A Cross-Sectional Study from The University Teaching Hospital of Kigali, Rwanda	Modern Pathology	g) No diagnoses
Claudia et al.	The spectrum of infectious diseases hospital mortality by HIV status	Acta Medica Mediterranea	i) Pre 2014
Cobaschi et al.	INTERDISCIPLINARY IN MANAGING HIV/AIDS INFECTED PATIENTS-THE IMPORTANCE OF TEAMWORK	Medical-Surgical Journal-Revista Medico-Chirurgicala	INCLUDED
Cobos-Trigueros et al.	Acquisition of resistant microorganisms and infections in HIV-infected patients admitted to the ICU	European journal of clinical microbiology & infectious diseases : official publication of the European Society of Clinical Microbiology	i) Pre 2014
Coelho et al.	Fatores associados ao risco de interna√ß√£o dos indiv√≠duos HIV(+)	NA	i) Pre 2014
Cohen et al.	Lost but not forgotten: A population‚Äêbased study of mortality and care trajectories among people living with HIV who are lost to follow‚Äêup in Ontario, Canada	HIV Medicine	g) No diagnoses
Coker et al.	An Evaluation of Renal Care received by Human Immunodeficiency Virus (HIV) Patients admitted in a Tertiary Hospital in Sierra Leone	West African journal of medicine	g) No diagnoses
Costa et al.	Aplica√ß√£o dos escores MEWS (Modified Early Warning Score), MEDS (Mortality in Emergency Department Sepsis) e Sequential Organ Failure Assessment (SOFA) para classifica√ß√£o da gravidade dos pacientes internados em uma enfermaria de doen√ßas infecciosas	NA	c) Not all PLHIV
Costales et al.	Performance of Xpert Ultra nasopharyngeal swab for identification of tuberculosis deaths in northern Tanzania.	Clinical Microbiology and Infection	INCLUDED
Cox et al.	Needle autopsy to establish the cause of death in HIV-infected hospitalized adults in Uganda: a comparison to complete autopsy	Journal of acquired immune deficiency syndromes (1999)	i) Pre 2014
Cox et al.	Is Urinary Lipoarabinomannan the Result of Renal Tuberculosis? Assessment of the Renal Histology in an Autopsy Cohort of Ugandan HIV-Infected Adults	PloS one	i) Pre 2014
Craig et al.	Factors associated with admissions in HIV-1-infected individuals in the era of multiple HIV interventions	HIV Medicine	z) Duplicate paper in same study
Craik et al.	Challenges with targeted viral load testing for medical inpatients at Queen Elizabeth Central Hospital in Blantyre, Malawi	Malawi medical journal : the journal of Medical Association of Malawi	INCLUDED
Cranmer et al.	Brief Report: Performance of Tuberculosis Symptom Screening Among Hospitalized ART-Naive Children With HIV in Kenya	Journal of acquired immune deficiency syndromes (1999)	j) Other
Crowell et al.	Impact of hepatitis coinfection on hospitalization rates and causes in a multicenter cohort of persons living with HIV	Journal of acquired immune deficiency syndromes (1999)	i) Pre 2014
Cui et al.	Modelling clinical progression and health care utilization of HIV-positive patients in British Columbia prior to death	HIV medicine	i) Pre 2014
Cunha et al.	Mortality, survival and prognostic factors of people with AIDS in intensive care unit	Revista da Escola de Enfermagem da U S P	l) Cause of admission only
Cunneen et al.	A clinical review of HIV management and antiretroviral resistance in a rural south african hospital	Irish Journal of Medical Science	a) Irrelevant
da Silva et al.	Cryptococcosis in HIV-AIDS patients from Southern Brazil: Still a major problem	Journal de mycologie medicale	INCLUDED
Damasceno et al.	EVOLUCAO CLINICA DE PACIENTES HIV POSITIVOS EM UMA UNIDADE DE TERAPIA INTENSIVA, NO NORDESTE DO BRASIL	Brazilian Journal of Infectious Diseases	INCLUDED
Damasceno-Escoura et al.	Histoplasmosis in HIV-Infected Patients: Epidemiological, Clinical and Necropsy Data from a Brazilian Teaching Hospital	Mycopathologia	f) Everyone has same diagnosis
Dangor et al.	Temporal association in hospitalizations for tuberculosis, invasive pneumococcal disease and influenza virus illness in South African children	PloS one	f) Everyone has same diagnosis
Dart et al.	An evaluation of inpatient morbidity and critical care provision in Zambia	Anaesthesia	g) No diagnoses

Davies et al.	Provider-Initiated HIV Testing and Counselling for Children	PLoS Medicine	e) Not hospitalised
Davy-Mendez et al.	Hospitalization Rates and Outcomes Among Persons Living With Human Immunodeficiency Virus in the Southeastern United States, 1996-2016	Clinical infectious diseases : an official publication of the Infectious Diseases Society of America	i) Pre 2014
Davy-Mendez et al.	Racial, ethnic, and gender disparities in hospitalizations among persons with HIV in the United States and Canada, 2005-2015	AIDS (London, England)	i) Pre 2014
Davy-Mendez et al.	Hospitalization Rates and Causes Among Persons With HIV in the United States and Canada, 2005-2015	The Journal of infectious diseases	i) Pre 2014
Day et al.	Delirium in HIV infected patients admitted to acute medical wards post universal access to antiretrovirals in South Africa	South African Medical Journal	i) Pre 2014
Day et al.	Delirium in HIV-infected patients admitted to acute medical wards post universal access to antiretrovirals in South Africa	South African medical journal = Suid-Afrikaanse tydskrif vir geneeskunde	i) Pre 2014
de Campos et al.	The impact of highly active antiretroviral therapy on the burden of bacterial lower respiratory tract infections in children	South African medical journal = Suid-Afrikaanse tydskrif vir geneeskunde	e) Not hospitalised
de Lemos et al.	Mortality of children following a diagnosis of HIV infection in northeastern Brazil	Brazilian Journal of Infectious Diseases	e) Not hospitalised
de Leotoing et al.	Costs associated with hospitalization in HIV-positive patients in France	AIDS (London, England)	g) No diagnoses
De Matos et al.	Mortality predictive factors of people living with human immunodeficiency virus and bloodstream infection	International journal of infectious diseases : IJID : official publication of the International Society for Infectious Diseases	i) Pre 2014
de Oliveira et al.	Epidemiology of invasive fungal infections in patients with acquired immunodeficiency syndrome at a reference hospital for infectious diseases in Brazil	Mycopathologia	i) Pre 2014
Decano et al.	Adherence to antiretroviral therapy in hospitalized hiv-positive patients	Open Forum Infectious Diseases	g) No diagnoses
Deiss et al.	Prevalence, characteristics and outcomes of patients with Cryptococcal meningitis in Maputo, Mozambique	Journal of the International AIDS Society	INCLUDED
Deiss et al.	High burden of cryptococcal antigenemia and meningitis among patients presenting at an emergency department in Maputo, Mozambique	PloS one	l) Cause of admission only
Del Carmen et al.	Feasibility of implementing a geriatic consultation service for hospitalized older adults with HIV	Journal of the American Geriatrics Society	h) All have a syndrome
Devulapally et al.	A Clinical Study on Opportunistic Infections among HIV/AIDS Patients Admitted in the Department of General Medicine of a Tertiary Care Hospital	NA	INCLUDED
Di Bella et al.	Clostridium difficile infection among hospitalized HIV-infected individuals: epidemiology and risk factors: results from a case-control study (2002-2013)	BMC infectious diseases	f) Everyone has same diagnosis
Dicko et al.	Reasons for hospitalization in HIV-infected children in West Africa	Journal of the International AIDS Society	i) Pre 2014
Diez et al.	COVID-19 in hospitalized HIV-positive and HIV-negative patients: A matched study	HIV medicine	f) Everyone has same diagnosis
Dignani et al.	Epidemiology of invasive fungal diseases on the basis of autopsy reports	F1000Prime Reports	c) Not all PLHIV
Dillon et al.	Malignancies in HIV infection: A 5 year retrospective analysis	HIV Medicine	c) Not all PLHIV
Dissanayake et al.	Incidence and significance of an elevated red blood cell distribution width among hospitalised HIV-infected adult patients	International journal of STD & AIDS	f) Everyone has same diagnosis
Dlamini et al.	A prospective study of the demographics, management and outcome of patients with acute kidney injury in Cape Town, South Africa	PloS one	f) Everyone has same diagnosis

Dollo et al.	[Retrospective study of neuromeningeal cryptococcosis in patients infected with HIV in the infectious diseases unit of university hospital of Casablanca, Morocco]	Etude retrospective de la cryptococcose neuromeningee chez les patients infectes par le VIH dans le service des maladies infectieuses du CHU de Casablanca, Maroc.	i) Pre 2014
Dondo et al.	Pneumococcal Conjugate Vaccine Impact on Meningitis and Pneumonia Among Children Aged <5 Years-Zimbabwe, 2010-2016	Clinical infectious diseases : an official publication of the Infectious Diseases Society of America	c) Not all PLHIV
Dondo et al.	Pneumococcal Conjugate Vaccine Impact on Meningitis and Pneumonia Among Children Aged <5 Years,ÄîZimbabwe, 2010,Äì2016	Clinical Infectious Diseases	a) Irrelevant
dos Santos et al.	UMA DECADA DE MORTALIDADE HOSPITALAR DE INTERNACOES ASSOCIADAS AO HIV/AIDS SEGUNDO VULNERABILIDADE SOCIAL EM CAMPINAS: UM ESTUDO DE COORTE RETROSPECTIVO	Brazilian Journal of Infectious Diseases	z) Duplicate paper in same study
Dovonou et al.	Morbidity and mortality in the Internal Medicine Service of CHDB over a period of three years (2009-2011)	Morbidite et mortalite dans le service de medecine interne du Centre Hospitalier Departemental du Borgou sur une periode de trois ans (2009-2011).	c) Not all PLHIV
Dowsing et al.	HIV testing in general medical admissions - a missed -opportunity	Clinical medicine (London, England)	g) No diagnoses
Dreyer et al.	Evaluation of factors and patterns influencing the 30-day readmission rate at a tertiary-level hospital in a resource-constrained setting in Cape Town, South Africa	South African Medical Journal	c) Not all PLHIV
Du et al.	Mortality-related risks in treatment-naive hospitalized AIDS patients with opportunistic infections in Southwest China	Future Virology	l) Cause of admission only
du Plooy et al.	Profile of Young South African Children Hospitalized With HIV: Cause for Concern	The Pediatric infectious disease journal	j) Other
Duarte et al.	Burden of Tuberculosis Hospitalizations in Portugal From 2000 to 2015	Archivos de Bronconeumologia	f) Everyone has same diagnosis
DuffyCaitlyn and Kenga et al.	Multiple Concurrent Illnesses Associated with Anemia in HIV-Infected and HIV-Exposed Uninfected Children Aged 6,Äì59 Months, Hospitalized in Mozambique	American Journal of Tropical Medicine and Hygiene	h) All have a syndrome
Duke et al.	Large-scale data reporting of paediatric morbidity and mortality in developing countries: It can be done	Archives of Disease in Childhood	c) Not all PLHIV
Dutertre et al.	Initiation of Antiretroviral Therapy Containing Integrase Inhibitors Increases the Risk of IRIS Requiring Hospitalization	Journal of acquired immune deficiency syndromes (1999)	f) Everyone has same diagnosis
Edmiston et al.	Multimorbidity, not human immunodeficiency virus (HIV) markers predicts unplanned admission among people with HIV in regional New South Wales	Internal medicine journal	g) No diagnoses
Elkhatiali et al.	An evaluation of challenges with the South African PMTCT HIV programme seen from the perspective of HIV-positive children admitted to the PICU	SAJCH South African Journal of Child Health	z) Duplicate paper in same study
Enane et al.	"We did not know what was wrong"-Barriers along the care cascade among hospitalized adolescents with HIV in Gaborone, Botswana	PloS one	l) Cause of admission only
Erscoiu et al.	Impact of HIV-1 infection on mortality among new diagnosed cases in a hospital in Bucharest	BMC Infectious Diseases	i) Pre 2014
Etyang et al.	Burden of disease in adults admitted to hospital in a rural region of coastal Kenya: an analysis of data from linked clinical and demographic surveillance systems	The Lancet. Global health	g) No diagnoses
Evans et al.	Incidence, aetiology and outcome of community-acquired acute kidney injury in medical admissions in Malawi	BMC nephrology	k) Duplicate record

Falci et al.	Histoplasmosis, An Underdiagnosed Disease Affecting People Living With HIV/AIDS in Brazil: Results of a Multicenter Prospective Cohort Study Using Both Classical Mycology Tests and Histoplasma Urine Antigen Detection	Open forum infectious diseases	h) All have a syndrome
Farhan et al.	In-Patient Mortality among PLHIV: A 7-Year Hospital-Based Retrospective Study in Coastal South India	Journal of the International Association of Providers of AIDS Care	i) Pre 2014
Farooq et al.	A seven-month prospective review of HIV admissions to a regional infectious disease unit in Manchester, UK	HIV Medicine	INCLUDED
Feder et al.	Hiv Infection And Comorbidity As Predictors Of Palliative Care Consultation In Adults Hospitalized With Heart Failure	American Journal of Respiratory and Critical Care Medicine	g) No diagnoses
Felsen et al.	An expanded human immunodeficiency virus (HIV) testing strategy leveraging the electronic medical record uncovers undiagnosed infection among hospitalized patients	Open Forum Infectious Diseases	g) No diagnoses
Ferraz et al.	Clinical characteristics of newly diagnosed HIV-infected patients and risk factors for late presentation: A Portuguese cohort	Journal of the International AIDS Society	i) Pre 2014
Figueiredo-Mello et al.	Prospective etiological investigation of community-acquired pulmonary infections in hospitalized people living with HIV	Medicine	f) Everyone has same diagnosis
Figueroa-Agudelo et al.	Sociodemographic and clinical characteristics of patients with new HIV diagnosis	Infectio	i) Pre 2014
Fiseha et al.	Prevalence and associated factors of impaired renal function and albuminuria among adult patients admitted to a hospital in Northeast Ethiopia	PloS one	g) No diagnoses
Fleming et al.	Hospitalization rates and diagnoses vary by age group among persons with HIV (PWH) in 2014-2015	Open Forum Infectious Diseases	g) No diagnoses
Fleming et al.	Risk factors for increased hospital length of stay among PWH, 2014-2015	Topics in Antiviral Medicine	INCLUDED
Fleming et al.	U.S. Hospitalization rates and reasons stratified by age among persons with HIV 2014-15	AIDS care	INCLUDED
Florescu et al.	Mortality causes in infectious diseases	Romanian Journal of Legal Medicine	g) No diagnoses
Focaccia et al.	Clinical and epidemiology evaluation of Aids-infected patients hospitalized between 2011 and 2016 in the Santos region of Brazil	Revista da Sociedade Brasileira de Medicina Tropical	INCLUDED
Freercks et al.	Scope and mortality of adult medical ICU patients in an Eastern Cape tertiary hospital	The Southern African journal of critical care : the official journal of the Critical Care Society	INCLUDED
Frigati et al.	Hospitalization in South African Adolescents With Perinatally Acquired HIV on Antiretroviral Therapy	The Pediatric infectious disease journal	h) All have a syndrome
Gaillet et al.	Outcomes in critically Ill HIV-infected patients between 1997 and 2020: analysis of the OUTCOMEREA multicenter cohort	Critical care (London, England)	e) Not hospitalised
Gama et al.	Immunologic biomarkers, morbidity and mortality among HIV patients hospitalised in a Tertiary Care Hospital in the Brazilian Amazon	BMC infectious diseases	i) Pre 2014
Gao et al.	Performance of Xpert MTB/RIF for Diagnosis of Tuberculosis in HIV-Infected People in China: A Retrospective, Single-Center Study	Medical science monitor : international medical journal of experimental and clinical research	a) Irrelevant
Garcia et al.	Severe bacterial non-AIDS infections in HIV women	Revista Espanola de Quimioterapia	a) Irrelevant
Garcia-Basteiro et al.	Determining the role of Xpert MTB/RIF in diagnosing tuberculosis in post-mortem tissues	European Respiratory Journal	INCLUDED
Garcia-Basteiro et al.	Unmasking the hidden tuberculosis mortality burden in a large postmortem study in Mozambique	European Respiratory Journal	k) Duplicate record

Garcia-Basteiro et al.	Unmasking the hidden tuberculosis mortality burden in a large post mortem study in Maputo Central Hospital, Mozambique	The European respiratory journal	l) Cause of admission only
Garcia-Basteiro et al.	Performance of the Xpert MTB/RIF ultra assay for determining cause of death by TB in tissue samples obtained by minimally invasive autopsies	Chest	l) Cause of admission only
Garcia-BasteiroAlberto et al.	Unmasking the hidden tuberculosis mortality burden in a large <i>post mortem study in Maputo Central Hospital, Mozambique	The European Respiratory Journal	e) Not hospitalised
Gbeasor-Komlanvi et al.	Predictors of three-month mortality among hospitalized older adults in Togo	BMC Geriatrics	c) Not all PLHIV
Gbeasor-Komlanvi et al.	HIV testing uptake and prevalence among hospitalized older adults in Togo: A cross-sectional study	PloS one	g) No diagnoses
Gel et al.	Prevalence, clinical pattern and immediate outcomes of HIV-infected children admitted to Al Sabah Children's Hospital, South Sudan	South Sudan Medical Journal	g) No diagnoses
Gemaque et al.	Prevalence of oral lesions in hospitalized patients with infectious diseases in northern Brazil	TheScientificWorldJournal	c) Not all PLHIV
Georges et al.	Causes and outcome of acute kidney injury amongst adults patients in two hospitals of different category in Cameroon; a 5 year retrospective comparative study	BMC Nephrology	h) All have a syndrome
Ghahramani et al.	Predictive factors for positive HIV test results in a hospital setting	Archives of Clinical Infectious Diseases	g) No diagnoses
Ghasemzadeh et al.	Epidemiologic evaluation of HIV/AIDS patients admitted in Southern Of Iran in 12 years	Acta Medica Mediterranea	i) Pre 2014
Gibson et al.	Thirty-day readmissions among HIV-infected individuals at a safety-net hospital: Predictors and preventability	Open Forum Infectious Diseases	INCLUDED
Gilbert et al.	Risk factors for development of acute kidney injury in hospitalised adults in Zimbabwe	PloS one	g) No diagnoses
Gilliams et al.	DESCRIPTION OF PATIENTS WITH HIV/AIDS ATTENDING A SUPPLEMENTAL PALLIATIVE CARE CLINIC AND IMPACT ON VIRAL LOAD SUPPRESSION, HOSPITALIZATION, AND RETENTION IN CARE	Journal of Investigative Medicine	g) No diagnoses
Gilliams et al.	Increased Retention in Care After a Palliative Care Referral Among People Living With HIV	Journal of acquired immune deficiency syndromes (1999)	g) No diagnoses
Giordano et al.	A Randomized Trial to Test a Peer Mentor Intervention to Improve Outcomes in Persons Hospitalized With HIV Infection	Clinical infectious diseases : an official publication of the Infectious Diseases Society of America	g) No diagnoses
Girma et al.	Time to occurrence, predictors, and patterns of opportunistic infections incidence among HIV-positive patients attending Antiretroviral Therapy Clinic of Salale University Comprehensive Specialized Hospital: A retrospective cohort study	Medicine	e) Not hospitalised
Goetghebuer et al.	Initiation of Antiretroviral Therapy Before Pregnancy Reduces the Risk of Infection-related Hospitalization in Human Immunodeficiency Virus-exposed Uninfected Infants Born in a High-income Country	Clinical infectious diseases : an official publication of the Infectious Diseases Society of America	c) Not all PLHIV
Gonzaga Ferreira et al.	Predictors of mobility impairment after hospitalization in a reference hospital among individuals with infectious diseases	Fisioterapia e Pesquisa	a) Irrelevant
Gonzalez-Fernandez et al.	Advanced presentation among HIV/AIDS patients despite universal access to antiretroviral therapy in northern Mexico	Journal of the International AIDS Society	l) Cause of admission only
Gopalan et al.	Factors influencing decisions to admit or refuse patients entry to a South African tertiary intensive care unit	South African medical journal = Suid-Afrikaanse tydskrif vir geneeskunde	g) No diagnoses

Gordon et al.	Implementation of an in-patient pediatric mortality reduction intervention, Gondar University Hospital, Ethiopia	Public health action	c) Not all PLHIV
Goswami et al.	A Minority of Patients Newly Diagnosed with AIDS Are Started on Antiretroviral Therapy at the Time of Diagnosis in a Large Public Hospital in the Southeastern United States	Journal of the International Association of Providers of AIDS Care	i) Pre 2014
Govender et al.	The characteristics of HIV/AIDS patients with deep vein thrombosis at Dr George Mukhari Academic Hospital	International Journal of Infectious Diseases	g) No diagnoses
Govender et al.	Clinical risk factors for in-hospital mortality in older adults with HIV infection: findings from a South African hospital administrative dataset	The Pan African medical journal	i) Pre 2014
Grant et al.	Clinical and immunologic predictors of death after an acute opportunistic infection: results from ACTG A5164	HIV clinical trials	f) Everyone has same diagnosis
Grant et al.	High-Cost, High-Need Users of Acute Unscheduled HIV Care: A Cross-Sectional Study	Open forum infectious diseases	INCLUDED
Greene et al.	Geriatric conditions in older HIV-positive adults and association with healthcare utilization	Journal of the American Geriatrics Society	k) Duplicate record
Guedes et al.	Visceral Leishmaniasis in Hospitalized HIV-Infected Patients in Pernambuco, Brazil	The American journal of tropical medicine and hygiene	INCLUDED
GuillV©n et al.	Causas de muerte en pacientes infectados con VIH en el aV±o 2017. Hospital Vargas de Caracas	Bol. venez. infectol	i) Pre 2014
Guinhouya et al.	Le VIH chez le malade neurologique aux CHU de Lome	Journal de la Recherche Scientifique de l'Universite de Lome	h) All have a syndrome
Guo et al.	Customized order-entry sets can prevent antiretroviral prescribing errors: a novel opportunity for antimicrobial stewardship	P & T : a peer-reviewed journal for formulary management	g) No diagnoses
Guo et al.	[Research on the causes of death associated with combined effects of HBV and HCV infection in patients with acquired immunodeficiency syndrome]	Zhonghua gan zang bing za zhi = Zhonghua ganzangbing zazhi = Chinese journal of hepatology	a) Irrelevant
Gupta-Wright et al.	Rapid urine-based screening for tuberculosis in HIV-positive patients admitted to hospital in Africa (STAMP): a pragmatic, multicentre, parallel-group, double-blind, randomised controlled trial	Lancet (London, England)	g) No diagnoses
Gupta-Wright et al.	Urine-based screening for tuberculosis: A randomized trial in HIV-positive inpatients	Topics in Antiviral Medicine	INCLUDED
Gupta-Wright et al.	Tuberculosis in hospitalised patients with HIV: clinical characteristics, mortality, and implications from the STAMP trial	Clinical infectious diseases : an official publication of the Infectious Diseases Society of America	f) Everyone has same diagnosis
Gutierrez-Velilla et al.	Identifying risk factors for HIV-positive test results in walk-in and hospitalized patients in a Mexico City HIV clinic: a descriptive study	International journal of STD & AIDS	g) No diagnoses
Haachambwa et al.	Care Continuum and Postdischarge Outcomes Among HIV-Infected Adults Admitted to the Hospital in Zambia	Open forum infectious diseases	h) All have a syndrome
Habib et al.	Comparison of home and hospital deaths among patients on anti-retroviral therapy (ART): A clinical and verbal autopsy study	HIV and AIDS Review	i) Pre 2014
Hadlock et al.	Risk factors for potentially preventable hospital readmissions among persons living with human immunodeficiency virus infection	AIDS care	g) No diagnoses
Hahn et al.	Impact of endemic HIV on emergency care service delivery in South Africa	South African medical journal = Suid-Afrikaanse tydskrif vir geneeskunde	INCLUDED
Haidari et al.	Neurological admissions in people living with HIV: A review of inpatient admissions to an inner-city teaching hospital	HIV Medicine	i) Pre 2014

Haile Hantalo et al.	Isolation and Antibiotic Susceptibility Pattern of Bacterial Uropathogens and Associated Factors Among Adult People Living with HIV/AIDS Attending the HIV Center at Wolaita Sodo University Teaching Referral Hospital, South Ethiopia	HIV/AIDS (Auckland, N.Z.)	l) Cause of admission only
Halman et al.	Complex care needs of patients with late-stage HIV disease: a retrospective study	AIDS care	h) All have a syndrome
Harris et al.	Paediatric deaths in a tertiary government hospital setting, Malawi	Paediatrics and international child health	c) Not all PLHIV
Hart et al.	How advanced is the epidemiological transition in Papua New Guinea? New evidence from verbal autopsy	International Journal of Epidemiology	e) Not hospitalised
Hashmi et al.	Intensive care admissions of HIV infected patients: The effect of HAART on outcomes in an inner-city hospital	Chest	g) No diagnoses
Havugimana et al.	Prevalence of anemia, associated risk factors and outcome in CHUK, Rwanda: a prospective observational study	medRxiv	g) No diagnoses
Heinz et al.	HOSPITALIZATION RATES AMONG EMERGENCY ROOM-DIAGNOSED HIV+ PATIENTS IN THE US AND EU5	Value in Health	g) No diagnoses
Heller et al.	Implementing Advanced HIV Disease Care for Inpatients in a Referral Hospital in Malawi - Demand, Results and Cost Implications	Annals of global health	l) Cause of admission only
Hellinger et al.	Hospital Use by Persons With HIV in the 21st Century: A 5-State Study	Medical care	i) Pre 2014
Hill-Tout et al.	Routine HIV testing in acute medical admissions in a high prevalence area reduces morbidity and mortality of HIV: a full cycle audit	International journal of STD & AIDS	g) No diagnoses
Himwaze et al.	Prevalence of Human Immunodificency Virus, Hepatitis B, and Hepatitis C viral infections among forensic autopsy cases at the University Teaching Hospital in Lusaka, Zambia	Forensic Science International: Reports	e) Not hospitalised
Ho et al.	Impact of HIV on the burden and severity of influenza illness in adults in Malawi: A cohort and case-control study	The Lancet	h) All have a syndrome
Ho et al.	Impact of Human Immunodeficiency Virus on the Burden and Severity of Influenza Illness in Malawian Adults: A Prospective Cohort and Parallel Case-Control Study	Clinical infectious diseases : an official publication of the Infectious Diseases Society of America	h) All have a syndrome
Hoffmann et al.	Post-hospital mortality and readmission among HIV-infected adults in South Africa	Topics in Antiviral Medicine	z) Duplicate paper in same study
Hoffmann et al.	Readmission and death following hospitalization among people with HIV in South Africa	PloS one	INCLUDED
Holland et al.	Why are patients living with HIV still admitted to hospital?	HIV Medicine	z) Duplicate paper in same study
Howlett et al.	Hiv-related acute respiratory admissions-good outcomes and an opportunity for testing	Thorax	h) All have a syndrome
Hoyos Pulgarin et al.	Closing gaps in histoplasmosis: clinical characteristics and factors associated with probable/histoplasmosis in HIV/AIDS hospitalized patients, a retrospective cross-sectional study in two tertiary centers in Pereira, Colombia	AIDS research and therapy	l) Cause of admission only
Huerga et al.	Urine Lipoarabinomannan Testing for All HIV Patients Hospitalized in Medical Wards Identifies a Large Proportion of Patients With Tuberculosis at Risk of Death	Open forum infectious diseases	INCLUDED
Hunter et al.	Case mix of patients managed in the resuscitation area of a district-level public hospital in Cape Town	African journal of emergency medicine : Revue africaine de la medecine d'urgence	g) No diagnoses
Hurst et al.	Trends in Diagnoses Among Hospitalizations of HIV-infected Children and Adolescents in the United States: 2003-2012	The Pediatric infectious disease journal	i) Pre 2014
Hurtado et al.	Mortality due to Cryptococcus neoformans and Cryptococcus gattii in low-income settings: an autopsy study	Scientific reports	k) Duplicate record
Jacobs et al.	Implementing opt-out HIV testing for adolescents-will they just say "NO"? UCSF benioff children's hospital Oakland HIV focus	Journal of Adolescent Health	g) No diagnoses

Jacobs et al.	Initiation of Antiretroviral Therapy in the Hospital Is Associated with Linkage to Human Immunodeficiency Virus (HIV) Care for Persons Living with HIV and Substance Use Disorder	Clinical Infectious Diseases	g) No diagnoses
Jain et al.	Surveillance of tuberculosis co-infection among HIV infected patients and their CD4+ cell count profile	Asian Pacific Journal of Tropical Disease	i) Pre 2014
Janocha-Litwin et al.	Neurological Disorders of Patients Living with HIV Hospitalized in Infectious Departments of the Specialist Hospital in Lower Silesia in Poland	Healthcare (Basel, Switzerland)	h) All have a syndrome
Jardim et al.	Thirty-day readmission rates in a cohort of people living with HIV in southern Brazil, 2015 to 2017	International journal of STD & AIDS	k) Duplicate record
Jefferys et al.	Hospitalisation among elite controllers	HIV Treatment Bulletin	d) Not primary research
Jemal et al.	Prevalence of Cryptococcal Antigenemia and Associated Factors among HIV/AIDS Patients at Felege-Hiwot Referral Hospital, Bahir Dar, Northwest Ethiopia	International journal of microbiology	INCLUDED
Jensen et al.	Patterns of disease on admission to children's wards and changes during a COVID-19 outbreak in KwaZulu-Natal Province, South Africa	South African Medical Journal	c) Not all PLHIV
Jereen et al.	Prevalence of HIV associated non-AIDS conditions and associated risk factors among hospitalized HIV-infected Patients in India	Open Forum Infectious Diseases	INCLUDED
Jiang et al.	Effects of Talaromyces marneffei infection on mortality of HIV/AIDS patients in southern China: a retrospective cohort study	Clinical microbiology and infection : the official publication of the European Society of Clinical Microbiology and Infectious Diseases	z) Duplicate paper in same study
Jipa et al.	Tuberculosis in people living with human immunodeficiency virus might be overlooked	Romanian Journal of Legal Medicine	f) Everyone has same diagnosis
Johnson et al.	Hospitalisation across the ages: Transitioning young people with perinatally acquired HIV (PaHIV)	HIV Medicine	i) Pre 2014
Johnston et al.	The Prevalence of Drug-Drug Interactions with Antiretroviral Therapy in Human Immunodeficiency Virus-Infected Patients in the Intensive Care Unit	Journal of pharmacy practice	g) No diagnoses
Johnstone et al.	Epidemiology of invasive bacterial infections in pneumococcal conjugate vaccine-vaccinated and -unvaccinated children under 5 years of age in Soweto, South Africa: a cohort study from a high-HIV burden setting	Paediatrics and international child health	i) Pre 2014
Joseph Mollel et al.	Causes of death among a cohort of HIV-infected adults in rural Tanzania	HIV Medicine	e) Not hospitalised
Juniper et al.	The majority of HIV inpatient admissions in south London occur in previously diagnosed patients: Is poor engagement in care the final hurdle?	HIV Medicine	INCLUDED
Kamis et al.	Risk Factors for Hospitalization in People With HIV and COVID-19	Journal of acquired immune deficiency syndromes (1999)	f) Everyone has same diagnosis
Kanyama et al.	Implementation of tuberculosis and cryptococcal meningitis rapid diagnostic tests amongst patients with advanced HIV at Kamuzu Central Hospital, Malawi, 2016-2017	BMC infectious diseases	INCLUDED
Karat et al.	Autopsy Prevalence of Tuberculosis and Other Potentially Treatable Infections among Adults with Advanced HIV Enrolled in Out-Patient Care in South Africa	PloS one	INCLUDED
Karat et al.	Measuring mortality due to HIV-associated tuberculosis among adults in South Africa: Comparing verbal autopsy, minimally-invasive autopsy, and research data	PloS one	i) Pre 2014
Karat et al.	Performance of verbal autopsy methods in estimating HIV-associated mortality among adults in South Africa	BMJ global health	e) Not hospitalised
Karat et. al.	Autopsy Prevalence of Tuberculosis and Other Potentially Treatable Infections among Adults with Advanced HIV Enrolled in Out-Patient Care in South Africa.	PloS one	INCLUDED
Kavuma Mwanje et al.	Association between CD4 T cell counts and the immune status among adult critically ill HIV-negative patients in intensive care units in Uganda	AAS open research	c) Not all PLHIV

Kayambankadzanja et al.	The Prevalence and Outcomes of Sepsis in Adult Patients in Two Hospitals in Malawi	The American journal of tropical medicine and hygiene	g) No diagnoses
Kazibwe et al.	HIV, tuberculosis, diabetes mellitus and hypertension admissions and premature mortality among adults in Uganda from 2011 to 2019: is the tide turning?	Tropical medicine and health	INCLUDED
Kebede et al.	The role of chest radiography in the diagnosis of bacteriologically confirmed pulmonary tuberculosis in hospitalised Xpert MTB/RIF-negative patients	ERJ open research	h) All have a syndrome
Kelly et al.	The impact of recreational drug use on inpatient admissions	HIV Medicine	INCLUDED
Kerkhoff et al.	Diagnostic sensitivity of SILVAMP TB-LAM (FujiLAM) point-of-care urine assay for extra-pulmonary tuberculosis in people living with HIV	European Respiratory Journal	i) Pre 2014
Kershaw et al.	Audit of Early Mortality among Patients Admitted to the General Medical Ward at a District Hospital in Botswana	Annals of global health	c) Not all PLHIV
Khan et al.	Neurological manifestations of HIV/AIDS to tertiary care hospital in KP, Peshawar, Pakistan	International Journal of Infectious Diseases	h) All have a syndrome
Khawcharoenporn et al.	Enhanced inpatient rounds, appointment reminders, and patient education improved HIV care engagement following hospital discharge	International journal of STD & AIDS	g) No diagnoses
Kimmel et al.	Characteristics associated with motivation to stop substance use and improve skin and needle hygiene among hospitalized patients who inject drugs	Substance abuse	c) Not all PLHIV
Kirby et al.	HOSPITALISATION IN HIV PATIENTS: ARE THE CAUSES OF ADMISSION CHANGING?	Sexually Transmitted Infections	l) Cause of admission only
Kitila et al.	Under-five mortality and associated factors in southeastern Ethiopia	PloS one	c) Not all PLHIV
Klinger et al.	Mortality Among Inpatients After the Initiation of 'Treat All' With Dolutegravir in Botswana	Open Forum Infectious Diseases	INCLUDED
Kozhevnikova et al.	Opportunistic diseases in patients with HIV infection in the intensive care unit	Terapevticheskii arkhiv	i) Pre 2014
Krishnam et al.	Utility of routine non-gated CT chest in detection of subclinical atherosclerotic calcifications of coronary arteries in hospitalised HIV patients	The British journal of radiology	h) All have a syndrome
Kruger et al.	Outcome of children admitted to a general highcare unit in a regional hospital in the Western Cape, South Africa	South African Journal of Child Health	c) Not all PLHIV
Kruisselbrink et al.	Monitoring of medical and surgical patients in Mulago hospital, Uganda: A pilot observational study using the modified early warning score	American Journal of Respiratory and Critical Care Medicine	c) Not all PLHIV
Kruisselbrink et al.	Modified Early Warning Score (MEWS) Identifies Critical Illness among Ward Patients in a Resource Restricted Setting in Kampala, Uganda: A Prospective Observational Study	PloS one	c) Not all PLHIV
Krutikov et al.	HIV-related medical admissions to an HIV specialist inpatient unit: Quality standards and outcomes	HIV Medicine	l) Cause of admission only
Kukoyi et al.	Viral load monitoring and antiretroviral treatment outcomes in a pediatric HIV cohort in Ghana	BMC infectious diseases	i) Pre 2014
Kwizera et al.	Clinical characteristics and short-term outcomes of HIV patients admitted to an African intensive care unit	Intensive Care Medicine Experimental	g) No diagnoses
Kwizera et al.	Clinical Characteristics and Short-Term Outcomes of HIV Patients Admitted to an African Intensive Care Unit	Critical care research and practice	i) Pre 2014
LaCourse et al.	Implementation and Operational Research: Implementation of Routine Counselor-Initiated Opt-Out HIV Testing on the Adult Medical Ward at Kamuzu Central Hospital, Lilongwe, Malawi	Journal of acquired immune deficiency syndromes (1999)	g) No diagnoses
LaCourse et al.	Urine Tuberculosis Lipoarabinomannan Predicts Mortality in Hospitalized Human Immunodeficiency Virus-Infected Children	Clinical infectious diseases : an official publication of the Infectious Diseases Society of America	e) Not hospitalised

LaCourse et al.	Stool Xpert MTB/RIF and urine lipoarabinomannan for the diagnosis of tuberculosis in hospitalized HIV-infected children	AIDS (London, England)	l) Cause of admission only
Lagrutta et al.	La Unidad Febril de Urgencias del Hospital Mu√±iz frente a COVID-19, HIV y tuberculosis	Medicina (Buenos Aires)	c) Not all PLHIV
Laher et al.	Patterns of presentation and predictors of ICU mortality among HIV infected patients	Critical Care	INCLUDED
Laher et al.	Predictors of prolonged hospital stay in HIV-positive patients presenting to the emergency department	PloS one	g) No diagnoses
Laher et al.	Antiretroviral therapy non-adherence among HIV-positive patients presenting to an emergency department in Johannesburg, South Africa: Associations and reasons	South African Medical Journal	g) No diagnoses
Lai et al.	Prevalence and risk factors of anaemia in hospitalised HIV-infected patients in southeast China: a retrospective study	Epidemiology and infection	j) Other
Lakoh et al.	Causes of hospitalization and predictors of HIV-associated mortality at the main referral hospital in Sierra Leone: a prospective study	BMC public health	INCLUDED
Lang et al.	Reduction of paediatric hospital mortality in a Malawian referral hospital	Pediatric Critical Care Medicine	c) Not all PLHIV
Lanjewar et al.	Pathologic lesions in children with acquired immunodeficiency syndrome an autopsy study of 11 cases from Mumbai, India	Indian journal of pathology & microbiology	i) Pre 2014
Lara-Medrano et al.	Prolonged hospital stays and associated factors in patients receiving care in a HIV/AIDS clinic in Mexico City	Journal of the International AIDS Society	INCLUDED
Laso et al.	Analysis of HIV patients hospitalization, clinical situation and related factors	International Journal of Clinical Pharmacy	c) Not all PLHIV
Laundy et al.	A service review of patients living with HIV admitted to an infectious diseases department...21st National HIV Nurses Association Annual Conference, 27-28 June 2019, Manchester Conference Centre, Manchester, UK	HIV Nursing	INCLUDED
Laurence et al.	Depression and the Likelihood of Hospital Admission from the Emergency Department among Older Patients with HIV	Journal of health care for the poor and underserved	g) No diagnoses
Lawson-Ananissoh et al.	Les facteurs associes au deces des patients infectes par le virus de l'immunodeficience humaine hospitalises dans le service d'hepato-gastroenterologie du CHU Campus de Lome, Togo, Factors associated with the death of patients infected with the human immunodeficiency virus hospitalized in the gastroenterology department of the Campus Teaching Hospital of Lome, Togo	Medecine et sante tropicales	h) All have a syndrome
Leon Alonso et al.	Morbidity in patients infected with HIV/AIDS in an Intensive Care Unit	Morbilidad en pacientes infectados por VIH/SIDA en una Unidad de Cuidados Intensivos.	l) Cause of admission only
Leonard et al.	Late presentation among patients diagnosed with HIV in an inpatient setting	Open Forum Infectious Diseases	l) Cause of admission only
Letang et al.	Minimally Invasive Tissue Sampling: A Tool to Guide Efforts to Reduce AIDS-Related Mortality in Resource-Limited Settings	Clinical infectious diseases : an official publication of the Infectious Diseases Society of America	INCLUDED
Li et al.	In-hospital Mortality and Causes of Death in People Diagnosed With HIV in a General Hospital in Shenyang, China: A Cross-Sectional Study	Frontiers in public health	INCLUDED
Liang et al.	Clinical characteristics and pathogens of 143 cases of AIDS-associated bloodstream infection	Chinese Journal of Infection and Chemotherapy	h) All have a syndrome
Lima et al.	Mortalidade, sobrevida e fatores progn√≥sticos de pessoas com AIDAS em Unidade de Terapia Intensiva	NA	l) Cause of admission only
Liping et al.	Epidemiological characteristics and clinical analysis of 97 AIDS patients	European Journal of Inflammation	i) Pre 2014

Liu et al.	Analysis of clinical features of 2 992 inpatients with HIV/AIDS in Yunnan Province	Chinese Journal of Dermatovenereology	INCLUDED
Liu et al.	Trends in rates and causes of hospitalization among people living with HIV in the antiretroviral therapy era: A retrospective cohort study in China, 2008-2020	Frontiers in public health	h) All have a syndrome
Liusha et al.	A comparative study of Mycobacterium Tuberculosis in hospitalised adult HIV infected patients with normal and abnormal renal function at the University Teaching Hospital, Lusaka, Zambia	Medical Journal of Zambia	i) Pre 2014
Long et al.	The High Cost of HIV-Positive Inpatient Care at an Urban Hospital in Johannesburg, South Africa	PloS one	i) Pre 2014
Lopes et al.	Vulnerability factors associated with HIV/AIDS hospitalizations: a case-control study	Revista brasileira de enfermagem	g) No diagnoses
Luggya et al.	Trauma unit management and outcomes at an urban tertiary hospital in sub-Saharan Africa: a descriptive study	African health sciences	c) Not all PLHIV
Lukoko et al.	Investigating SOFA, delta-SOFA and MPM-III for mortality prediction among critically ill patients at a private tertiary hospital ICU in Kenya: A retrospective cohort study	PloS one	c) Not all PLHIV
Macken et al.	Bedside paediatric HIV testing in Malawi: Impact on testing rates	Malawi medical journal : the journal of Medical Association of Malawi	g) No diagnoses
Madaline et al.	A clinical snapshot of hospitalized, newly diagnosed, HIV-positive Malawian children reveals opportunities for improved HIV healthcare delivery	American Journal of Tropical Medicine and Hygiene	i) Pre 2014
Madaline et al.	Rapid Diagnostic Testing of Hospitalized Malawian Children Reveals Opportunities for Improved HIV Diagnosis and Treatment	The American journal of tropical medicine and hygiene	l) Cause of admission only
Maheswaran et al.	Economic costs and health-related quality of life outcomes of hospitalised patients with high HIV prevalence: A prospective hospital cohort study in Malawi	PloS one	INCLUDED
Mahmoudi et al.	Epidemiologic and clinical findings of children with acquired immunodeficiency syndrome in four provinces of Iran	Wiener medizinische Wochenschrift (1946)	i) Pre 2014
Maia et al.	Fatores associados a √≥bitos de adultos hospitalizados vivendo com AIDS	Rev. baiana sa√ʃde p√ʃblica	g) No diagnoses
Mamuye et al.	Point-of-Care Testing for Cryptococcal Disease Among Hospitalized Human Immunodeficiency Virus-Infected Adults in Ethiopia	The American journal of tropical medicine and hygiene	INCLUDED
Mamuye et al.	Point-of-Care Testing for Cryptococcal Disease Among Hospitalized Human Immunodeficiency Virus,ÄìInfected Adults in Ethiopia	American Journal of Tropical Medicine and Hygiene	i) Pre 2014
Manciuc et al.	Clinico-epidemiological aspects and psychosocial HIV infection of a patient in the period of fertila age-HIV-AIDS experience, Regional Centre, Iasi	Clujul Medical	h) All have a syndrome
Mandla et al.	Prevalence of severe acute malnutrition and its effect on under-five mortality at a regional hospital in South Africa	South African Journal of Clinical Nutrition	c) Not all PLHIV
Mangurian et al.	Underdetection of pre-existing HIV/AIDS during psychiatric hospitalizations	AIDS (London, England)	h) All have a syndrome
Mapera et al.	Clinico-epidemiological profile of children living with HIV/AIDS managed at Heal Africa Hospital, Goma, Democratic Republic of the Congo	African health sciences	e) Not hospitalised
Maphula et al.	Patterns of presentation and survival of HIV-infected patients admitted to a tertiary-level intensive care unit	HIV medicine	INCLUDED
Martin-Onraet et al.	In-hospital mortality in HIV-infected patients: 10 years after the implementation of universal access to HAART in Mexico	Salud publica de Mexico	i) Pre 2014
Martinez et al.	Infectious cause of death determination using minimally invasive autopsies in developing countries	Diagnostic microbiology and infectious disease	k) Duplicate record
Marwaha et al.	The transfer times and outcomes of a tertiary in-patient HIV ward	HIV Medicine	INCLUDED

Masoza et al.	Prevalence and outcome of HIV infected children admitted in a tertiary hospital in Northern Tanzania	BMC pediatrics	INCLUDED
Massah et al.	Hospitalizations & mortality differ by gender among long-term art patients in Uganda	Topics in Antiviral Medicine	g) No diagnoses
Maxwell et al.	A changing pattern of HIV inpatient admissions and complexity: from late diagnoses to defaulters	HIV Medicine	l) Cause of admission only
Mbewe et al.	Advanced HIV disease management practices within inpatient medicine units at a referral hospital in Zambia: a retrospective chart review	AIDS research and therapy	g) No diagnoses
McMorrow et al.	The Impact of Human Immunodeficiency Virus Exposure on Respiratory Syncytial Virus-associated Severe Respiratory Illness in South African Infants, 2011-2016	Clinical infectious diseases : an official publication of the Infectious Diseases Society of America	h) All have a syndrome
Mdala et al.	Causes of mortality and associated modifiable health care factors for children (< 5-years) admitted at Onandjokwe Hospital, Namibia	African journal of primary health care & family medicine	c) Not all PLHIV
Meng et al.	Spectrum and mortality of opportunistic infections among HIV/AIDS patients in southwestern China	European journal of clinical microbiology & infectious diseases : official publication of the European Society of Clinical Microbiology	INCLUDED
Mgamb et al.	Assessing the Leading Causes of Mortality in less than Fiveyear old Children in Migori District, Kenya, 2011	Pan African Medical Journal	g) No diagnoses
Mgori et al.	HIV and/or AIDS-related deaths and modifiable risk factors: A descriptive study of medical admissions at Oshakati Intermediate Hospital in Northern Namibia	African journal of primary health care & family medicine	i) Pre 2014
Mignano et al.	Results and Implications of Routine HIV Testing in the Inpatient Setting: A Descriptive Analysis	Population health management	g) No diagnoses
Milan et al.	Results from the first audit of an intensive care unit in Botswana	Southern African Journal of Critical Care	g) No diagnoses
Mishore et al.	Hospitalization and Predictors of Inpatient Mortality among HIV-Infected Patients in Jimma University Specialized Hospital, Jimma, Ethiopia: Prospective Observational Study	AIDS research and treatment	l) Cause of admission only
Mkoko et al.	HIV-positive patients in the intensive care unit: A retrospective audit	South African medical journal = Suid-Afrikaanse tydskrif vir geneeskunde	i) Pre 2014
Mody et al.	Audit of HIV testing in a multispecialty acute admissions unit in a London general hospital	Sexually transmitted infections	g) No diagnoses
Moein et al.	Evaluation of healthcare usage rate in HIV/AIDS patients in Isfahan, Iran in 2018	HIV and AIDS Review	g) No diagnoses
Montgomery et al.	HIV screening of admissions to infectious disease unit	Scottish Medical Journal	i) Pre 2014
Montufar Andrade et al.	Epidemiology of human immunodeficiency virus infection in inpatients in a teaching hospital of high complexity in Medellin, Colombia	Infectio	i) Pre 2014
Moodley et al.	The impact of an unknown HIV serostatus on inpatient mortality	The Pan African medical journal	g) No diagnoses
Mopeli et al.	An audit of primary medical conditions in children admitted to the paediatric intensive care unit of charlotte maxeke johannesburg academic hospital	SAJCH South African Journal of Child Health	i) Pre 2014
Moreira et al.	Accuracy of quick sequential organ failure assessment score to predict mortality in hospitalized patients with suspected infection in an HIV/AIDS reference centre in Rio de Janeiro, Brazil	Clinical microbiology and infection : the official publication of the European Society of Clinical Microbiology and Infectious Diseases	h) All have a syndrome

Mouton et al.	Mortality from adverse drug reactions in adult medical inpatients at four hospitals in South Africa: a cross-sectional survey	British journal of clinical pharmacology	g) No diagnoses
Movahedi et al.	Epidemiology of children with acquired immune deficiency syndrome (stage 3): A referral hospital-based study in Iran	Journal of medical virology	e) Not hospitalised
Moya et al.	Leading causes of death in infants and children under 5 from the champs network	American Journal of Tropical Medicine and Hygiene	e) Not hospitalised
Mucheleng'a nga et al.	The histological appearances of the adult kidney in HIV infection at autopsy at the University Teaching Hospital in Lusaka	Medical Journal of Zambia	h) All have a syndrome
Mudenda et al.	Histopathological Evaluation of Deceased Persons in Lusaka, Zambia With or Without Coronavirus Disease 2019 (COVID-19) Infection: Results Obtained From Minimally Invasive Tissue Sampling	Clinical infectious diseases : an official publication of the Infectious Diseases Society of America	h) All have a syndrome
Mulu et al.	Prevalence of Malnutrition and Associated Factors among Hospitalized Patients with Acquired Immunodeficiency Syndrome in Jimma University Specialized Hospital, Ethiopia	Ethiopian journal of health sciences	e) Not hospitalised
Munthali et al.	Tuberculosis caseload in children with severe acute malnutrition related with high hospital based mortality in Lusaka, Zambia	BMC research notes	f) Everyone has same diagnosis
Mvalo et al.	Antibiotic treatment failure in children aged 1 to 59 months with World Health Organization-defined severe pneumonia in Malawi: A CPAP IMPACT trial secondary analysis	PloS one	h) All have a syndrome
Mwaanza et al.	High rates of congenital cytomegalovirus infection linked with maternal HIV infection among neonatal admissions at a large referral center in sub-Saharan Africa	Clinical infectious diseases : an official publication of the Infectious Diseases Society of America	i) Pre 2014
Mwandama et al.	Prevalence of deep vein thrombosis and associated factors in adult medical patients admitted to the University Teaching Hospital, Lusaka, Zambia	Medical Journal of Zambia	c) Not all PLHIV
Mwangome et al.	Diagnostic criteria for severe acute malnutrition among infants aged under 6 mo	The American journal of clinical nutrition	i) Pre 2014
Nacarapa et al.	Extrapulmonary tuberculosis mortality according to clinical and point of care ultrasound features in Mozambique	Scientific reports	f) Everyone has same diagnosis
Naicker et al.	HIV in acute care: A review of the burden of HIV-associated presentations to an emergency department	South African Family Practice	INCLUDED
Naidoo et al.	HIV prevalence and morbidity in older in-patients in a high HIV prevalence setting	AIDS research and human retroviruses	INCLUDED
Naidoo et al.	HIV Prevalence and Morbidity in Older Inpatients in a High HIV Prevalence Setting	AIDS research and human retroviruses	k) Duplicate record
Nakiyingi et al.	Role of chest X-ray in diagnosis of HIV-associated smear-negative TB in Uganda	Topics in Antiviral Medicine	e) Not hospitalised
Namuju et al.	Rates of refusal of clinical autopsies among HIV-positive decedents and an overview of autopsies in Uganda	Wellcome open research	g) No diagnoses
Nascimento et al.	Mortality in hospitalized HIV-infected patients in a referral center in Bahia, Brazil	The Brazilian journal of infectious diseases : an official publication of the Brazilian Society of Infectious Diseases	l) Cause of admission only
Ndlovu et al.	Changing mortality amongst hospitalised children with Severe Acute Malnutrition in KwaZulu-Natal, South Africa, 2009 - 2018	BMC nutrition	g) No diagnoses
Negera et al.	Clinical outcome of admitted HIV/AIDS patients in Ethiopian tertiary care settings: A prospective cohort study	PloS one	z) Duplicate paper in same study
Negera et al.	Health-related quality of life among admitted HIV/AIDS patients in selected ethiopian tertiary care settings: A cross-sectional study	Open Public Health Journal	g) No diagnoses

Nemi et al.	Infection par le VIH meconnue : circonstances de decouverte et pronostic en milieu de medecine interne a Lome	Journal de la Recherche Scientifique de l‚ÄôUniversit√© de Lom√©	h) All have a syndrome
Neto et al.	HIV treatment non-adherence is associated with ICU mortality in HIV-positive critically ill patients	Journal of the Intensive Care Society	k) Duplicate record
Newberry et al.	Early use of corticosteroids in infants with a clinical diagnosis of Pneumocystis jiroveci pneumonia in Malawi: a double-blind, randomised clinical trial	Paediatrics and international child health	f) Everyone has same diagnosis
Ngari et al.	Mortality during and following hospital admission among school-aged children: a cohort study	Wellcome open research	c) Not all PLHIV
Nicol et al.	Accuracy of a Novel Urine Test, Fujifilm SILVAMP Tuberculosis Lipoarabinomannan, for the Diagnosis of Pulmonary Tuberculosis in Children	Clinical infectious diseases : an official publication of the Infectious Diseases Society of America	h) All have a syndrome
Nijhawan et al.	HIV care cascade before and after hospitalization: impact of a multidisciplinary inpatient team in the US South	AIDS care	INCLUDED
Nijhawan et al.	A Multicomponent Intervention to Reduce Readmissions Among People With HIV	Journal of acquired immune deficiency syndromes (1999)	l) Cause of admission only
Njuguna et al.	Urgent versus post-stabilization art in hospitalized children: A randomized trial	Topics in Antiviral Medicine	k) Duplicate record
Njuguna et al.	Urgent versus post-stabilisation antiretroviral treatment in hospitalised HIV-infected children in Kenya (PUSH): a randomised controlled trial	The lancet. HIV	z) Duplicate paper in same study
Njuguna et al.	Brief Report: Cofactors of Mortality Among Hospitalized HIV-Infected Children Initiating Antiretroviral Therapy in Kenya	Journal of acquired immune deficiency syndromes (1999)	INCLUDED
Njuki et al.	Using verbal and social autopsies to explore health-seeking behaviour among HIV-positive women in Kenya: A retrospective study	BMC Women's Health	e) Not hospitalised
Nozza et al.	Decrease in Incidence Rate of Hospitalizations Due to AIDS-Defining Conditions but Not to Non-AIDS Conditions in PLWHIV on cART in 2008-2018 in Italy	Journal of clinical medicine	INCLUDED
Nurutdinova et al.	Linkage to HIV outpatient care following an inpatient stay	Open Forum Infectious Diseases	g) No diagnoses
Nyakeriga et al.	Mortuary and hospital-based HIV mortality surveillance among decedents in a low-resource setting: lessons from Western Kenya	BMC public health	e) Not hospitalised
Ofem et al.	Effect of task-shifting HIV testing from laboratory personnel to nurses on paediatric and adolescent HIV testing rate and yield in rural Nigeria: a prospective before-and-after study	The Lancet Global Health	g) No diagnoses
Ojobi et al.	Pattern of medical admissions in a tertiary health centre in Makurdi, north central Nigeria: A one year review	Highland Medical Research Journal	g) No diagnoses
Okeng'o et al.	Early Mortality and Associated Factors among Patients with Stroke Admitted to a Large Teaching Hospital in Tanzania	Journal of stroke and cerebrovascular diseases : the official journal of National Stroke Association	f) Everyone has same diagnosis
Okoroiwu et al.	Causes of morbidity and mortality among patients admitted in a tertiary hospital in southern Nigeria: A 6 year evaluation	PloS one	g) No diagnoses
Olack et al.	Causes of mortality in women of reproductive age living in an urban slum (kibera) Nairobi	American Journal of Tropical Medicine and Hygiene	e) Not hospitalised
Oladele et al.	Candidaemia in a tertiary hospital in Nigeria	African journal of laboratory medicine	c) Not all PLHIV
Olds et al.	Non-communicable disease burden among inpatients at a rural district hospital in Malawi	Global health research and policy	INCLUDED
Oliwa et al.	Diagnostic practices and estimated burden of tuberculosis among children admitted to 13 government hospitals in Kenya: An analysis of two years' routine clinical data	PloS one	c) Not all PLHIV

Onyango et al.	Causes of death in HIV-infected and HIV-uninfected children in the child health and mortality prevention surveillance study-Kenya	AIDS (London, England)	e) Not hospitalised
Onyango et al.	Causes of death in HIV-infected and HIV-uninfected children aged under-five years in western Kenya	AIDS (London, England)	e) Not hospitalised
Ordi et al.	Clinico-pathological discrepancies in the diagnosis of causes of death in adults in Mozambique: A retrospective observational study	PloS one	l) Cause of admission only
Oud et al.	Trends of demand for critical care services among hiv-infected patients: A population-based study	Critical Care Medicine	g) No diagnoses
Ousley et al.	High Proportions of Patients With Advanced HIV Are Antiretroviral Therapy Experienced: Hospitalization Outcomes From 2 Sub-Saharan African Sites	Clinical infectious diseases : an official publication of the Infectious Diseases Society of America	l) Cause of admission only
Ozdemir et al.	Evaluation of epidemiological, clinical, and laboratory features and mortality of 144 HIV/AIDS cases in Turkey	HIV clinical trials	e) Not hospitalised
P√©rez Vega et al.	Mortalidad en pacientes hospitalizados con diagn√≥stico de virus de inmunodeficiencia humana	Bol. venez. infectol	INCLUDED
Pan et al.	Viral suppression and HIV transmission behaviors among hospitalized patients living with HIV	International journal of STD & AIDS	g) No diagnoses
Pandharpurkar et al.	Spectrum of opportunistic infections in relation to CD4 counts in HIV/AIDS patients admitted in the department of general medicine of a tertiary care hospital	NA	z) Duplicate paper in same study
Pang et al.	Prevalence of Opportunistic Infections and Causes of Death among Hospitalized HIV-Infected Patients in Sichuan, China	The Tohoku journal of experimental medicine	g) No diagnoses
Parry et al.	HIV-related admissions to a London specialist unit: Who, what and why?	HIV Medicine	a) Irrelevant
Pavlinac et al.	Stool Xpert MTB/RIF and urine lam for diagnosing TB in HIV-infected Kenyan children	Topics in Antiviral Medicine	c) Not all PLHIV
Peet et al.	An audit of HIV testing in general medical patients within 24 hours of admission at a university teaching hospital in North West England	HIV Medicine	g) No diagnoses
Perry et al.	HIV-related mortality at a district hospital in Botswana	International journal of STD & AIDS	i) Pre 2014
Pham et al.	Morbidity and mortality patterns in children admitted to hospital in Thai Binh, Vietnam: A five-year descriptive study with a focus on infectious diseases	Journal of Epidemiology and Global Health	g) No diagnoses
Phillips et al.	Implementation of a routine HIV testing policy in an acute medical setting in a UK general hospital: a cross-sectional study	Sexually transmitted infections	i) Pre 2014
Pirotte et al.	[Emergency department utilization by HIV-positive adults in a Belgian setting]	Etude des motifs d'admission au service des Urgences d'une cohorte de patients infectes par le VIH suivis au CHU de Liege.	INCLUDED
Prakash et al.	Acute kidney injury in patients with human immunodeficiency virus infection	Indian journal of nephrology	h) All have a syndrome
Prendergast et al.	Inflammatory biomarkers in HIV-infected children hospitalized for severe malnutrition in Uganda and Zimbabwe	AIDS (London, England)	i) Pre 2014
Prin et al.	High Sepsis-Related Mortality and Antimicrobial Resistance at a Referral Hospital in Malawi	Journal of the American College of Surgeons	c) Not all PLHIV
Quayson et al.	Ante-mortem and post-mortem diagnoses of pulmonary infections in HIV/AIDS patients in a tertiary hospital in Ghana	Virchows Archiv	h) All have a syndrome
Raberahona et al.	Hospital admission among HIV-patients: Causes and factors associated with AIDS-defining events in a referral tertiary care hospital in Antananarivo, Madagascar	Tropical Medicine and International Health	l) Cause of admission only
Raberahona et al.	Hospitalization of HIV positive patients in a referral tertiary care hospital in Antananarivo Madagascar, 2010-2016: Trends, causes and outcome	PloS one	INCLUDED

Raducan et al.	Dermatological manifestations common in hospitalized HIV patients	BMC Infectious Diseases	h) All have a syndrome
Rafi et al.	A Study on Pattern of Neurological Complications in Human Immunodeficiency Virus Infected Patients Attending a Tertiary Care Center in South Tamil Nadu, India	NA	g) No diagnoses
Raga Almudever et al.	Changing pattern of hospital admissions due to medical conditions in HIV-infected subjects in a European public health care system with free access to antiretroviral treatment	HIV Medicine	INCLUDED
Rajbhandari et al.	HBV/HIV coinfection is associated with greater mortality in hospitalized patients with HBV	Hepatology	i) Pre 2014
Rakislova et al.	Clinico-pathological discrepancies in the diagnosis of causes of death in adults in Mozambique	Virchows Archiv	g) No diagnoses
Rakislova et al.	Mortality due to Cryptococcus neoformans and C. Gattii in Mozambique: An Autopsy Study	Laboratory Investigation	z) Duplicate paper in same study
Randall et al.	Acute kidney injury among HIV-infected patients admitted to the intensive care unit	International journal of STD & AIDS	i) Pre 2014
Raphael et al.	Autopsy findings in HIV/aids patients in lago university teaching hospital: A one year prospective study	Laboratory Investigation	i) Pre 2014
Raubenheimer et al.	The utility of a shortened palliative care screening tool to predict death within 12 months - a prospective observational study in two south African hospitals with a high HIV burden	BMC palliative care	l) Cause of admission only
Rayment et al.	Routine HIV testing on an Acute Admissions Unit (AAU) is feasible and affordable, but a challenge to sustain	HIV Medicine	g) No diagnoses
Rein et al.	Causes of hospitalisation among a cohort of people with HIV from a London centre followed from 2011 to 2018	BMC infectious diseases	z) Duplicate paper in same study
Reinhardt et al.	Aids-defining illnesses at initial diagnosis of HIV in a large guatemalan cohort	Open Forum Infectious Diseases	e) Not hospitalised
Ressler et al.	Non-AIDS cancers contribute to an increasing proportion of deaths in persons living with HIV at a single university-based clinic	Open Forum Infectious Diseases	i) Pre 2014
Ricart et al.	Severe HIV-associated pulmonary tuberculosis, 2006-2016	Journal of the International AIDS Society	i) Pre 2014
Rice et al.	Impact of emergency care training on outcomes and care in a ugandan emergency department	Annals of Emergency Medicine	a) Irrelevant
Richards et al.	Infectious disease consultations at a South African academic hospital: A 6-month assessment of inpatient consultations	Southern African journal of infectious diseases	c) Not all PLHIV
Rico et al.	The presence of a malignant comorbidity is a significant predictor of increased 30-day hospital readmission rates in HIV-1 infected individuals	Journal of AIDS and Clinical Research	i) Pre 2014
Robertson et al.	Mortality among clients in the New York city HIV Care Coordination Program (CCP): incidence and associated clinical factors	Annals of epidemiology	e) Not hospitalised
Roche et al.	Multimorbidity in a large district hospital: A descriptive cross-sectional study	South African medical journal = Suid-Afrikaanse tydskrif vir geneeskunde	INCLUDED
Rogena et al.	A review of completeness, correctness, and order of cause of death statements among decedents with documented causes of death and HIV status at two major mortuaries in Kenya, 2015	Journal of forensic and legal medicine	g) No diagnoses
Rohit et al.	Cardiovascular manifestations of HIV infected patients: results of a single centre study from a tertiary care centre	Journal of Evolution of Medical and Dental Sciences	g) No diagnoses
Rudd et al.	Sepsis presentation, management, and outcomes in adults and children admitted to a rural ugandan hospital: A prospective observational cohort study	American Journal of Respiratory and Critical Care Medicine	h) All have a syndrome

Ruiz et al.	Mortality in patients with acquired human immunodeficiency virus infection hospitalized in an intensive care unit during the period 2017-2019	Scientific reports	z) Duplicate paper in same study
Rukhadze et al.	Causes and outcomes of hospitalizations among HIV positive persons in Georgia's referral institution, 2012-2017	HIV Medicine	l) Cause of admission only
Rukhadze et al.	Causes and outcomes of hospitalizations among people living with HIV in Georgia's referral institution, 2012-2017	International journal of STD & AIDS	h) All have a syndrome
Sabur et al.	Using urine lipoarabinomannan (LAM) in practice: The incremental yield of urine lam and xpert MTB/RIF testing in hospitalized hiv-infected patients	Chest	a) Irrelevant
Sachdeva et al.	Demographic Profile and Clinical Features of Admitted HIV Positive Patients and their Correlation with CD4 Counts in a North India Tertiary Care Hospital	NA	z) Duplicate paper in same study
Sanders et al.	Antimicrobial stewardship program to reduce antiretroviral medication errors in hospitalized patients with human immunodeficiency virus infection	Infection control and hospital epidemiology	g) No diagnoses
Schlabe et al.	Patients with HIV and HCV in intensive care treatment: Admission criteria and trends in the intensive care unit (ICU) at the University Hospital of Bonn 2014-2019	HIV Medicine	INCLUDED
Schlabe et al.	People living with HIV, HCV and HIV/HCV coinfection in intensive care in a German tertiary referral center 2014-2019	Infection	z) Duplicate paper in same study
Schlabe et al.	Analysis of the inpatient care spectrum of patients with HIV infection in the infectiology department of a tertiary referral centre before and during the COVID19 pandemic	HIV Medicine	l) Cause of admission only
Seidenberg et al.	Epidemiology of injuries, outcomes, and hospital resource utilisation at a tertiary teaching hospital in Lusaka, Zambia	African Journal of Emergency Medicine	c) Not all PLHIV
Senoglu et al.	Epidemiological differences and risk factors for hospitalization in people living with HIV in Istanbul, Turkey	International journal of STD & AIDS	i) Pre 2014
Serban et al.	Retrospective analysis of HIV/AIDS deaths recorded in the Clinical Infectious Diseases Hospital, Constanta in the period 01 January 2014-30 June 2016. Epidemiological considerations	BMC Infectious Diseases	l) Cause of admission only
Shahrin et al.	Characteristics and predictors of death among hospitalized HIV-infected patients in a low HIV prevalence country: Bangladesh	PloS one	i) Pre 2014
Siefried et al.	Socioeconomic and psychosocial factors are associated with poor treatment outcomes in Australian adults living with HIV: a case-control study	Sexual health	e) Not hospitalised
Silva et al.	Cytomegalovirus infections in patients with HIV/AIDS in a unit of health of the Amazonian Region, Belem, Para, Brazil	Journal of Medical Microbiology and Diagnosis	i) Pre 2014
Silva et al.	Should we perform the serum cryptococcal antigen test in people living with HIV hospitalized due to a community-acquired pneumonia episode?	International journal of STD & AIDS	a) Irrelevant
Simamora et al.	Clinical signs and laboratory parameters as predictors of mortality among hospitalized human immunodeficiency virus-infected adult patients at tertiary hospital in surabaya	Open Access Macedonian Journal of Medical Sciences	g) No diagnoses
Singh et al.	Neurological manifestations in HIV patients - a hospital-based study of Jawaharlal Nehru Institute of Medical Sciences (JNIMS), Imphal, Manipur	Journal of Evolution of Medical and Dental Sciences	z) Duplicate paper in same study
Singh et al.	Burden of pulmonary diseases among HIV-infected patients: A review of nationwide readmission database	American Journal of Respiratory and Critical Care Medicine	i) Pre 2014
Smaill et al.	In early HIV infection, immediate vs deferred antiretroviral therapy reduced serious illnesses at 3 years	Annals of Internal Medicine	e) Not hospitalised
Smaill et al.	In early HIV infection, immediate vs deferred antiretroviral therapy reduced serious illnesses at 3 years	ACP Journal Club	e) Not hospitalised
Smati et al.	A description of the changing needs of inpatient care for HIV patients in the modern antiretroviral therapy era	HIV Medicine	i) Pre 2014
Soares et al.	Acute kidney injury in HIV-infected children: comparison of patients according to the use of highly active antiretroviral therapy	Jornal de pediatria	i) Pre 2014

Sornum et al.	A retrospective review of admissions-what can we learn?	HIV Medicine	g) No diagnoses
Sossen et al.	"SILVAMP TB LAM" Rapid Urine Tuberculosis Test Predicts Mortality in Patients Hospitalized With Human Immunodeficiency Virus in South Africa	Clinical infectious diseases : an official publication of the Infectious Diseases Society of America	h) All have a syndrome
Souza J√∫nior et al.	Aspectos epidemiol√≥gicos da morbimortalidade pelo v√≠rus da imunodefici√™ncia humana no nordeste brasileiro	Rev. Pesqui. (Univ. Fed. Estado Rio J., Online)	i) Pre 2014
Stone et al.	The relationship of weekend admission and mortality on the public medical wards at a Kenyan referral hospital	International health	g) No diagnoses
Strand et al.	The burden of Anemia and operational challenges in conducting clinical research at a medical ward in Lilongwe, Malawi	Blood	c) Not all PLHIV
Streatfield et al.	HIV/AIDS-related mortality in Africa and Asia: evidence from INDEPTH health and demographic surveillance system sites	Global health action	e) Not hospitalised
Streatfield et al.	Cause-specific childhood mortality in Africa and Asia: evidence from INDEPTH health and demographic surveillance system sites	Global health action	e) Not hospitalised
Su et al.	Use of mycobacterial and fungal blood culture bottle in diagnosis of bloodstream infections in AIDS patients	Chinese Journal of Infection and Chemotherapy	h) All have a syndrome
Sudheer et al.	Predictors for mortality among human immunodeficiency virus infected patients on antiretroviral therapy	Value in Health	g) No diagnoses
Sun et al.	Prevalence of renal impairment among HIV positive patients hospitalized in the first hospital of China medical university	International Journal of Clinical and Experimental Medicine	i) Pre 2014
Sutcliffe et al.	A clinical guidance tool to improve the care of children hospitalized with severe pneumonia in Lusaka, Zambia	BMC pediatrics	f) Everyone has same diagnosis
Tepungipame et al.	Predictors of AIDS-related death among adult HIV-infected inpatients in Kisangani, the Democratic Republic of Congo	The Pan African medical journal	INCLUDED
Thit et al.	The clinical utility of the urine-based lateral flow lipoarabinomannan assay in HIV-infected adults in Myanmar: an observational study	BMC medicine	INCLUDED
Traore et al.	[Epidemiological, clinical and evolving HIV-positive patients referred to the University Hospital of Point G, Bamako, Mali]	Profil epidemioclinique et evolutif de patients VIH positif, referes au CHU du Point G, Bamako, Mali.	i) Pre 2014
Tsega et al.	Determinants of non-adherence to antiretroviral therapy in adult hospitalized patients, Northwest Ethiopia	Patient preference and adherence	e) Not hospitalised
Tshikuka et al.	Severity of outcomes associated to illnesses funded by GFATM initiative and socio demographic and economic factors associated with HIV/AIDS, TB and malaria mortality in Kinshasa Hospitals, DRC	Ethiopian journal of health sciences	g) No diagnoses
Turruelles et al.	Clinical and laboratory findings in patients with late diagnosis of AIDS	Revista Habanera de Ciencias Medicas	z) Duplicate paper in same study
Ueckermann et al.	Characteristics and outcomes of patients admitted to a tertiary academic hospital in Pretoria with HIV and severe pneumonia: a retrospective cohort study	BMC infectious diseases	f) Everyone has same diagnosis
Ulyanova Ya et al.	Clinical and laboratory characterictic of acute HIV-infection in adult residents of novosibirsk region	Jurnal Infektologii	l) Cause of admission only
Umbleja et al.	Prevalence of physical-function impairment and frailty in middle-aged PWH	Topics in Antiviral Medicine	e) Not hospitalised
Umeta et al.	Causes and predictors of hospitalization and in-hospital mortality among HIV/AIDS patients on highly active antiretroviral therapy in secondary and tertiary care hospitals in Oromia Regional State: Multi-center cross-sectional study	HIV and AIDS Review	INCLUDED
Vallabha et al.	Clinical Profile of Surgical Diseases with Emergence of New Problems in HIV+ Individuals	The Indian journal of surgery	i) Pre 2014

Valle Diaz De la Guardia et al.	Pharmacological study of HIV patients entered into the hospital	European Journal of Hospital Pharmacy	INCLUDED
van Schalkwyk et al.	Screening for invasive fungal disease using non-culture-based assays among inpatients with advanced HIV disease at a large academic hospital in South Africa	Mycoses	INCLUDED
Verma et al.	Clinical spectrum of renal disease in hospitalized HIV/AIDS patients: A teaching hospital experience	Journal of family medicine and primary care	g) No diagnoses
Verma et al.	Histological spectrum of renal disease in HIV/AIDS patients with significant proteinuria: An Indian perspective	Journal of family medicine and primary care	g) No diagnoses
Vidal et al.	Asymptomatic cryptococcal antigen prevalence detected by lateral flow assay in hospitalised HIV-infected patients in Sao Paulo, Brazil	Tropical medicine & international health : TM & IH	l) Cause of admission only
Vidal et al.	Combining urine antigen and blood polymerase chain reaction for the diagnosis of disseminated histoplasmosis in hospitalized patients with advanced HIV disease	Medical mycology	g) No diagnoses
Vidal Turruelles et al.	Aspectos cl√≠nicos y de laboratorio en pacientes con diagn√≥stico tard√≠o de SIDA	Revista Habanera de Ciencias M√©dicas	g) No diagnoses
Viktorova et al.	Tuberculosis in patients with hiv infection in a therapeutic hospital in a region with high hiv prevalence	Infektsionnye Bolezni	f) Everyone has same diagnosis
Virata et al.	Cumulative burden of non-communicable diseases predicts COVID hospitalization among people with HIV: A one-year retrospective cohort study	PloS one	f) Everyone has same diagnosis
Vosloo et al.	An assessment of critically ill children admitted to a general high-care unit in a regional hospital in western cape, south africa	SAJCH South African Journal of Child Health	g) No diagnoses
Voznesenskiy et al.	Coexistence of Competing Opportunistic Pathogens in Critically ill Patients with Advanced AIDS: A Case Report and Literature Review	Open AIDS Journal	g) No diagnoses
Wamalwa et al.	Cytomegalovirus Viremia and Clinical Outcomes in Kenyan Children Diagnosed With Human Immunodeficiency Virus (HIV) in Hospital	Clinical infectious diseases : an official publication of the Infectious Diseases Society of America	INCLUDED
Wang et al.	Inappropriate data selection and statistical method lead to overestimated mortality for hospitalised HIV/AIDS patients	Epidemiology and infection	a) Irrelevant
Wang et al.	The burden of serious non-AIDS-defining events among admitted cART-naive AIDS patients in China: An observational cohort study	PloS one	k) Duplicate record
Ward et al.	Chemsex related admissions to a city centre hospital	Sexually Transmitted Infections	e) Not hospitalised
Wen et al.	A 10-year national trend in dialysis-requiring AKI among hospitalized adults with HIV infection	Journal of the American Society of Nephrology	i) Pre 2014
Wetzstein et al.	Disseminated disease due to non-tuberculous mycobacteria in HIV positive patients: A retrospective case control study	PloS one	f) Everyone has same diagnosis
Weze et al.	ASSOCIATION BETWEEN VTE AND CLINICAL OUTCOMES IN HOSPITALIZED PATIENTS WITH HIV	Chest	i) Pre 2014
Wolter et al.	HIV and influenza virus infections are associated with increased blood pneumococcal load: a prospective, hospital-based observational study in South Africa, 2009-2011	The Journal of infectious diseases	f) Everyone has same diagnosis
Wolter et al.	Epidemiology of Pertussis in Individuals of All Ages Hospitalized With Respiratory Illness in South Africa, January 2013-December 2018	Clinical infectious diseases : an official publication of the Infectious Diseases Society of America	h) All have a syndrome
Woodruff et al.	Evaluation of healthcare resource use among human immunodeficiency virus (HIV) patients in a large insured U.S. population	Open Forum Infectious Diseases	e) Not hospitalised

Worodria et al.	Predictors Of Mortality Among Hospitalized Pneumonia Patients In A High Hiv Burden Setting	American Journal of Respiratory and Critical Care Medicine	f) Everyone has same diagnosis
Xiaoxia et al.	Clinical features and risk factors of cytomegalovirus infection in AIDS patients	Chinese Journal of Clinical Infectious Diseases	f) Everyone has same diagnosis
Xie et al.	Anemia and opportunistic infections in hospitalized people living with HIV: a retrospective study	BMC infectious diseases	l) Cause of admission only
Xu et al.	Moderate/Severe hyponatremia increases the risk of death among hospitalized Chinese human immunodeficiency virus/acquired immunodeficiency syndrome patients	PloS one	g) No diagnoses
Xu et al.	Underlying Cryptococcal Diseases and the Correlation With Serum Cryptococcal Antigen Titers in Hospitalized HIV-Infected Patients Screened Positive for Cryptococcal Antigenemia	Frontiers in cellular and infection microbiology	l) Cause of admission only
Yadav et al.	Seroprevalence of HIV, HBV and HCV among the Cadaver Population -- A Jaipur Based Study	Medico-Legal Update	e) Not hospitalised
Yakubi et al.	Audit of HIV testing and Retrospective Cohort analysis of HIV positive Critical Care Patients	Journal of the Intensive Care Society	g) No diagnoses
Yancheva et al.	Etiologic Characteristics of Enterocolitis in Hospitalized HIV-Infected Patients for 3-Year Period (2013-2015)	Acta Medica Bulgarica	h) All have a syndrome
Yazdanpanah et al.	What is the hospital economic burden of hiv in France? Results from an analysis of the national french medical information system database	Value in Health	g) No diagnoses
Yunsu et al.	Epidemiological characteristics of HIV infected Korean: Korea HIV/AIDS Cohort Study	Epidemiology and Health	e) Not hospitalised
Zamfir et al.	A retrospective study concerning specific therapy and evolution of invasive fungal infections diagnosed in the National Institute for Infectious Diseases Prof. Dr. Matei Bals	BMC Infectious Diseases	c) Not all PLHIV
Zar et al.	Tuberculosis Diagnosis in Children Using Xpert Ultra on Different Respiratory Specimens	American journal of respiratory and critical care medicine	h) All have a syndrome
Zetola et al.	Longer hospital stay is associated with higher rates of tuberculosis-related morbidity and mortality within 12 months after discharge in a referral hospital in Sub-Saharan Africa	BMC infectious diseases	f) Everyone has same diagnosis
Zhang et al.	Clinical analysis of 53 AIDS cases infected with Talaromyces marneffei	China Tropical Medicine	z) Duplicate paper in same study
Zhao et al.	A baseline epidemiological study of the co-infection of enteric protozoans with human immunodeficiency virus among men who have sex with men from Northeast China	PLoS Neglected Tropical Diseases	e) Not hospitalised
Zijenah et al.	Comparative performance characteristics of the urine lipoarabinomannan strip test and sputum smear microscopy in hospitalized HIV-infected patients with suspected tuberculosis in Harare, Zimbabwe	BMC infectious diseases	h) All have a syndrome
Zimba et al.	RISK FACTORS AND OUTCOMES OF HIV-ASSOCIATED STROKE IN ZAMBIA	Journal of Stroke and Cerebrovascular Diseases	f) Everyone has same diagnosis
Zinserling et al.	Immediate death causes in HIV infection	Virchows Archiv	INCLUDED
